# Dual-ferroelectric stacked high-performance field-effect transistor

**DOI:** 10.1038/s41467-026-76907-4

**Published:** 2026-08-19

**Authors:** Peng Wang, Biyi Jiang, Wuhong Xue, Jingyuan Qu, Wei Zhao, Tianqi Liu, Yan Han, Rongkai Jiao, Ruilong Yang, Feichi Zhou, Xiaohong Xu

**Affiliations:** 1 Key Laboratory of Magnetic Molecules and Magnetic Information Materials of Ministry of Education, School of Materials Science and Engineering, Shanxi Normal University, Taiyuan, China; 2 Research Institute of Materials Science, Shanxi Key Laboratory of Advanced Magnetic Materials and Devices, Shanxi Normal University, Taiyuan, China; 3 School of Microelectronics, Southern University of Science and Technology, Shenzhen, China

**Keywords:** Ferroelectrics and multiferroics, Ferroelectrics and multiferroics

## Abstract

Ferroelectric transistors have the potential to meet the computing demands of data-intensive applications such as artificial intelligence. However, the existing research lacks functional reconfigurability and multi-task parallel processing capability. Here, we report a dual-ferroelectric stacked transistor that operates through opto-electric dual-mode induced ferroelectric polarization reversal, which features configurable multi-mode functionality encompassing electronic storage, multi-level optical memory, and visual functional emulation. The dual-ferroelectric architecture significantly enhances non-volatile memory performances, demonstrating an ultra-high current on/off ratio, ultra-low dark current and energy consumption, over 8-bit conductance states, multiple analog states and large dynamic range. Furthermore, the simulated artificial visual system enables multi-task parallel processing of static and motion mixed-color information. This work demonstrates that dual-ferroelectric stacked architecture holds potential in parallel processing of multidimensional information.

## Abstract (web summary)

The authors propose a dual-ferroelectric stacked field-effect transistor that enables synergistic improvements of memory and synaptic performance, demonstrating high-precision parallel processing capabilities in mixed-color image recognition tasks.

## Introduction

With advances in artificial intelligence and large-scale model technology, the demand for computing power and energy efficiency in emerging fields such as image recognition, motion detection, and autonomous driving is increasing dramatically^1,2^. In traditional von Neumann-architecture vision processing systems, the physical separation of sensing, memory, and processing units leads to significant energy consumption, time delay, and additional hardware costs^3,4^. By comparison, neuromorphic vision systems with bio-hierarchical architecture offer a potential solution to address the above shortcomings due to their high degree of parallelism, low energy consumption, and adaptive learning properties^3,5–7^.

The retina enables redundancy elimination and feature extraction through preprocessing mechanisms, transmitting only key features to the visual cortex for high-level integration, recognition, and comprehension^8,9^. Various retinal cells collaborate with different functional regions of the visual cortex, progressively integrating information through hierarchical connections to achieve multi-task parallel processing of multiple visual information, such as color, shape, and motion^10–14^. This multi-task parallel processing mechanism enables artificial visual systems to achieve high-precision analysis and multi-information collaborative fusion in complex environments, thereby facilitating more efficient real-time perception and decision-making. Therefore, the in-depth development and design of bioinspired neuromorphic vision devices with multi-task parallel processing capabilities are crucial for constructing efficient, adaptive, and low-power intelligent vision systems.

Machine vision systems based on metal oxides^15–17^, two-dimensional materials^5,9,18–20^, and ferroelectric materials^21–23^ have made significant progress in the recognition of static and moving targets. However, most optoelectronic synapse devices typically lack functional reconfigurability and the parallel processing capability for multidimensional visual information (e.g., color, shape, motion)^21,24–27^, which significantly limits their application potential in the processing of complex visual tasks. Therefore, two aspects need to be explored and developed: firstly, the task-adaptive functional reconfiguration capability, enabling dynamic switching of processing modes based on actual needs; secondly, the parallel processing capabilities and recognition accuracy for static and dynamic mixed-color visual information. Notably, opto-ferroelectric synaptic transistors, with the advantages of multi-modal modulation of polarization plasticity^28^, nonvolatile memory^29,30^, low power consumption^31,32^, and reconfigurability^33–35^, lay the foundation for achieving multi-mode functional reconfigurability and multi-task parallel processing of static/dynamic mixed-color images.

Here, we propose a two-dimensional dual-ferroelectric field effect transistor (dual-FeFET) featuring configurable multi-mode functionality, encompassing electronic storage, multi-level optical memory, logic-in-memory, and visual synapse emulation. The device demonstrates good memory properties, including an ultra-large current-switching ratio of ~ 10^8^, ultra-low dark current of 3 × 10^−15^ A, and over 8-bit photoconductance states. Notably, in a broadband range, the device exhibits good tunability of synaptic behavior, such as the long-term potentiation (LTP) and long-term depression (LTD) behaviors with multiple analog states and large dynamic range (DR), high paired-pulse facilitation (PPF) index (~ 317%), and electrical energy consumption of about 0.4 fJ (excluding the optical input energy and the initial polarization-programming energy). The simulated artificial visual system, emulating the hierarchical structure and information processing mechanisms of the retinal-visual cortex, achieves multi-task parallel processing for color classification and digit recognition at different noise levels. Furthermore, a real-time color motion detection simulation system is implemented using inter-frame difference processing, enabling multi-task parallel processing for color discrimination and trajectory tracking of moving objects.

## Results

**Fig. 1:**
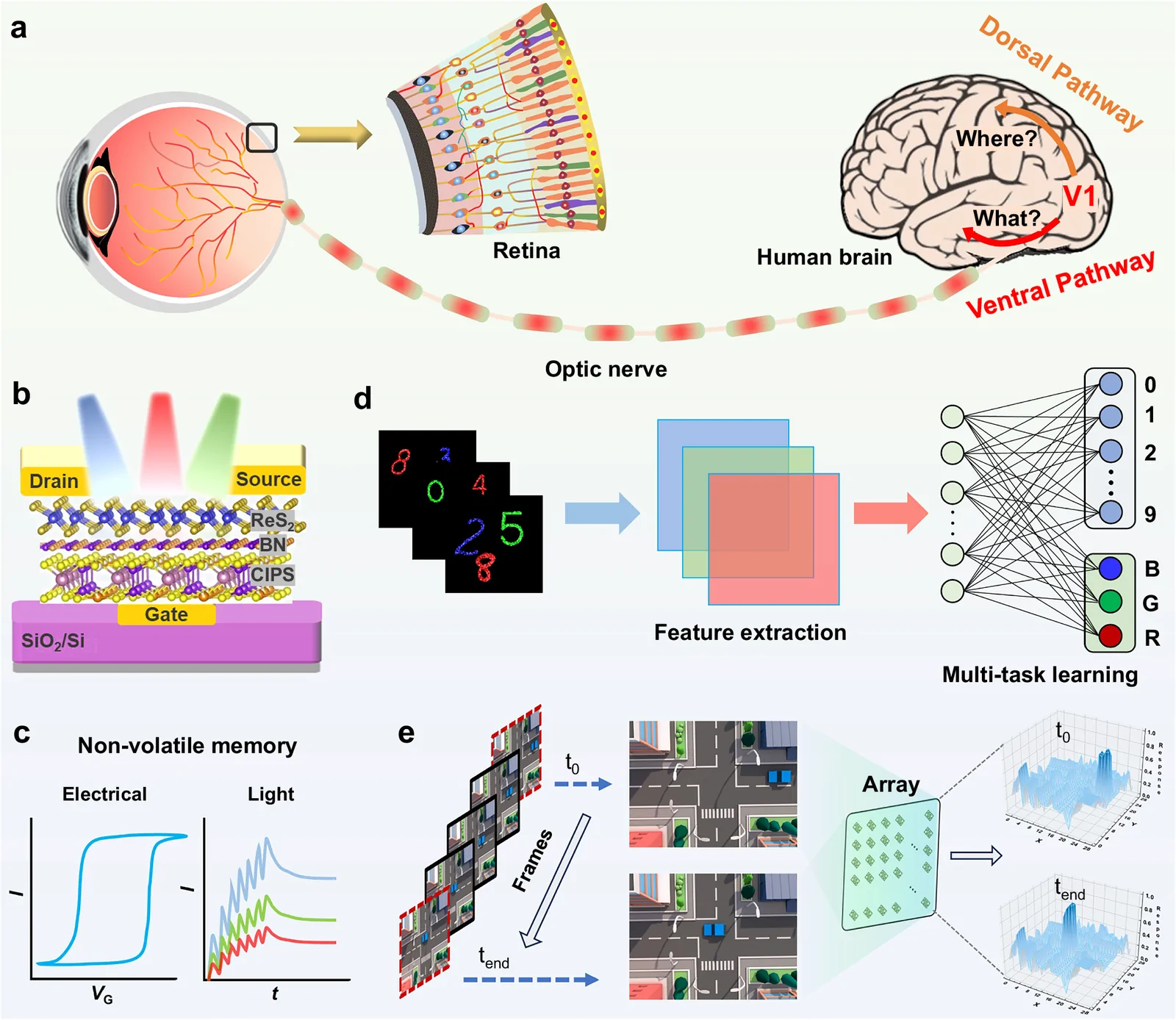
Schematic diagram of human visual system and all-in-one ferroelectric transistor for bioinspired visual system. **a** Schematic structure diagram of the human visual system composed of the retina, optic nerve, and visual cortex. **b** Structural diagram of the fabricated dual-ferroelectric stacked field-effect transistor device. **c**–**e** Schematic diagram of the functional and application demonstration of the device at different operating modes.

The retina efficiently preprocesses information to eliminate redundant information and only transmits key features to the visual cortex, enabling multi-level advanced integration and recognition encompassing color identification, dynamic trajectory analysis, and object recognition (Fig. 1a). We constructed a dual-FeFET to simulate a compact and efficient neuromorphic visual system. The device composition includes a ferroelectric semiconductor channel layer ReS_2_, a dielectric layer BN, and a ferroelectric layer CuInP_2_S_6_ (CIPS) (Fig. 1b and Supplementary Fig. 1) (the specific fabrication process is described in Methods and Supplementary Fig. 2). The ferroelectricity of CIPS and ferroelectric semiconductor properties of ReS_2_ are shown in Supplementary Figs. 3, 4. The high-quality interfaces of the fabricated devices were confirmed by Raman, transmission electron microscopy (TEM), and energy dispersive X-ray spectroscopy (EDS) element mapping (Supplementary Figs. 5, 6). This ferroelectric transistor operates through light-electric dual-mode modulation ferroelectric polarization reversal mechanism, enabling configurable multi-mode functionality, including electronic storage, multi-level optical memory, and static/motion image recognition (Fig. 1c–e).

**Fig. 2:**
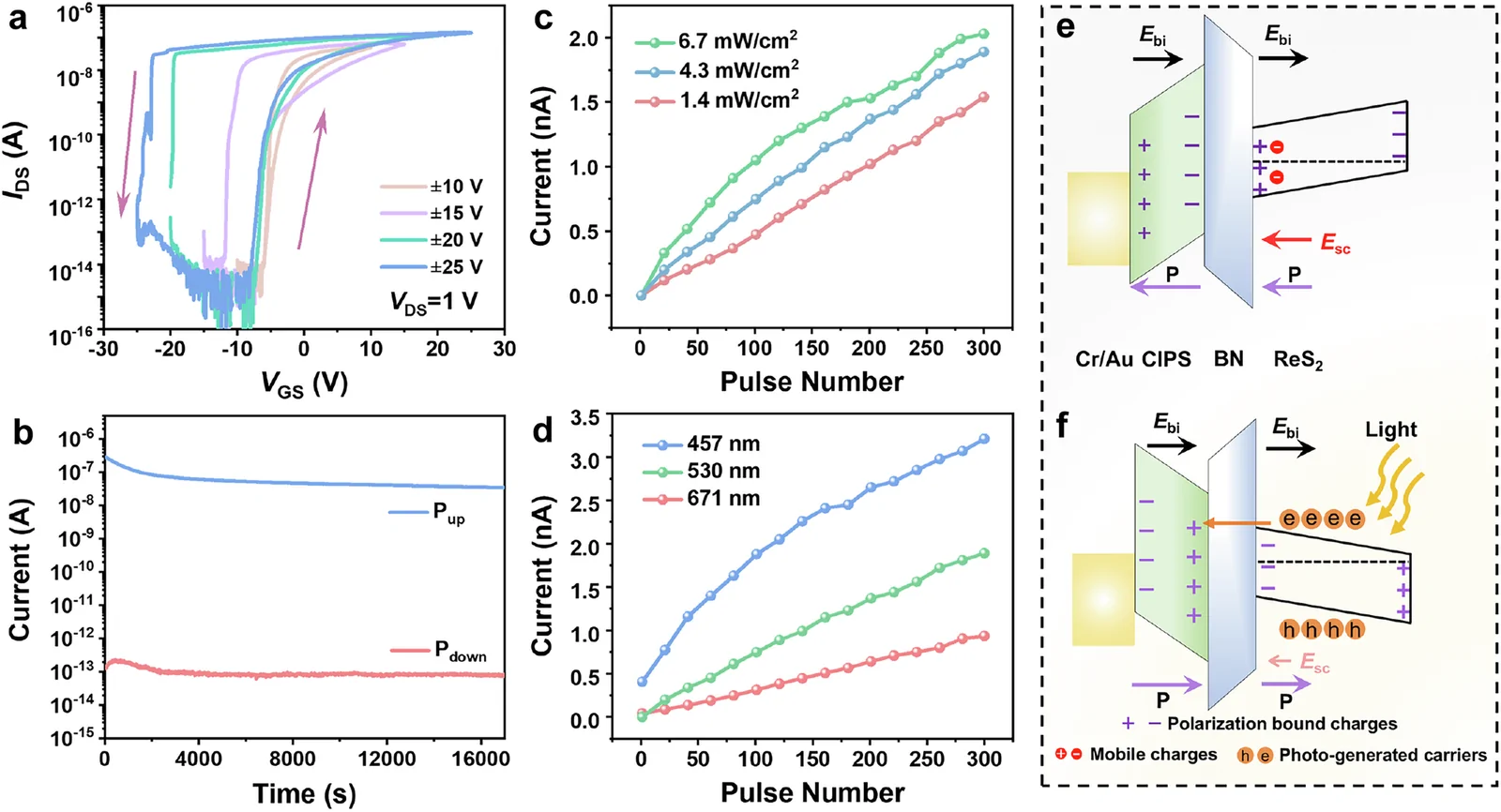
Memory proprieties and energy band diagrams of the ReS_2_/BN/CIPS FeFET. **a** The transfer curves at different sweep ranges and fixed *V*_DS_ of 1 V. **b** Retention characteristic after applying different polarization voltage pulses. **c**,** d** Multi-level memory characteristics with 300 distinguishable conductance states under varying optical conditions: light intensity at a fixed 457 nm wavelength (**c**) and wavelength at a fixed light intensity (**d**). **e**, **f** Band diagrams of light-induced ferroelectric polarization reversal.

Firstly, the electronic memory performances of dual-FeFET were examined. The transfer curves of the device were measured under varying gate voltage sweep ranges (*V*_GS_), with the drain voltage (*V*_DS_) fixed at 1 V (Fig. 2a). The transistor exhibits a typical n-type behavior with anticlockwise hysteresis loop induced by ferroelectric polarization reversal. Meanwhile, the transistor shows a high current on/off ratio of ~ 10^8^ and an ultra-low dark current of ~ 3 × 10^−15^ A, demonstrating a significant potential for applications in non-volatile multi-level storage and low power consumption. The retention characteristic was measured after ferroelectric polarization reversal using ±15 V gate pulses. As shown in Fig. 2b, the currents of the polarization-up and polarization-down states do not change significantly even after 1.6 × 10^4 ^s, indicating good retention performance. The endurance test results show that there is no significant degradation in the dynamic on/off ratio of the device after 6000 cycles (Supplementary Fig. 7), demonstrating good endurance. Temperature-dependent experiments (Supplementary Fig. 8a–d) further indicate that the dual-ferroelectric stacked structure exhibits superior storage performances compared to the single ferroelectric layer. When the temperature exceeded the Curie temperature of CIPS, the storage window and on/off ratio of the transfer curve decreased. When the temperature was above the Curie temperatures of both CIPS and ReS_2_, the memory window and on/off ratio decreased to a negligible level.

The high current on/off ratio and long retention time are preconditions for the realization of multi-level memory^36^. The critical step for realizing multi-level optoelectronic memory is to program the device by applying a negative gate voltage (− 15 V) and then to gradually erase it by periodic light pulses. When applying 20 sets of light pulses (each set consisting of five pulses, 671 nm, 8.3 mW/cm^2^, Δ*t* = 0.3 s) to the device, the channel current exhibits a stable stepwise increase trend (Supplementary Fig. 9a). This trend is still observable even after applying 300 light pulses (Supplementary Fig. 9b), suggesting the device possesses over 300 distinct memory states (> 8 bits). We further conducted the retention tests for optical write operations. First, we applied optical pulses of different durations (100 and 256 pulses) to the device and measured the retention characteristics of the photocurrent. As shown in Supplementary Fig. 10a, the photocurrent remained relatively stable even after 10^4 ^s. Subsequently, we applied 300 consecutive optical pulses to further evaluate the retention characteristics of the multi-level storage states. The results indicate that these 300 conductance states exhibit good retention characteristics, and no significant overlap was observed between adjacent states (Supplementary Fig. 10b). The human visual system, in addition to identifying specific colors, can also suppress or enhance particular colors through the attention mechanism. Given this biological capability, possessing distinguishable multi-wavelength nonvolatile storage is critical for constructing an advanced artificial vision system. The multi-level storage properties under different light intensities at a fixed wavelength and under different wavelengths at a fixed light intensity were also measured, 300 distinguishable memory states were observed in both cases (Fig. 2c, d), which is vitally important for achieving high-efficiency and high-accuracy visual neuromorphic computing. Notably, stimuli of different wavelengths evoke distinguishable photo responses, allowing for the selective extraction of target wavelength signals from complex environments, thereby laying the foundation for constructing neuromorphic vision systems with a selective attention mechanism.

The above-mentioned multi-level photoelectric storage properties primarily stem from two aspects: ⅰ. Large current on/off ratios and long retention times provide the basis for realizing multi-level photoelectric storage. ⅱ. Light pulses with different doses gradually induce ferroelectric polarization reversal^29,37^. The ReS_2_/CIPS heterojunction is essentially asymmetric with preferential upward polarization, thus exists an upward built-in electric field (*E*_bi_) both ReS_2_ and CIPS. Since ReS_2_ is a ferroelectric semiconductor, when the polarization direction is set downward, the negative (positive) screening charges are attracted on the bottom (top) surface, and these screening charges induce a downward electric field (*E*_sc_) that can balance the depolarization field (Fig. 2e and Supplementary Fig. 11a). When the device is exposed to illumination, the photogenerated charges compensate for the screening charges and significantly reduce the electric field. Subsequently, the *E*_bi_ and the depolarization field (*E*_dep_) switch the polarization direction from *P*_down_ to *P*_up_. Simultaneously, the photo-generated carriers screen the downward polarization of CIPS and lead to the polarization reversal driven by *E*_bi_ and *E*_dep_ (Fig. 2f and Supplementary Fig. 11b). It should be emphasized that the light-induced reversal of polarization direction exhibits unidirectionality. To verify the mechanism of light-induced ferroelectric polarization reversal, we conducted photo-assisted PFM measurements to directly observe the changes in ferroelectric polarization domains before and after light illumination. As shown in Supplementary Fig. 12a, the polarization states of CIPS and ReS_2_ were first set to the downward state by applying a + 7 V bias (the red dashed box indicates the ReS_2_/CIPS heterojunction, and the black dashed box indicates the separate CIPS). Subsequently, a light pulse (457 nm, 10.9 mW/cm^2^, and 90 s) was applied, the ferroelectric polarization gradually switched to the upward state (Supplementary Fig. 12b), indicating that light illumination can effectively induce ferroelectric polarization reversal. Notably, the polarization reversal in the ReS_2_/CIPS heterojunction region was faster and more complete, which is predominantly attributed to the generation of more photogenerated charge carriers in this region. Although photo-assisted PFM data provide useful support for light-induced ferroelectric polarization reversal, it should be noted that these results do not completely exclude possible contributions from trapping or photogating effects, especially under the actual pulsed optical conditions used for synaptic operation.

**Fig. 3:**
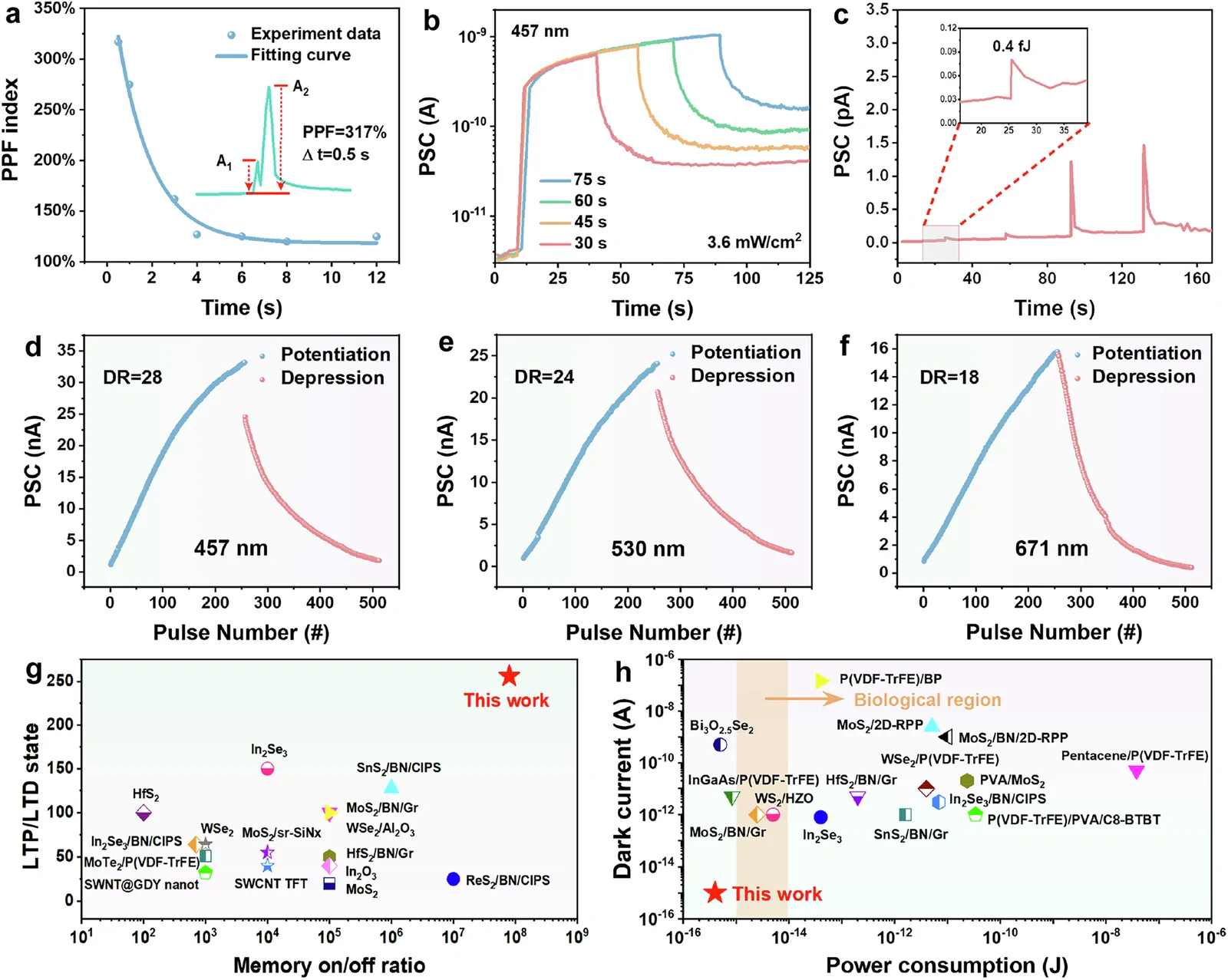
Synaptic behaviors of the ReS_2_/BN/CIPS FeFET. **a** PPF index as a function of the pulse interval time (∆*t*) with a light intensity of 1.3 mW/cm^2^ (the blue curve is the one fitted using a double-exponential decay function). The inset displays PPF triggered by two successive presynaptic spikes with ∆*t* of 0.5 s. **b** Non-volatile photocurrent under different light pulse durations (457 nm, 3.6 mW/cm^2^). **c** Photocurrent evolution under light pulses of varying intensities, and the minimum electrical energy consumption of about 0.4 fJ per pulse (excluding optical input and initial polarization-programming energy consumption; ∆*t* = 0.5 s, *V*_DS_ = 0.01 V). **d**–**f** LTP and LTD behaviors at wavelengths of 457 nm (d), 532 nm (**e**), and 671 nm (**f**), showing the characteristics of multiple analog states and large dynamic range (DR). **g**,** h** Comparison of the analog state numbers and memory on/off ratio (**g**) and the dark current and power consumption (**h**) of our device with those of previously reported optoelectronic synapse devices. Supplementary Tables 1 and 2 provide the detailed references.

Then, the synaptic behaviors of dual-FeFET at different wavelengths were investigated to demonstrate its potential in artificial vision simulations. Figure 3a shows the PPF behavior under the two consecutive light pulses stimulation (671 nm,1.3 mW/cm^2^), and the maximum PPF index reaches 317% (Δ*t* = 0.5 s). The PPF index can be defined by the equation: PPF = (A_2_-A_1_)/A_1 _× 100%, where A_1_ and A_2_ denote the current values of the first and second light pulses, respectively. Meanwhile, the PPF index decreases as increasing Δ*t*, similar to the memory behavior of humans^38^. Synaptic plasticity can be categorized into short-term plasticity and long-term plasticity according to memory duration. The longer retention times and stronger synaptic weights were observed by adjusting the parameters of light pulses at different wavelengths (457, 532, and 671 nm) (Fig. 3b and Supplementary Fig. 13a–c), thus realizing the transition from short-term plasticity to long-term plasticity. Figure 3c demonstrates the postsynaptic currents of the device stimulated by light pulses of different intensities at a voltage of 0.01 V, the minimum electrical energy consumption in the channel during read operation per synaptic event is about 0.4 fJ (excluding the optical input and initial polarization-programming energy consumption) (Supplementary Note 1).

The performance of neural networks (such as CNN, ANN, etc.) is constrained by various factors, including linearity, number of analog states, DR, and cycle to cycle. These factors directly affect the accuracy and stability of neural networks in processing complex tasks^39^. Figure 3d illustrates the LTP and LTD behaviors of dual-FeFET. When 256 consecutive optical pulses (457 nm, 4.3 mW/cm^2^, Δ*t* = 0.5 s) were applied to the device, the 256 distinguishable conductance states and DR of approximately 28 were implemented, which corresponds to the potentiation process. Subsequently, by applying 256 electrical pulses (− 5 V, Δ*t* = 0.5 s), the conductance states gradually decreased with increasing pulse numbers, exhibiting depression behavior. Moreover, the LTP and LTD behaviors were further investigated using optical stimulus of 532 and 671 nm and electrical stimulus of − 5 V. As shown in Fig. 3e, f, the devices also exhibit 256 distinguishable conductance states, with the DR of 24 at 532 nm and 18 at 671 nm. Meanwhile, we quantitatively evaluated the nonlinearity and symmetry of LTP and LTD (Supplementary Fig. 14 and Supplementary Note 2). Under modulation with a wavelength of 457 nm and a voltage of − 5 V, the linearity of potentiation/depression *(*A_p_/A_d_) values are approximately 0.77/0.34, and the asymmetric ratio (AR) is 0.56. For wavelengths of 532 nm and 671 nm, the A_p_/A_d_ values are 1.29/0.39 and 1.99/0.25, respectively, and the AR values are 0.45 and 0.52, respectively. In addition, the cyclic stability of LTP and LTD behaviors over 10 cycles, and smaller cyclic variations can be seen in the curves (Supplementary Fig. 15a–c). Overall, the dual-FeFET device has good performances such as a large on/off ratio, low dark current, multiple analog states, and low power consumption, and these performances are significantly superior to other reported optoelectronic devices (Fig.3g, h and Supplementary Tables 1, 2). These features lay the foundation for subsequent high-precision and high-efficiency recognition of complex visual information. LTP and LTD behaviors were also demonstrated under ± 5 V electrical stimulation (Supplementary Fig. 16). In addition, the reconfigurable logic-in-memory function with “AND” and “OR” are implemented by appropriately adjusting the parameters of the optical pulse input and electrical pulse input (Supplementary Fig. 17a–f and Supplementary Note 3).

In addition, intensity-dependent photocurrent responses were observed under varying wavelengths (457, 532, and 671 nm) (Supplementary Fig. 18a). The photocurrent increases linearly as light intensity rises from 1.1 to 10.9 mW/cm^2^, and the most pronounced enhancement was observed at 457 nm. Meanwhile, through dynamic regulation of ferroelectric polarization switching, the device exhibits tunable photocurrent modulation under positive and negative poling voltages, achieving signal amplification and suppression (Supplementary Fig. 18b).

**Fig. 4:**
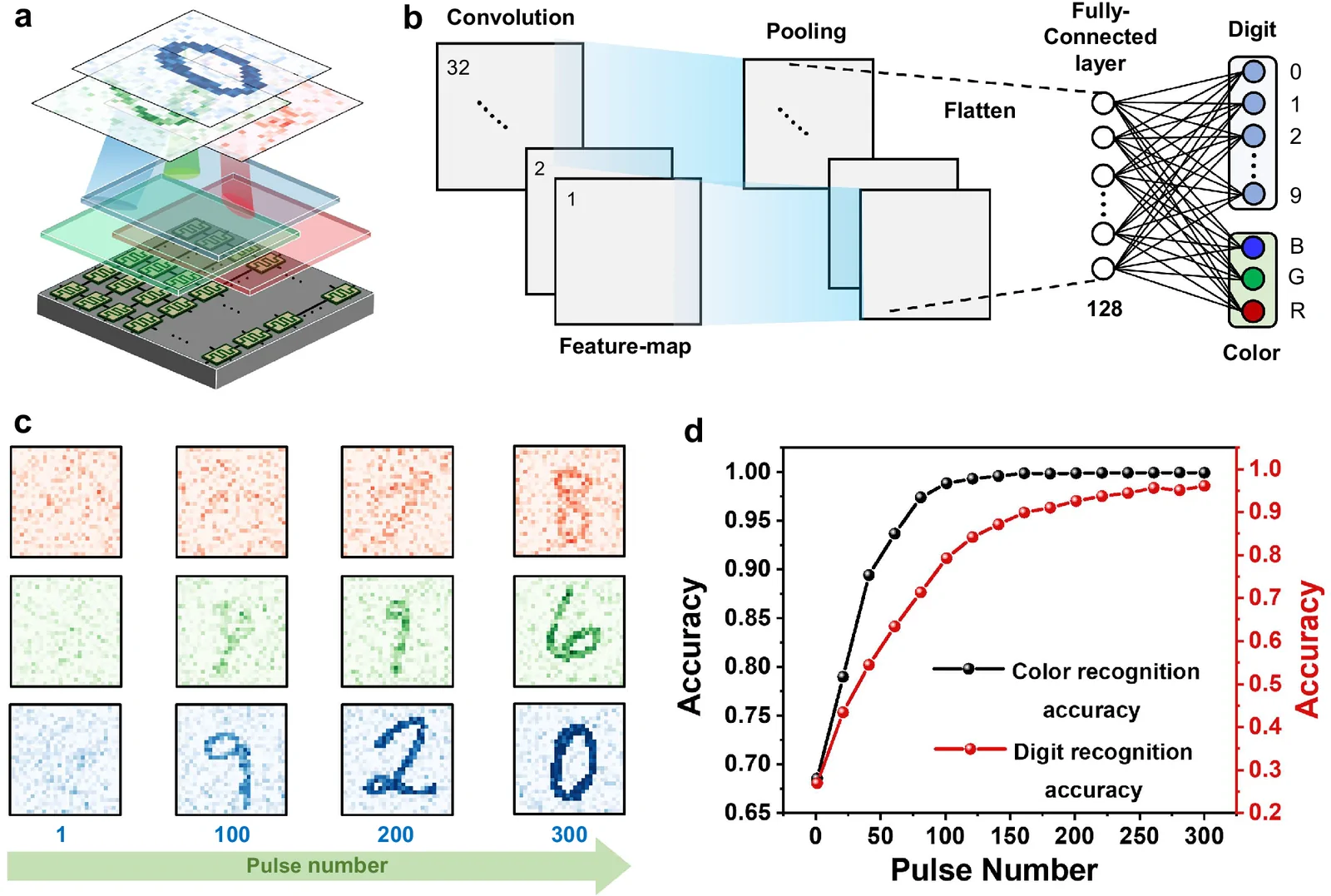
Color and digit class recognition of colored noisy digits. **a** Illustration of a simulated 28 × 28 ferroelectric transistor crossbar array. **b** Architecture of a multi-head convolutional neural network (CNN). **c** Output photocurrent maps of the ferroelectric array in response to noisy colored digit inputs at different perception times (optical pulse numbers) under 15% noise, showing progressively enhanced contrasts. **d** Recognition accuracy of color and digit tasks as a function of optical pulse number (perception time) under 15% noise. The MNIST handwritten digit database^41^ was used for digit recognition tasks.

To enable robust processing of static color visual information while supporting efficient parallel perceptual tasks, we construct a neuromorphic color vision simulation system with multitasking capability. As shown in Fig. 4a, the system consists of a ferroelectric transistor array at the front end, which performs visual sensing and multi-feature enhancement and extraction by emulating biologically inspired temporal integration mechanisms^40^. The quality of extracted features will increase by increasing the perception time. The extracted features are subsequently processed by a multi-head neural network (Fig. 4b), enabling both color and digit recognition.

During operation, colored input images consisting of handwritten digits^41^ embedded in 15% noisy RGB backgrounds (Fig. 4c) are sensed and preprocessed by the ferroelectric transistor array. Owing to its wavelength-dependent, pulse-driven temporal accumulation characteristic, the array outputs photocurrent with enhanced contrast between different colors, enabling progressive color feature enhancement as longer perception time is assigned to the input (Supplementary Fig. 19). In parallel, pulse-driven temporal accumulation enlarges the photocurrent difference between digit signals and fixed background noise, since the photoresponse to digit pixels increases with perception time while photoresponse to noise pixels remains nearly constant, thereby facilitating the extraction of digit appearance features. These dual enhancement effects, including color contrast enhancement and signal-to-noise contrast enhancement, generate high-quality representations that support both color vision and multitasking capability. The resulting current maps are then input into a multi-head convolutional neural network, in which different branches focus on different input features for color and digit recognition. With the pulse-driven temporal integration capability at the front-end array, the constructed neuromorphic color vision system achieves progressively higher accuracy in both color and digit recognition tasks as the perception time increases, owing to the enhanced feature quality provided by the ferroelectric transistor array (Fig. 4d and Supplementary Fig. 20). Furthermore, under other noise conditions, the system also shows a similar trend, achieving gradually improved accuracy as the perception time increases (Supplementary Figs. 21 and 22).

**Fig. 5:**
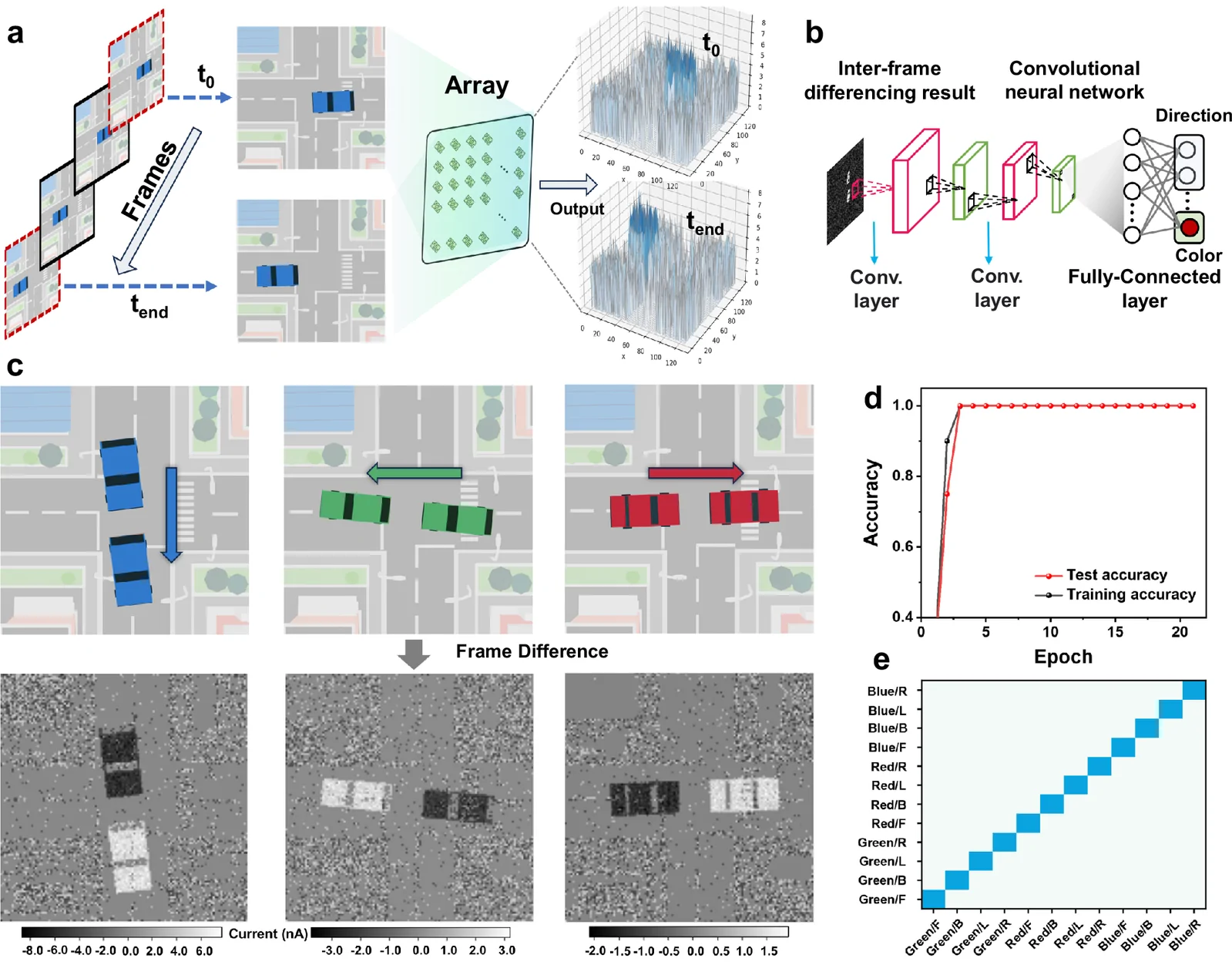
Color and motion recognition of moving colored targets. Schematic of the neuromorphic dynamic vision system consists of (**a**) a dual-FeFET array and (**b**) a CNN network for parallel motion direction and color recognition. **c** Inter-frame differencing results for three colored cars (blue, green, and red) moving in different directions (backward/B, leftward/L, and rightward/R). **d** Recognition accuracy of the network on the training and test sets over training epochs. **e** Confusion matrix of the 12-class classification results on the test set.

Beyond the parallel processing of static features, biological vision systems also support parallel processing of dynamic visual information. Distinct retinal channels extract different attributes and transmit them to the brain, where they are processed in parallel by different neural pathways^14,42^. Inspired by this, to further enable processing of dynamic visual information with multiple perceptual tasks, we construct a neuromorphic dynamic vision system based on a dual-FeFET array capable of simultaneously recognizing object color and motion direction. The system comprises a simulated 128 × 128 dual-FeFET array that extracts both the color and motion features, followed by a convolutional neural network for recognizing the two features, thereby achieving multitasking perception and recognition (Fig. 5a, b).

Dynamic scenes consisting of a moving car with four motion directions and three colors are sequentially sensed by the dual-FeFET array (Supplementary Figs. 23–25). Owing to the wavelength-dependent photoresponse of the device, objects with different colors produce distinguishable photocurrent distributions, enabling effective in-sensor color information encoding. Meanwhile, the intensity-dependent photoresponse allows acquisition of each frame in the dynamic scene, providing the basis for subsequent motion analysis. Motion information is extracted by applying inter-frame differencing to the photocurrent outputs of consecutive frames (see “Methods”), which suppresses static background signals and extracting motion trajectories. As shown in the array output current maps in Fig. 5c, the trajectories of moving objects with different colors are clearly extracted, with distinct current ranges corresponding to different colored cars. The resulting current map are then input into a CNN for simultaneous color and motion direction recognition (Supplementary Figs. 23–26), achieving an overall recognition accuracy of ~ 99% across all scenarios (Fig. 5d, e). Benefiting from the wavelength-and intensity-encoded information provided by the dual-FeFET array at the front end, the constructed neuromorphic dynamic vision simulation system achieves high accuracy in both color and motion direction recognition tasks, enabling efficient parallel processing of dynamic visual information. This work overcomes the limitation of existing studies that can only perform single recognition tasks and effectively improves information processing efficiency, laying the key foundation for building efficient and compact bio-inspired visual devices.

## Discussion

In summary, we propose a ReS_2_/BN/CIPS dual-FeFET for reconfigurable multi-mode function integration leveraging multi-physics field induced ferroelectric polarization reversal mechanisms. The design of the dual-ferroelectric layer stacking significantly improves the non-volatile memory performance and synaptic plasticity. As a non-volatile memory, it exhibits a large current on/off ratio of ~ 10^8^, ultra-low dark current of ~ 3 × 10^−15^ A, retention characteristic exceeding 10^4 ^s, and distinguishable broadband multi-level memory. As a synaptic device, it demonstrates a high PPF index, low power consumption, as well as LTP and LTD behaviors with wide-band, multi-analog states, and large DR. In addition, by constructing a simulated bionic artificial visual system, multi-task parallel recognition of static/dynamic color information is achieved, significantly enhancing the processing efficiency of visual information. This study proposes a neuromorphic parallel processing framework based on two-dimensional dual-FeFET, opening up new avenues for high-precision cognitive computing in complex visual scenes.

## Methods

### Device preparation

Firstly, Cr/Au (10 nm/50 nm) metal electrodes are deposited on SiO_2_ (285 nm)/Si substrates using photolithography technology and electron beam evaporation process. Secondly, CIPS, h-BN, and ReS_2_ flakes were mechanically exfoliated from bulk single crystals (SixCarbon Technology ShenZhen) and attached onto PDMS mediation. Finally, these flakes were sequentially and accurately transferred to the previously pre-deposited Cr/Au metal electrodes using a high-precision dry transfer technique. The pre-deposited electrode process effectively avoids the impact of organic solvents on the material and alleviates the pinning effect at the material-electrode interface.

### Materials and device characterizations

The morphology and thickness of the nanosheets and heterojunction devices were characterized using an optical microscope (AOSVI, L100-HK830) and an atomic force microscope (Dimension Icon, Bruker). The ferroelectric properties of CIPS and ReS_2_ materials were characterized using a piezoelectric force microscope (Dimension Icon, Bruker). Raman spectra were obtained using a confocal Raman system (Horiba Labarum HR Evolution) equipped with a 532 nm laser source. Absorption spectra were recorded using a UV-Vis spectrophotometer (Metatest, MStarter ABS), and second harmonic generation spectra was conducted using MStarter SHG microscope equipped with the 1064 nm laser source. Electronic and optoelectronic measurements were performed using a semiconductor analyzer (Keithley 4200SCS) equipped with a vacuum probe station (TTPX, Lakeshore). The incident light with wavelengths of 457, 532, and 671 nm is obtained from commercial light-emitting diode lamps, whose light intensities were measured by a FieldMate+PM150X photometer.

### Preparation of datasets for static scenario

For the static-colored digit recognition task, the MNIST dataset was used to generate noisy colored digit datasets. The MNIST images have a size of 28 × 28 pixels, corresponding to the simulated 28 × 28 array. The dataset was randomly divided into three equal subsets, and each subset was assigned to one color channel, including red, green, or blue. The dataset contains 60000 training samples and 10000 testing samples.

For each noise level and optical pulse number, an independent dataset was generated with the following sequence (Supplementary Fig. 27a). The colored MNIST image was first binarized. An overall Gaussian noise term was then added to each pixel of the binarized input image intensity to simulate the selected overall noise level:

1 $${P}_{{noise}}\left(x,y\right)=P\left(x,y\right)+N\left(0,{\sigma }_{{overall}}\right)\,$$

where P(x,y) is the binarized input image intensity at pixel position (x,y), and ${\sigma }_{{overall}}$ is the selected overall noise level. This overall Gaussian noise term represents the combined overall noise level in the static dataset, including possible environmental, device-related, and circuit-related contributions. The noisy image intensity was then mapped to a simulated array output current map using the measured device current response versus optical pulse count across RGB wavelengths (Supplementary Fig. 27b). The resulting simulated array output current maps were used for multi-head convolutional neural network training and testing for color and digit recognition.

### Preparation of datasets for dynamic scenario

A dynamic colored-car motion dataset was constructed to simulate dynamic visual scenes of a moving vehicle. For each condition, a colored car (red, green, or blue) was generated in synthetic scenes and programmed to move along one of four motion directions (forward, backward, leftward, and rightward), with variations in motion. These rendered scenes formed a 12-class dataset defined by the combination of three colors and four motion directions. Each class contained 100 distinct samples (1200 samples in total), which were split into training and test sets with a 4:1 ratio.

For both the training and test datasets, the same sequence was used to generate the simulated array output as one dataset sample (Supplementary Fig. 27c). For each dynamic scene, the frame at the beginning of the motion and the frame at the end of the motion were used as the two array input images. Each frame was first downsampled to 128 × 128 pixels to match the size of the simulated array. The corresponding color channel was then extracted according to the object color, and the extracted color-channel image was used as optical input for the simulated array. After extracting this selected color-channel image, pixel-wise environmental noise was added to the image:

2 $${P}_{\lambda }^{{\prime} }\left(x,y\right)={P}_{\lambda }\left(x,y\right)+N\left(0,{\sigma }_{{{{\rm{env}}}}}\right)$$

where ${P}_{\lambda }(x,y)$ is the input image intensity of the selected color channel at pixel position $\left(x,y\right)$, $N(0,{\sigma }_{{{{\rm{env}}}}})$ represents the environmental noise, and ${P}_{\lambda }^{{\prime} }(x,y)$ is the noisy input image intensity. The environmental Gaussian noise was used to represent background and illumination fluctuations in the optical input frames. The current of each simulated pixel was obtained from the measured wavelength- and intensity-dependent device response by linear interpolation:

3 $${I}_{0}(x,y)={f}_{\lambda }[{P}_{\lambda }(x,y)]$$

where ${P}_{\lambda }\left(x,y\right)$ is the intensity of the selected color channel at pixel position $\left(x,y\right)$, ${f}_{\lambda }$ is the linearly interpolated wavelength-dependent intensity-to-current mapping function constructed from the measured discrete data for wavelength λ, (Supplementary Fig. 27d) and ${I}_{0}(x,y)$ is the mapped device output current.

Device noise and other circuit-related noise were further added to the simulated current maps before inter-frame differencing:

4 $${I}_{{{{\rm{sim}}}}}\left(x,y\right)={I}_{0}\left(x,y\right)\left[1+{{{\mathcal{N}}}}\left(0,{\sigma }_{n}\right)\right]$$

where ${\sigma }_{n}$ is the normalized standard deviation.

The above procedure was applied both to the initial and final frames, generating two simulated array current maps. The inter-frame difference was then calculated as:

5 $$\Delta I(x,y)={I}_{{{{\rm{final}}}}}(x,y)-{I}_{{{{\rm{initial}}}}}(x,y)$$

where ${I}_{{{{\rm{initial}}}}}(x,y)$ and ${I}_{{{{\rm{final}}}}}(x,y)$ are the simulated current maps corresponding to the beginning and end frames, respectively. The resulting current difference map was used as one simulated array output sample for CNN training and testing.

### Neural network simulation

A convolutional multi-head neural network was employed for parallel color and digit recognition. The network consists of a single convolutional layer with 32 output channels (3 × 3 kernel, padding 1), followed by a 2 × 2 max pooling layer. The resulting feature maps are flattened and passed through a fully connected layer with 128 neurons, which subsequently branches into two output heads, including a 3-class head for color classification (R/G/B) and a 10-class head for digit recognition (0–9). The model was trained using the Adam optimizer with a learning rate of 1 × 10^−3^ and a batch size of 64.

A convolutional neural network was employed for object color and motion direction. The network comprises two convolutional layers with 8 and 16 output channels, respectively, each employing 3 × 3 convolutional kernels, followed by a max pooling layer. The extracted features are flattened and fed into a fully connected layer with 12 output neurons, corresponding to the combined color-direction classes. The model was trained using the Adam optimizer (learning rate 1 × 10^−3^) with a batch size of 32, and cross-entropy loss was used as the loss function.

The array-level demonstrations in this work are based on simulations parameterized by measured single-device characteristics, and Supplementary Note 4 and Supplementary Fig. 28 describe the proposed FeFET array configuration and operating scheme.

## Supplementary information

Supplementary Information

Transparent Peer Review file

## Source data

Source Data

## Data Availability

The dataset of the main fi gures generated in this study is provided in the Source/Source Data file. Source data are provided in this paper.
