# Supplementary material for: Dual-ferroelectric stacked high-performance field-effect transistor
Source: Nat Commun. 2026 Aug 19;17:9905. doi: 10.1038/s41467-026-76907-4 (PMC13582962; doi:10.1038/s41467-026-76907-4)
Supplement: Supplementary file 1 [file 41467_2026_76907_MOESM1_ESM.pdf]

Supplementary Information

Dual-ferroelectric stacked high-performance field-effect transistor

Peng Wang[1,2,#], Biyi Jiang[3,#], Wuhong Xue[1,2*], Jingyuan Qu[1], Wei Zhao[1], Tianqi Liu[1], Yan Han[1], Rongkai Jiao[1], Ruilong Yang[1,2], Feichi Zhou[3*], Xiaohong Xu[1,2*]

[1]Key Laboratory of Magnetic Molecules and Magnetic Information Materials of Ministry of Education, School of Materials Science and Engineering, Shanxi Normal University, Taiyuan 030031, China

[2]Research Institute of Materials Science, Shanxi Key Laboratory of Advanced Magnetic Materials and Devices, Shanxi Normal University, Taiyuan 030031, China

[3]School of Microelectronics, Southern University of Science and Technology, Shenzhen 518000, China

[#]These authors contributed equally: Peng Wang, Biyi Jiang

[*]Corresponding authors: Wuhong Xue, xuewuhong@sxnu.edu.cn; Feichi Zhou, zhoufc@sustech.edu.cn; Xiaohong Xu, xuxh@sxnu.edu.cn

Supplementary Information includes:

Supplementary Figures 1 to 28

Supplementary Notes 1 to 4

Supplementary Tables 1 to 2

Supplementary References

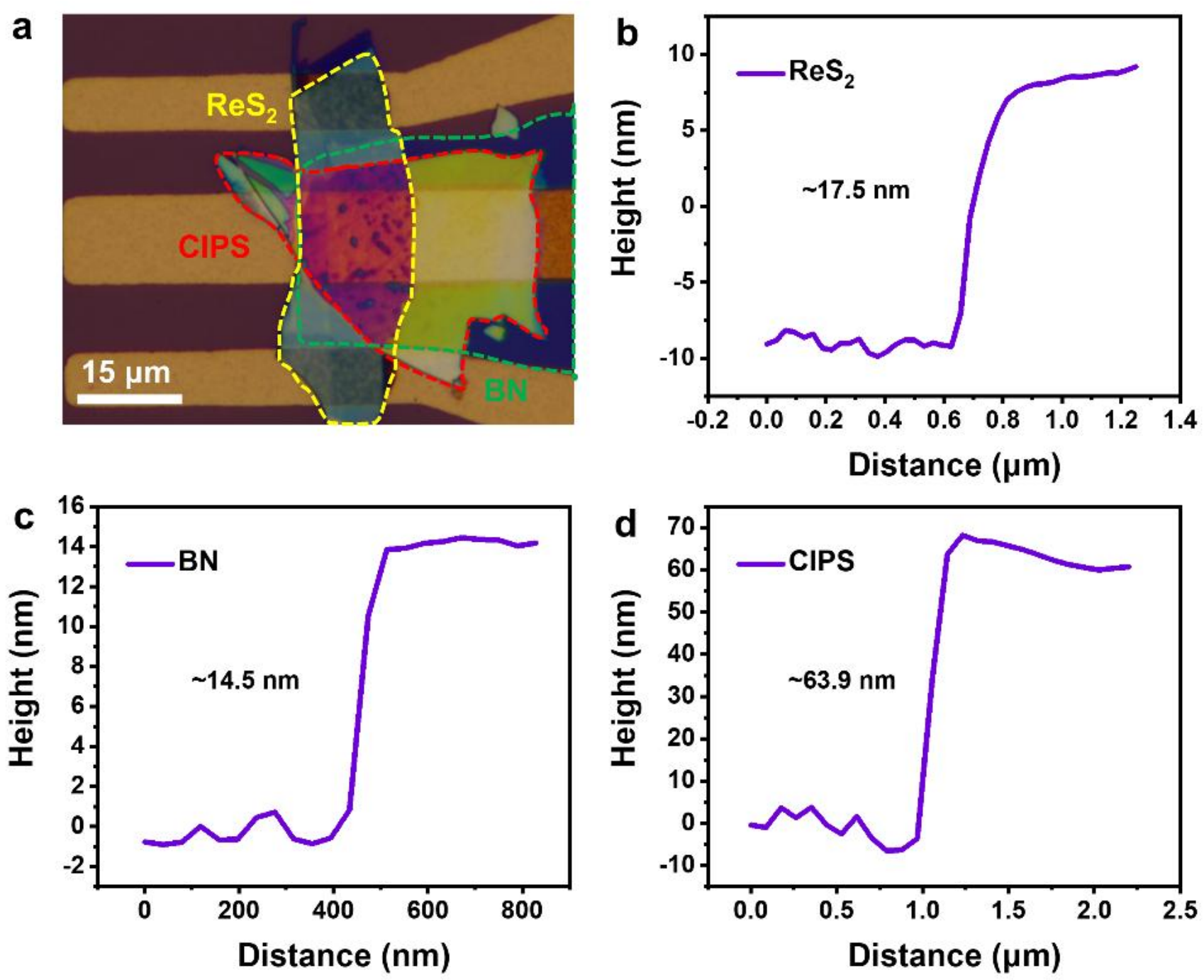

Supplementary Fig. 1 a Optical image of ReS_2/BN/$CuInP_2S_6$ (CIPS) Fe-FET, where the ferroelectric semiconductor channel layer ReS_2, dielectric layer BN, and ferroelectric insulating layer CIPS are marked by red, green, and blue dashed lines, respectively. **b–d** Height profiles of ReS_2, BN, and CIPS nanosheets, with thicknesses of approximately 17.5, 14.5, and 63.9 nm, respectively.

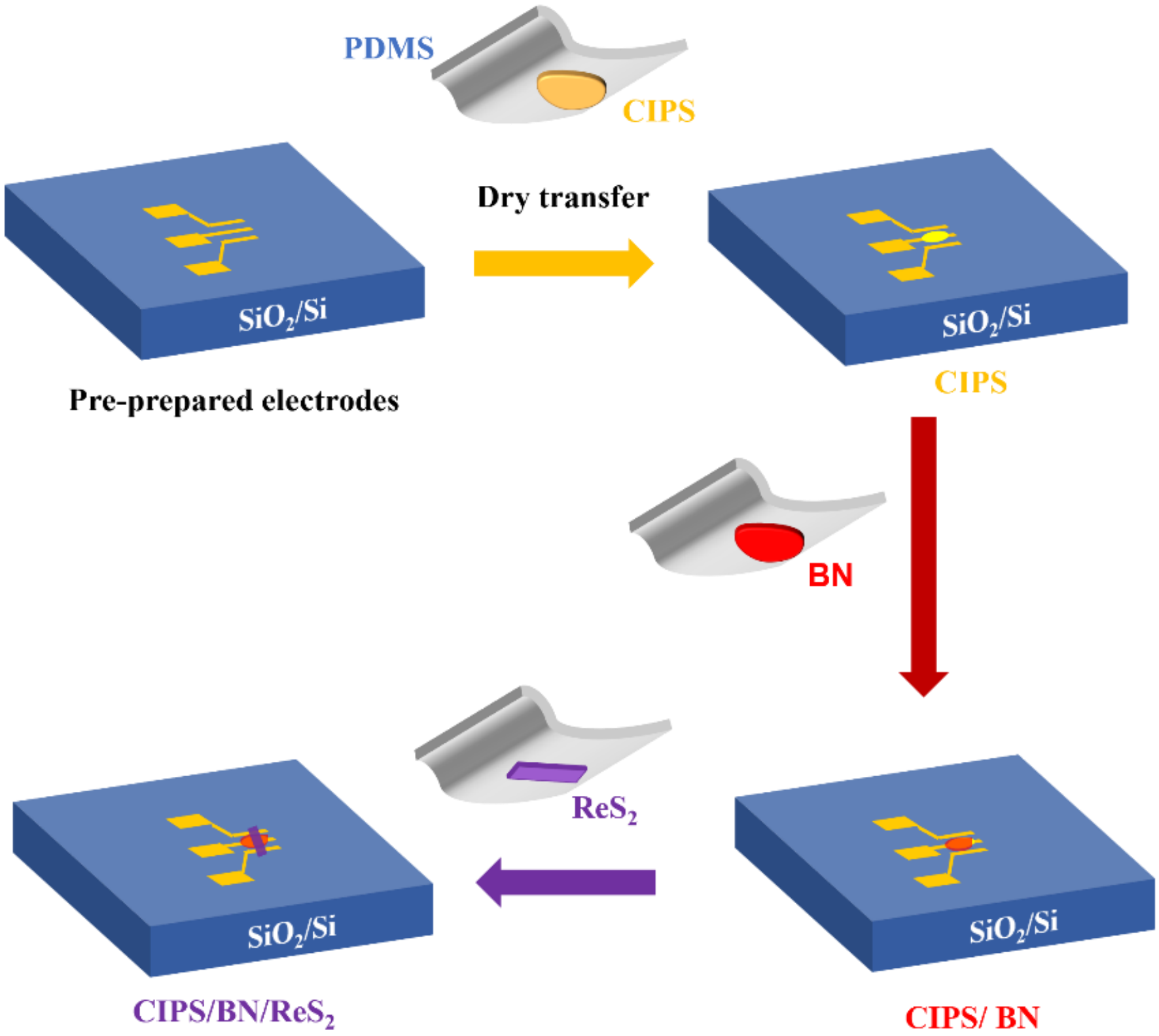

Supplementary Fig. 2 Fabrication process of the dual-ferroelectric field-effect transistors.

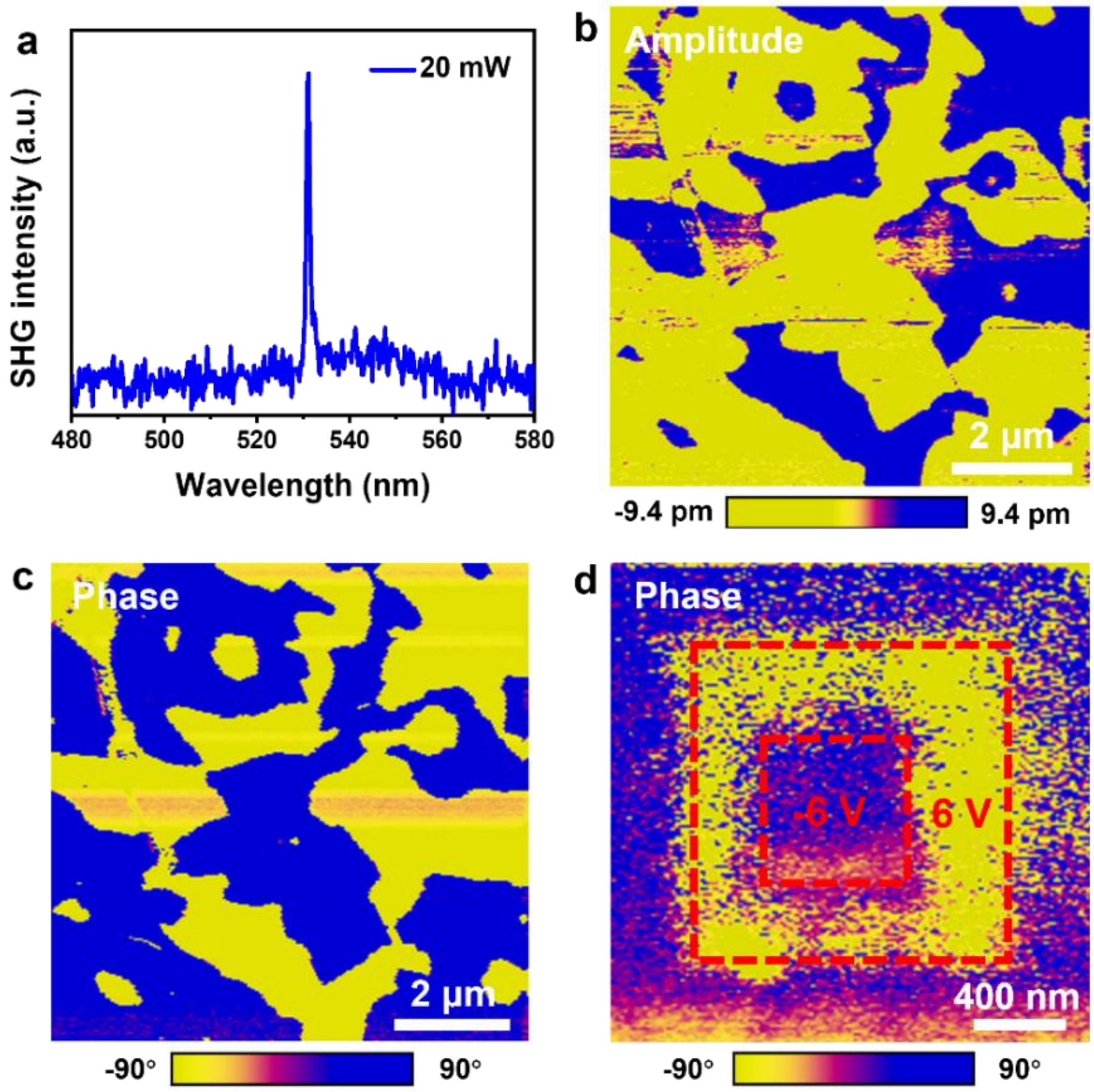

Supplementary Fig. 3 a SHG signal of CIPS nanosheets. **b, c** Local amplitude and phase images of CIPS nanosheets, showing obvious out-of-plane ferroelectric properties. **d** PFM phase of CIPS nanosheets after applying ±6 V polarization voltage.

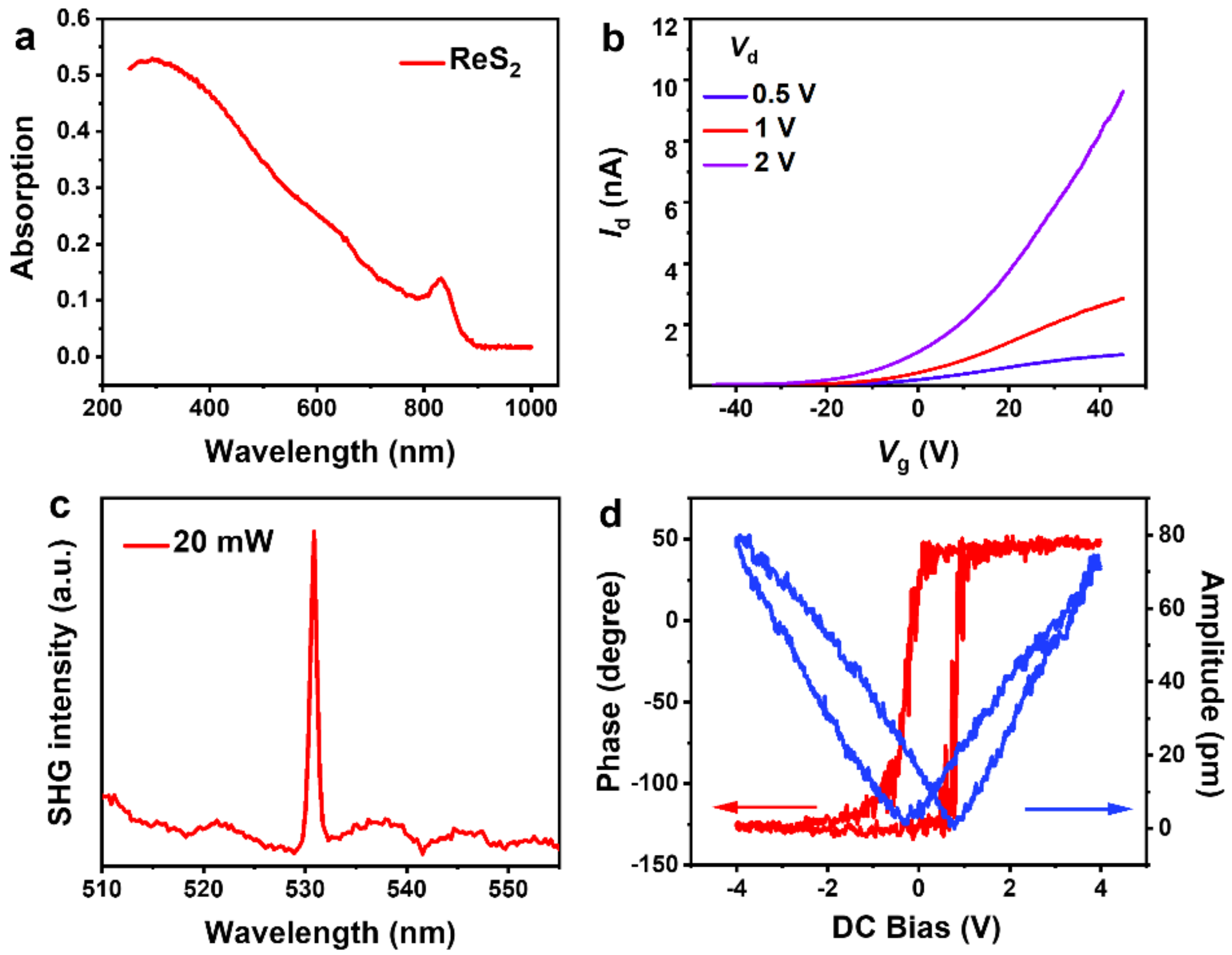

Supplementary Fig. 4 **a** UV-Vis-NIR absorption spectrum of ReS_2 nanosheet with bandgap of ~1.49 eV according to the formula $E_g \approx 1240/\lambda$. **b** Transfer curves of ReS_2 field-effect transistor under different V_{DS} and fixed sweep range of ±45 V. **c** SHG signal of ReS_2 nanosheet. **d** Corresponding PFM amplitude (blue) and phase (red) hysteresis loops of ReS_2 nanosheet.

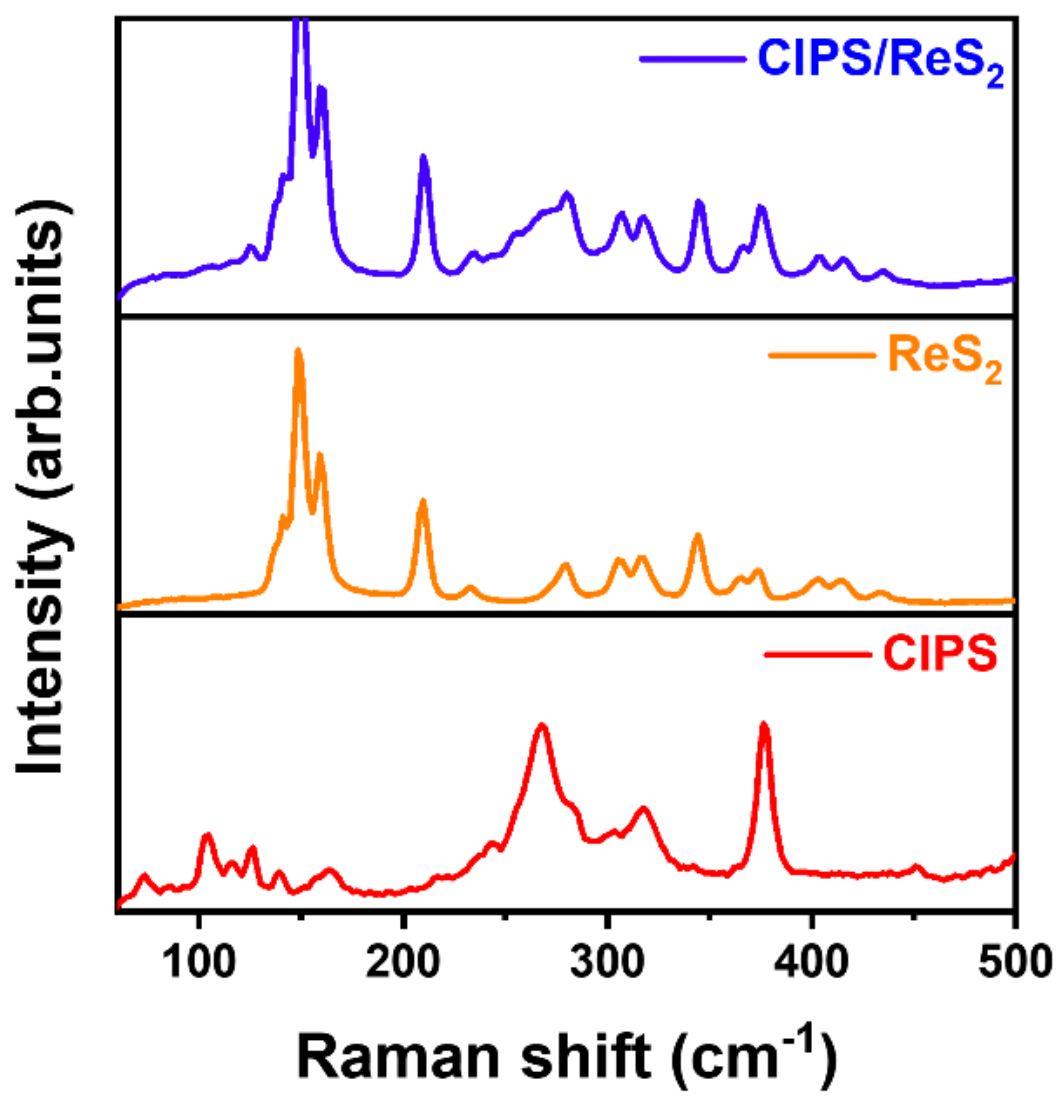

Supplementary Fig. 5 Raman spectra of dual-ferroelectric field-effect transistor.

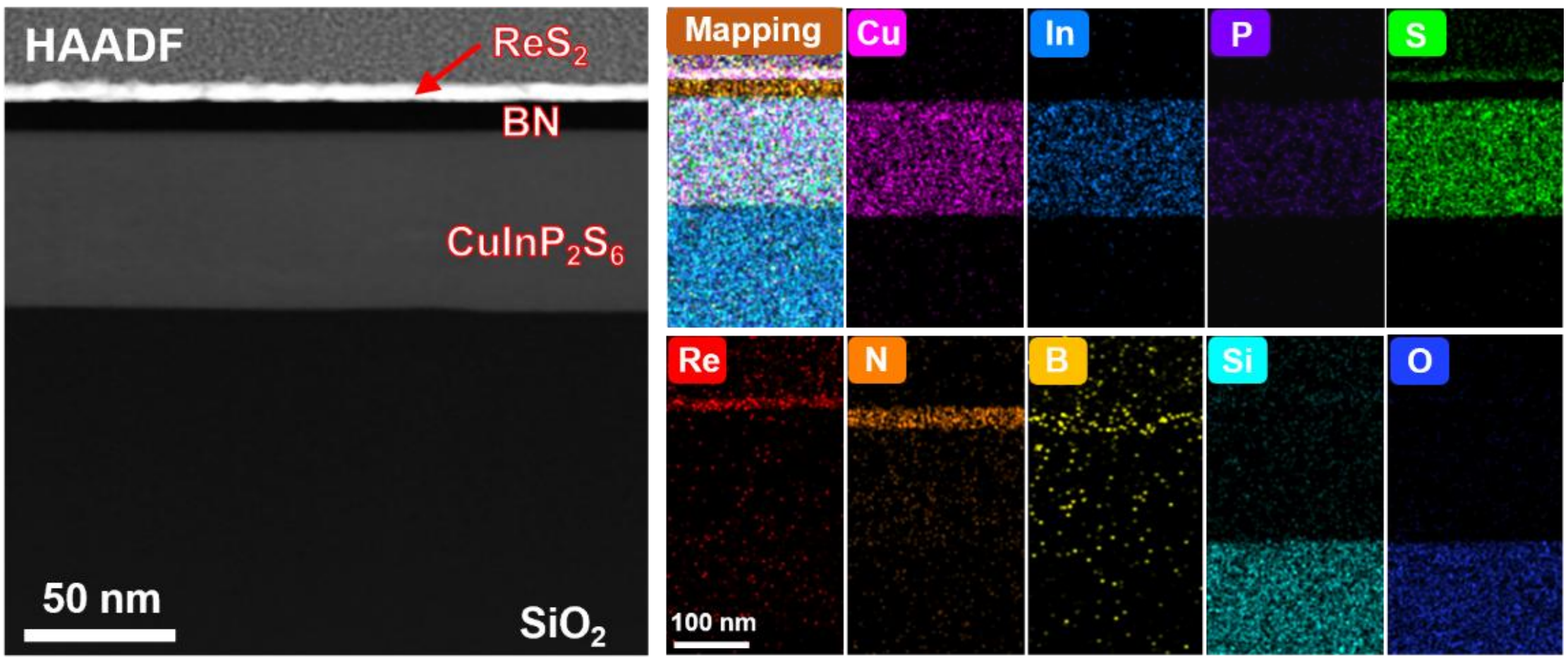

Supplementary Fig. 6 Cross-sectional TEM image and energy dispersive X-ray spectroscopy (EDS) element mapping of ReS_2/BN/CIPS heterojunction device.

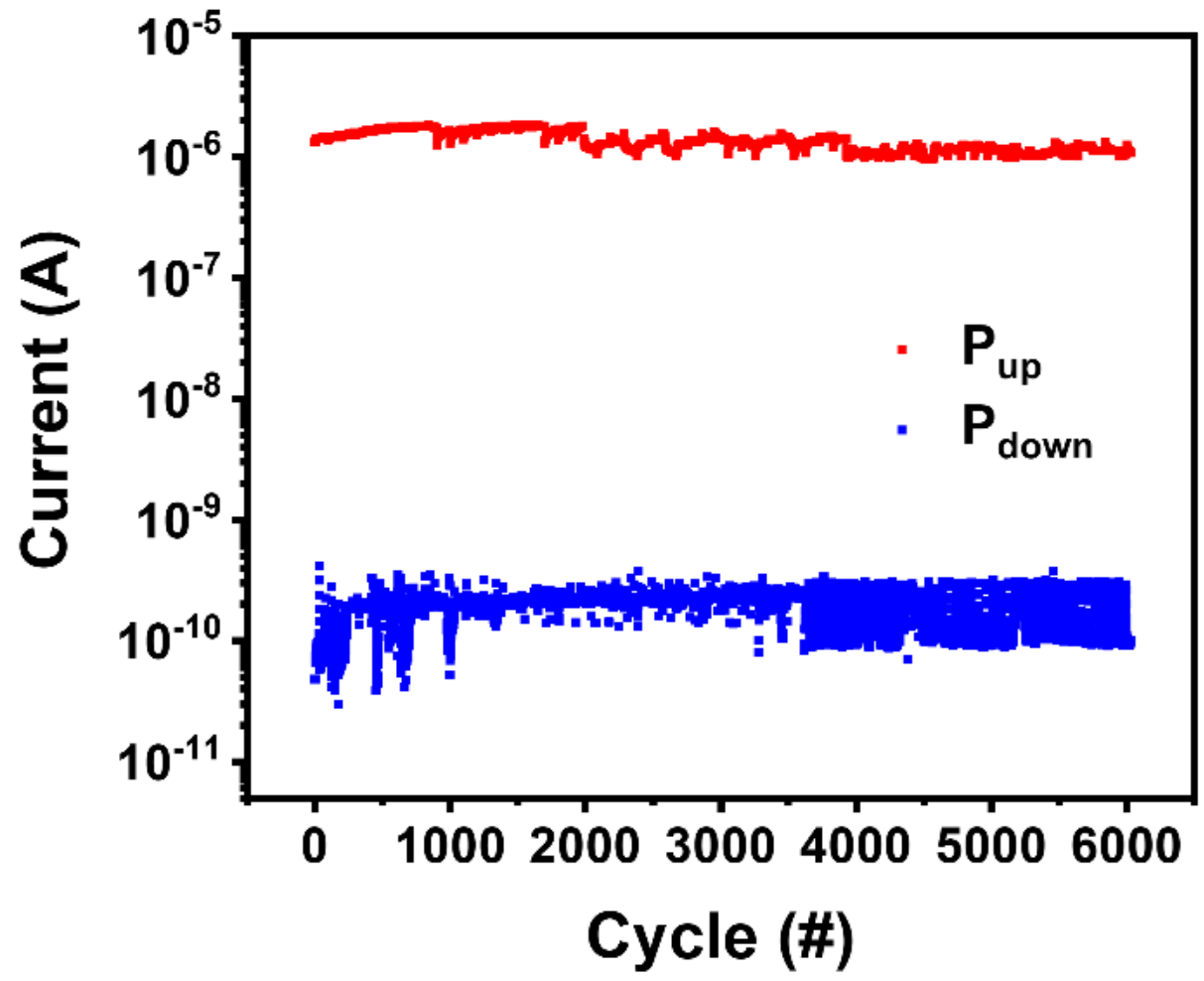

Supplementary Fig. 7 The robust endurance of ReS_2/BN/CIPS Fe-FET after over 6000 erase/write cycles.

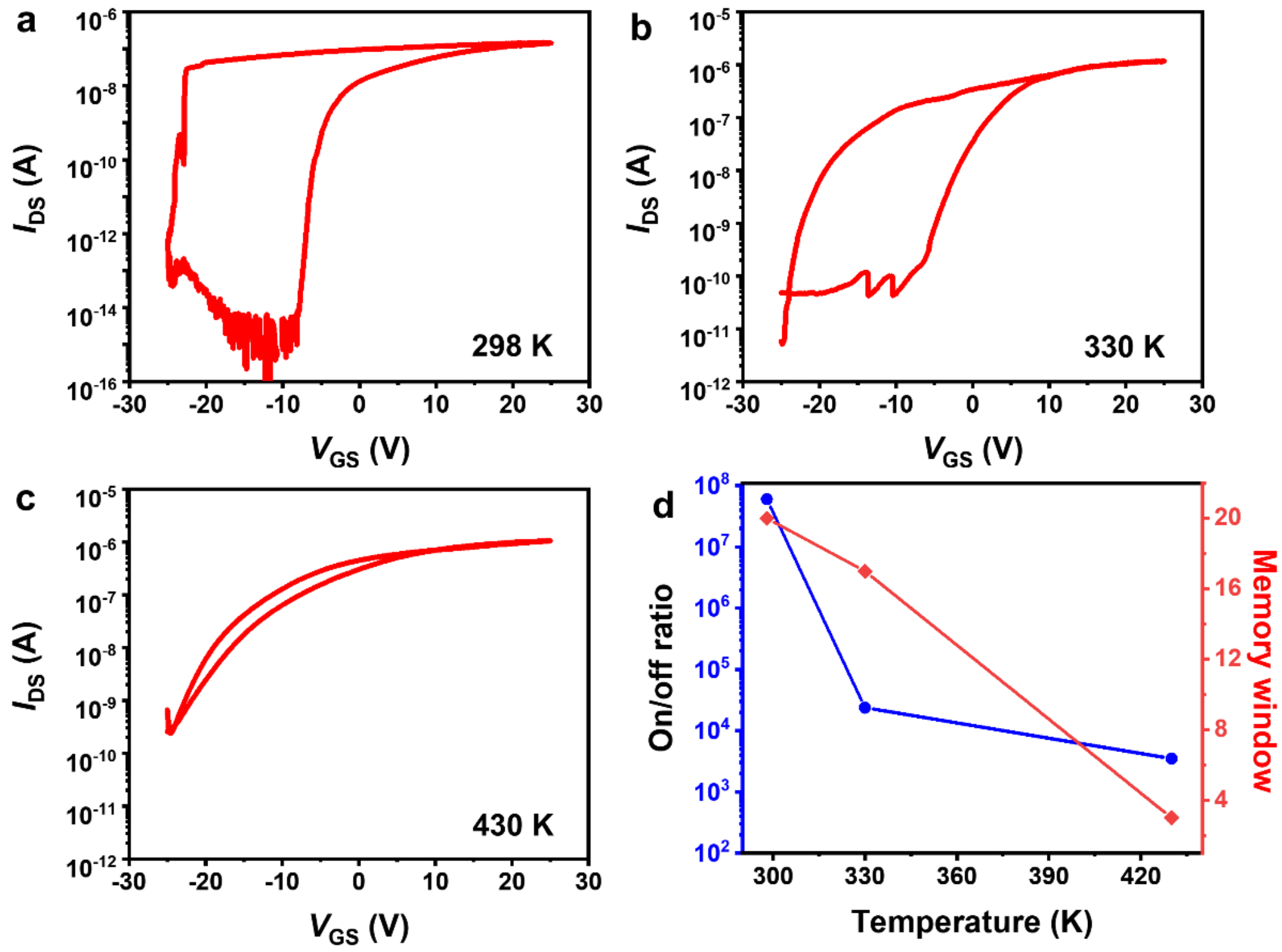

Supplementary Fig. 8 a-c I_{DS}-V_{GS} transfer curves of ReS_2/BN/CIPS Fe-FET at different temperatures, V_{DS}=1 V. **d** The dependence of the on-off ratio and memory window on temperature. As the temperature increases, the on/off ratio and memory window decrease significantly.

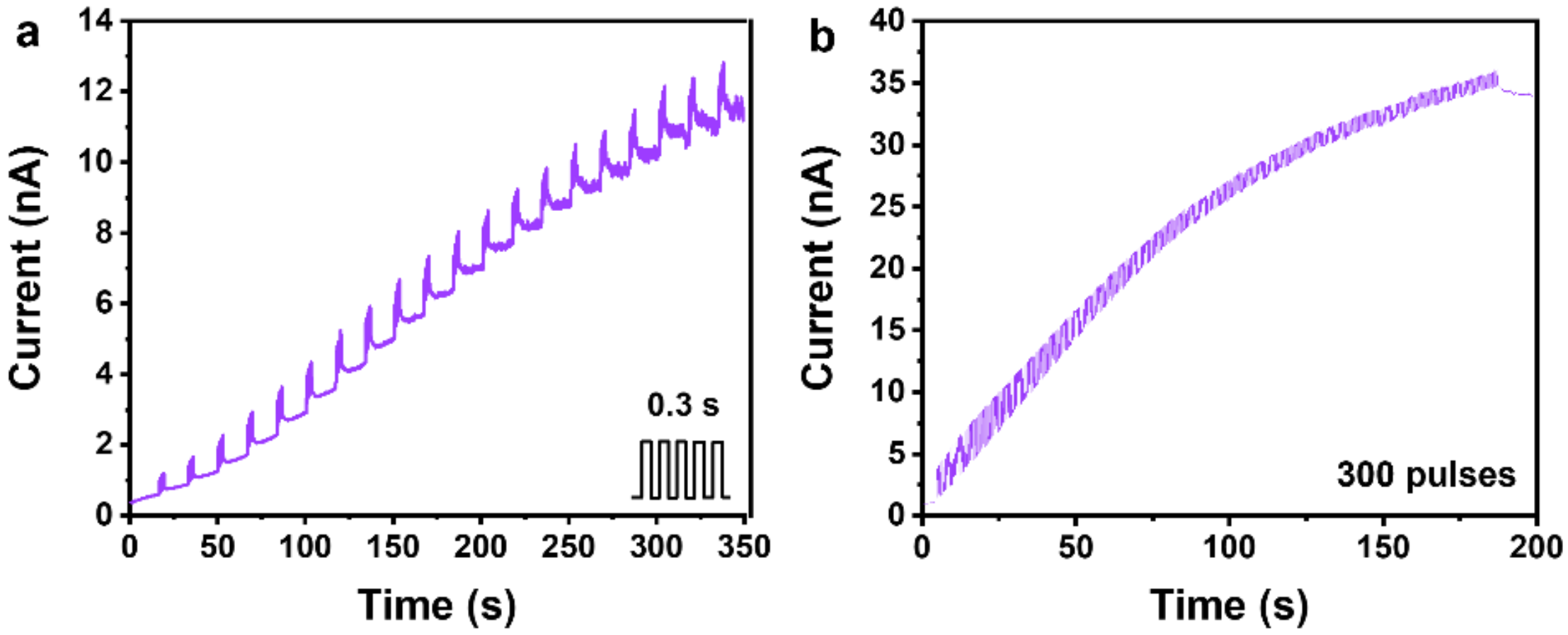

Supplementary Fig. 9 a Evolution of the photocurrent of ReS_2/BN/CIPS Fe-FET with time under 20 sets of optical stimuli, with each set consisting of five optical pulses (671 nm, 8.3 mW/cm^2, $\Delta t = 0.3$ s). **b** The evolution of the photocurrent with time under 300 optical pulses, demonstrating 300 distinguishable memory states.

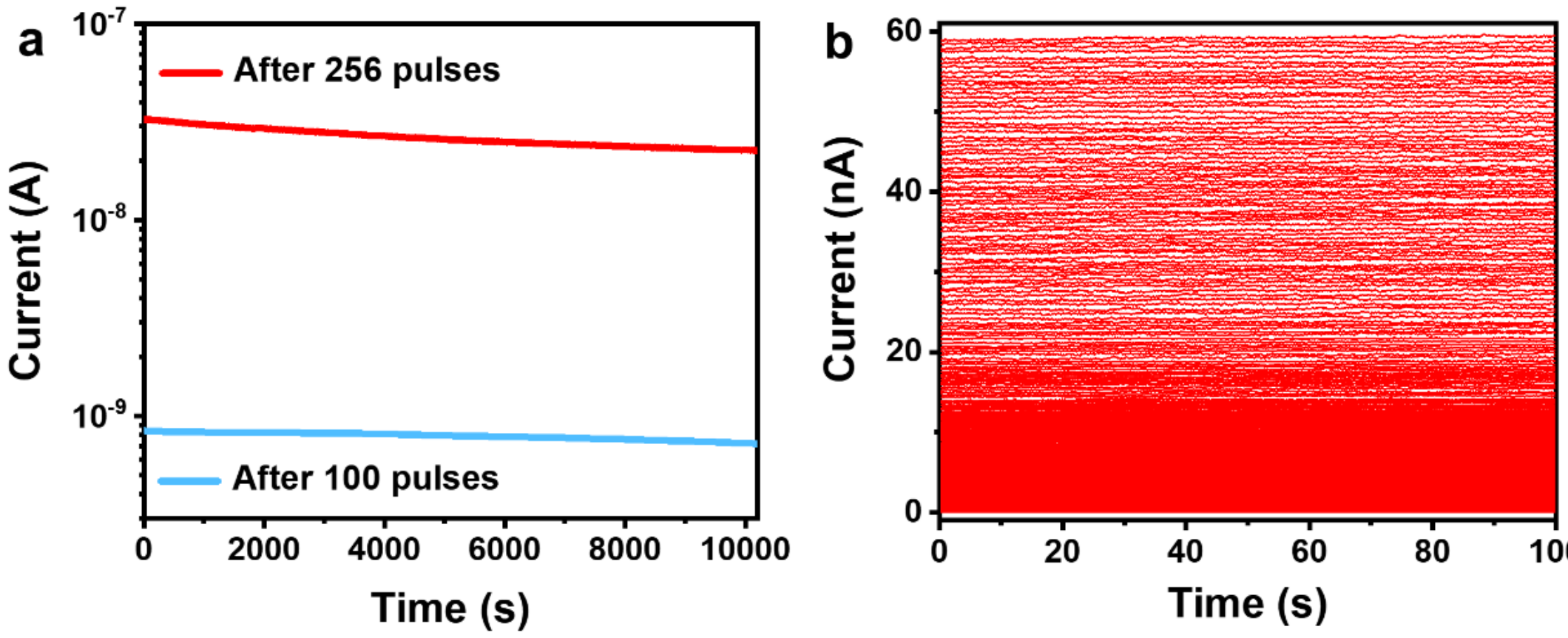

Supplementary Fig. 10 a Photocurrent retention of the device after 100 and 256 optical pulses (457 nm, 10.9 mW/cm^2). **b** Photocurrent retention under 300 consecutive optical pulses.

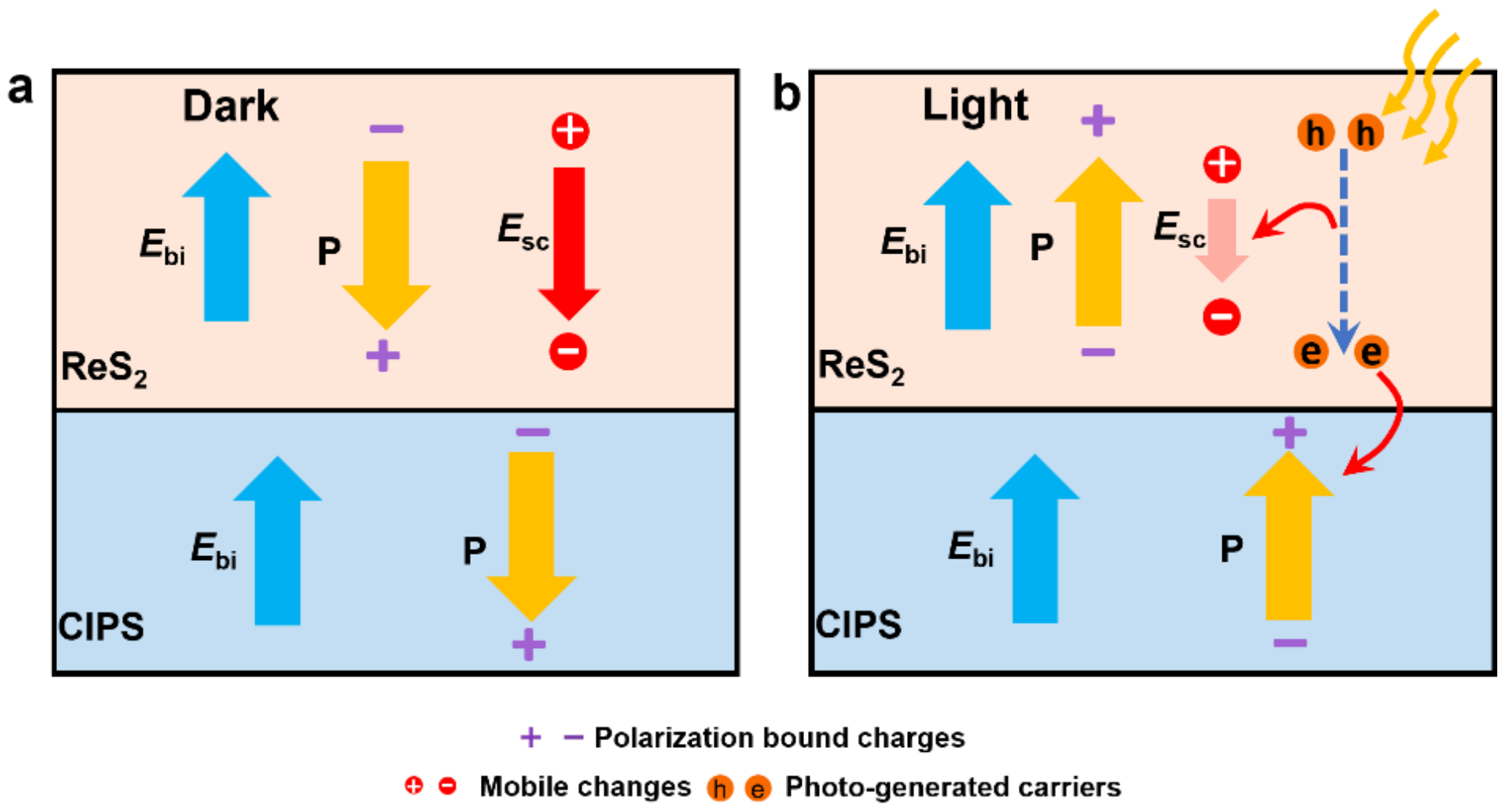

Supplementary Fig. 11 a, b Mechanism of light-induced polarization reversal.

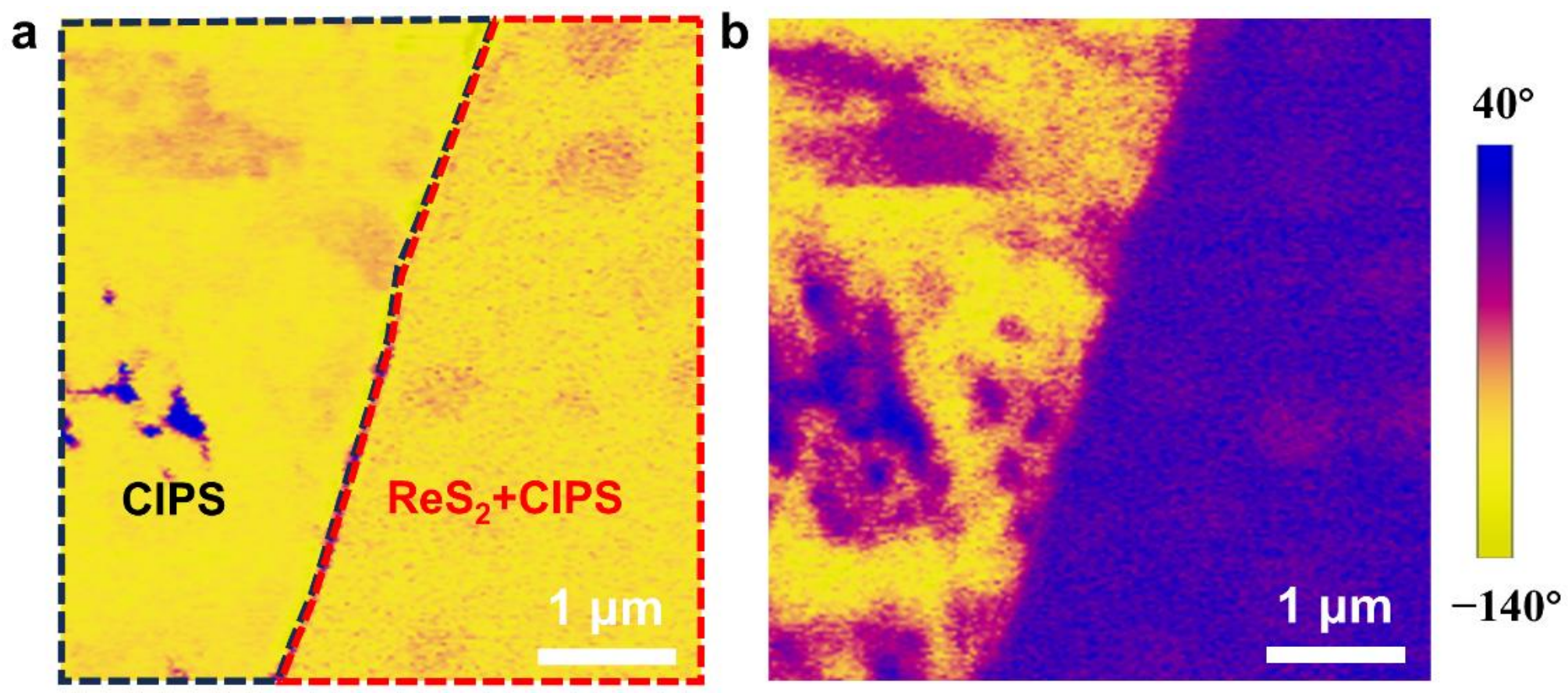

Supplementary Fig. 12 a, b PFM phase diagrams before and after light-induced ferroelectric domain evolution (457 nm, 10.9 mW/cm^2, 90 s). Scale bar: 1 μm.

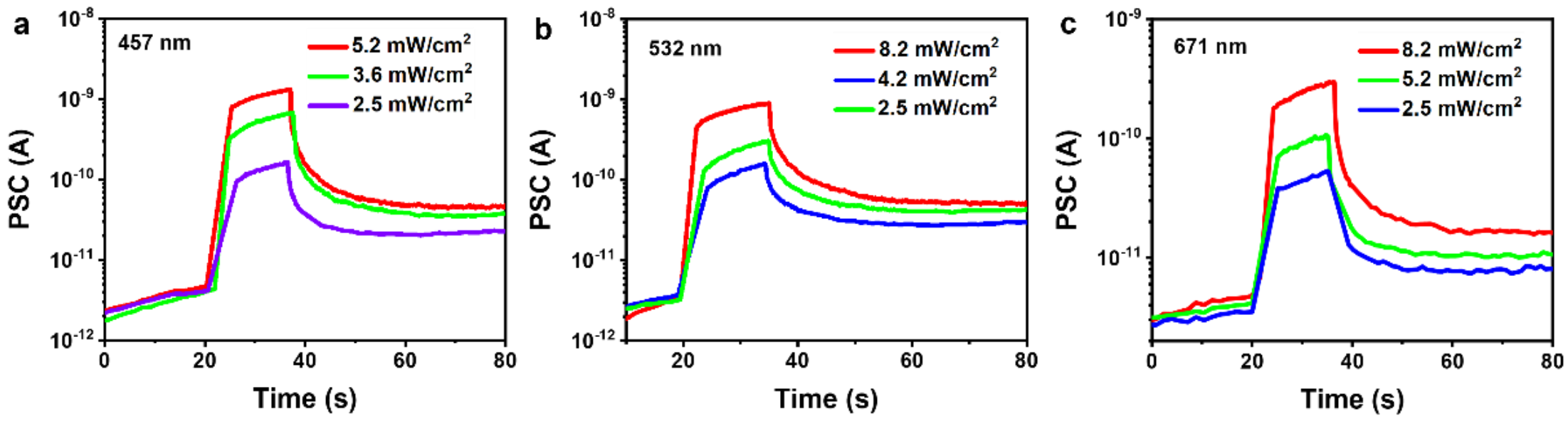

Supplementary Fig. 13 a-c The relationship between the postsynaptic current and time under different wavelengths and light intensities.

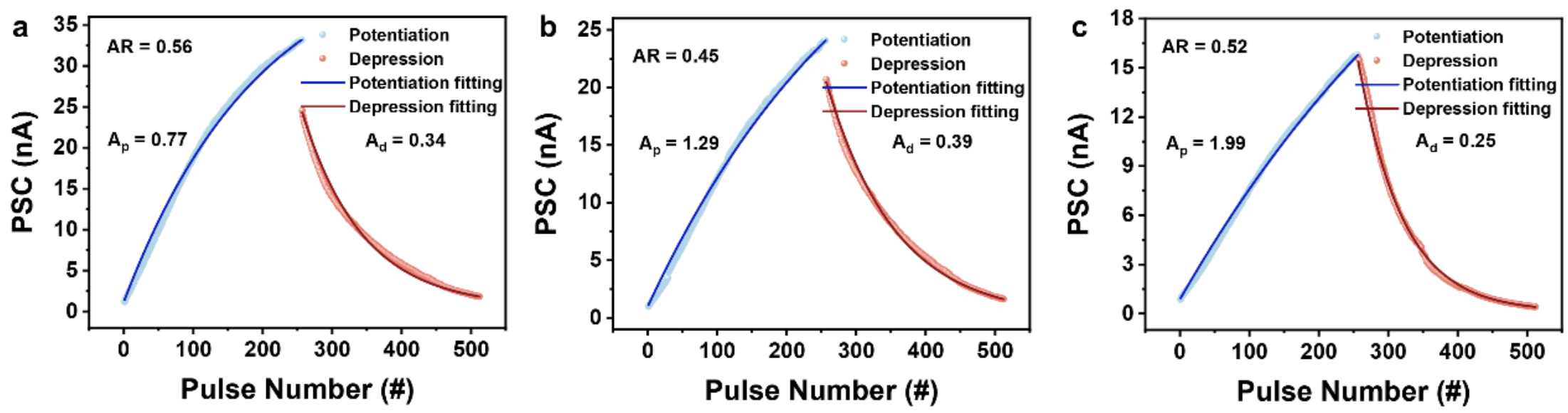

Supplementary Fig. 14 The Fitted curves for LTP/LTD characteristics and their linearity/symmetry.

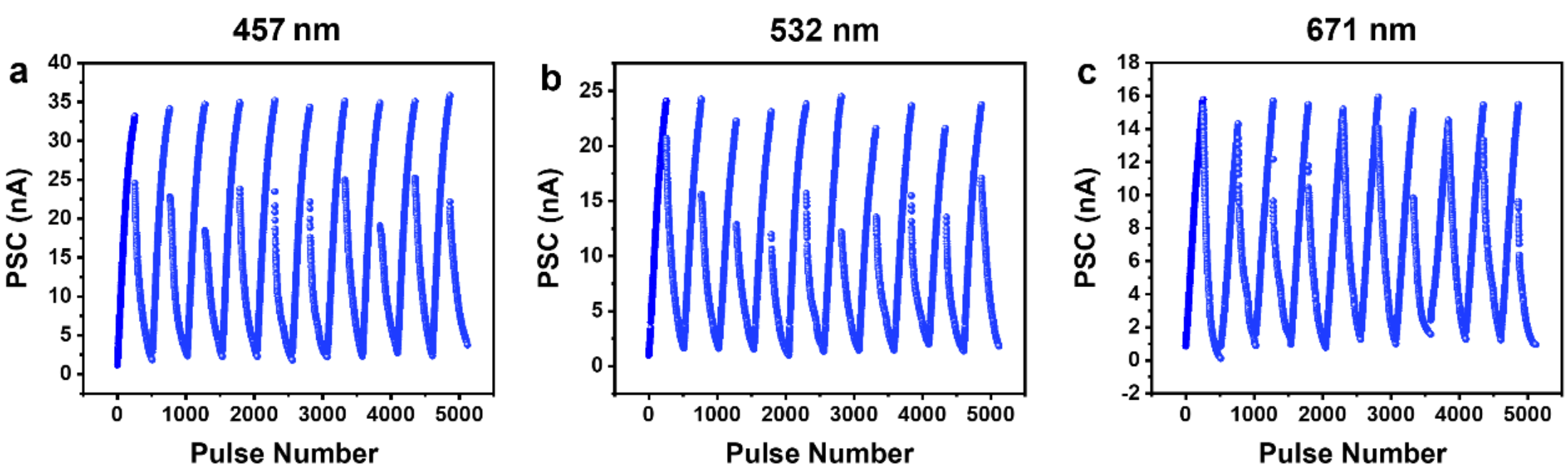

Supplementary Fig. 15 a-c Cyclic endurance of LTP/LTD synaptic behaviors under different wavelengths.

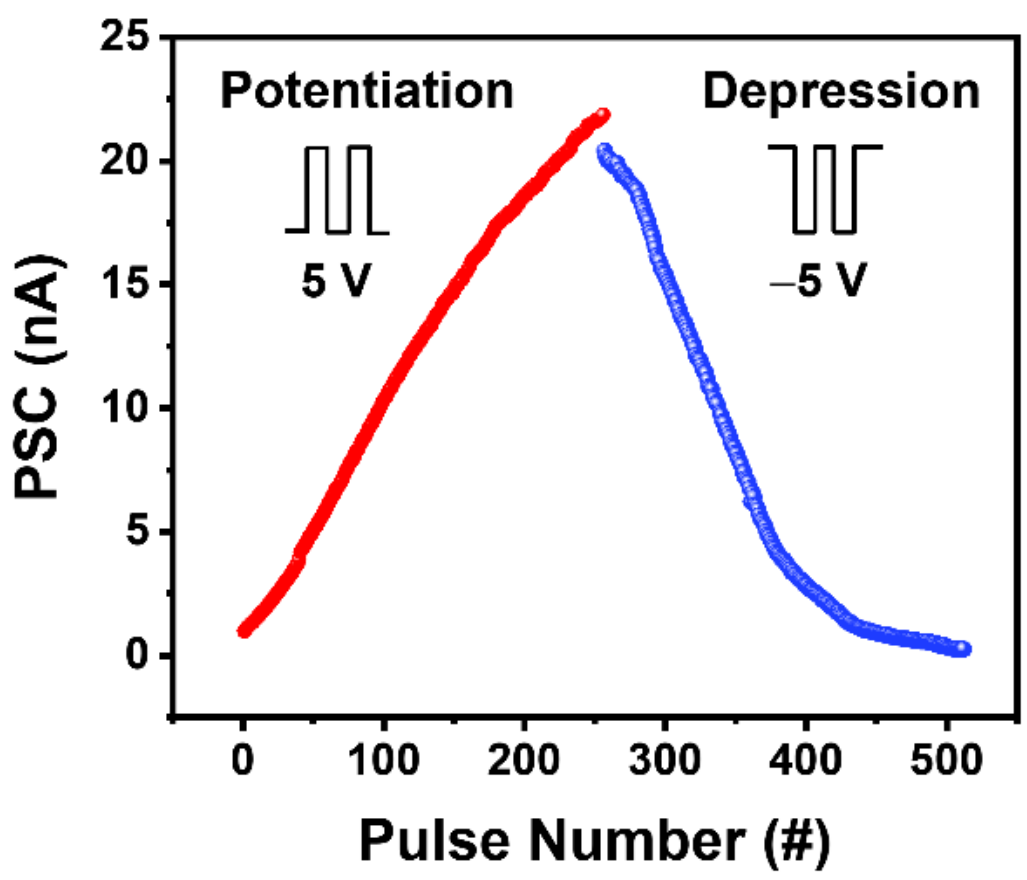

Supplementary Fig. 16 LTP and LTD behaviors of the device under ±5 V voltage stimulation.

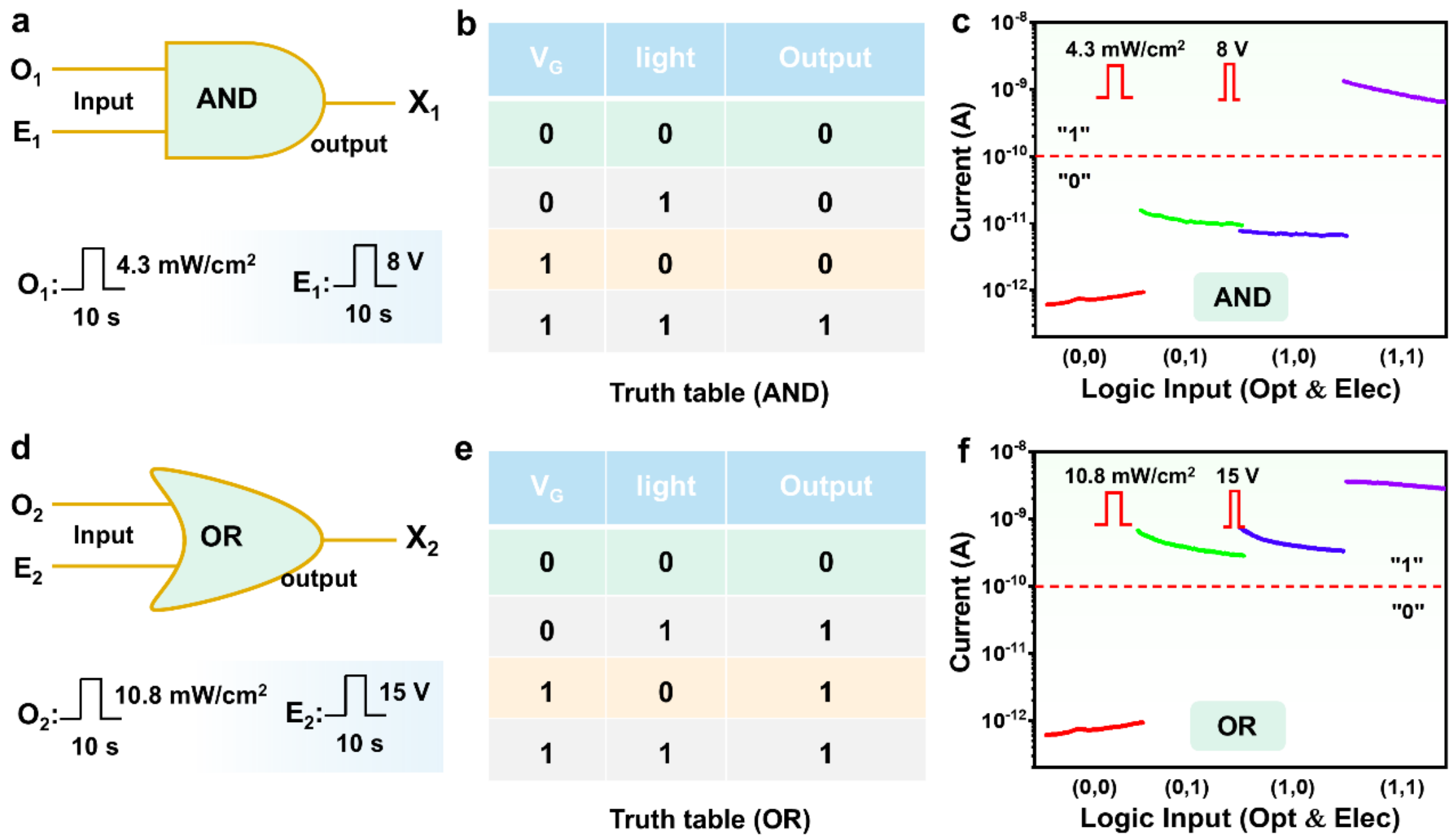

Supplementary Fig. 17 a, d Operation schemes for "AND" and "OR" logic functions and corresponding optical and electrical input signals. **b, e** Truth tables of the "AND" and "OR" logic functions. **c, f** Demonstration of the "AND" and "OR" logic-in-memory functions.

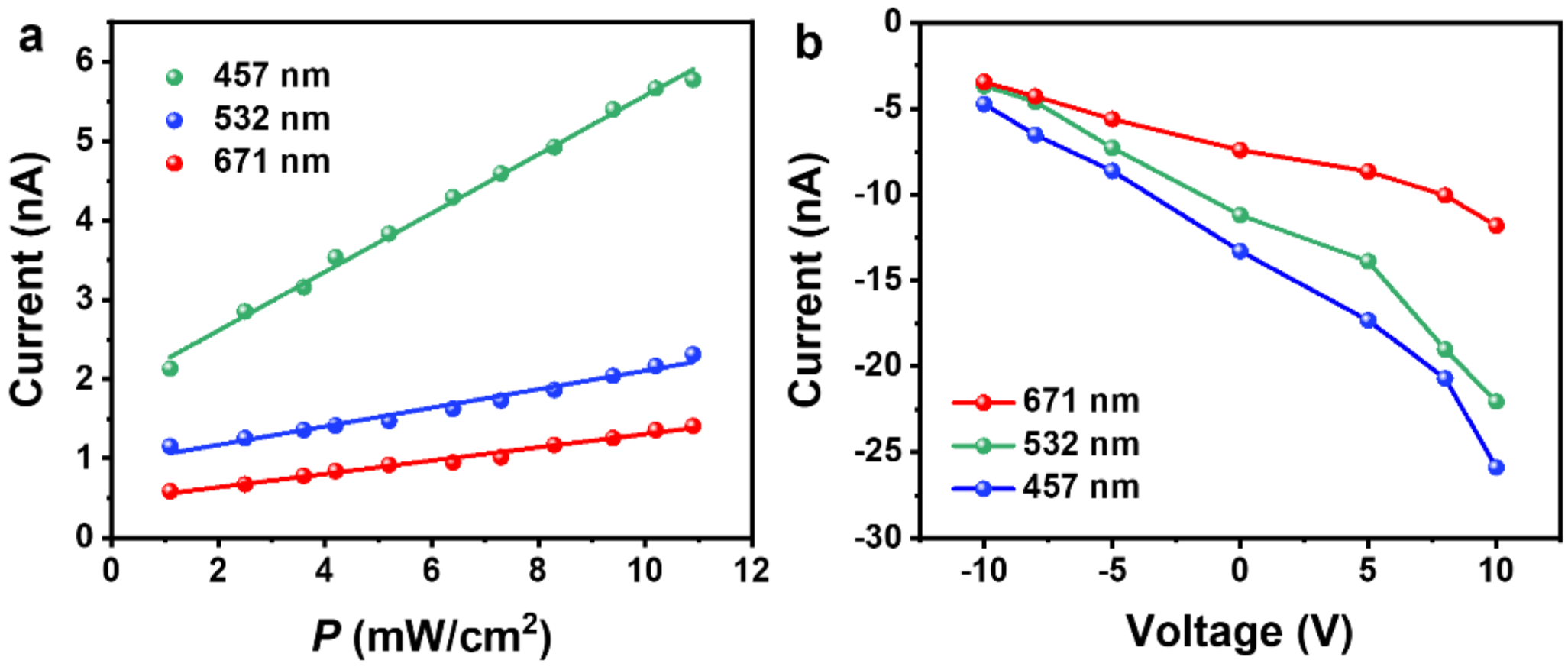

Supplementary Fig. 18 a The photocurrent response of the device as a function of light intensity at different wavelengths (457, 532, and 671 nm), V_{DS} = 0.5 V. **b** Polarization voltage-modulated photocurrent response of devices at different wavelengths.

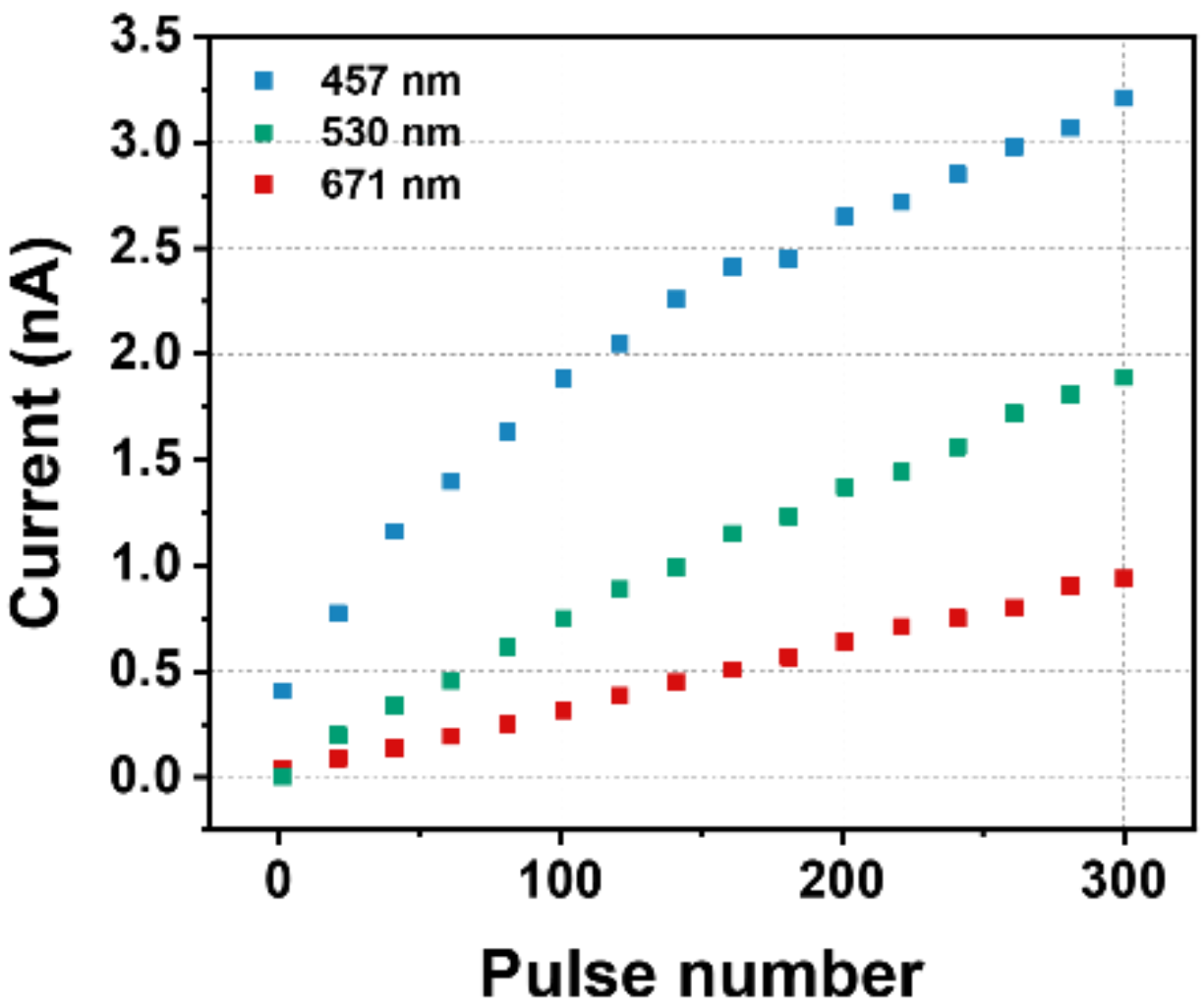

Supplementary Fig. 19 Current response versus optical pulse count across RGB wavelengths.

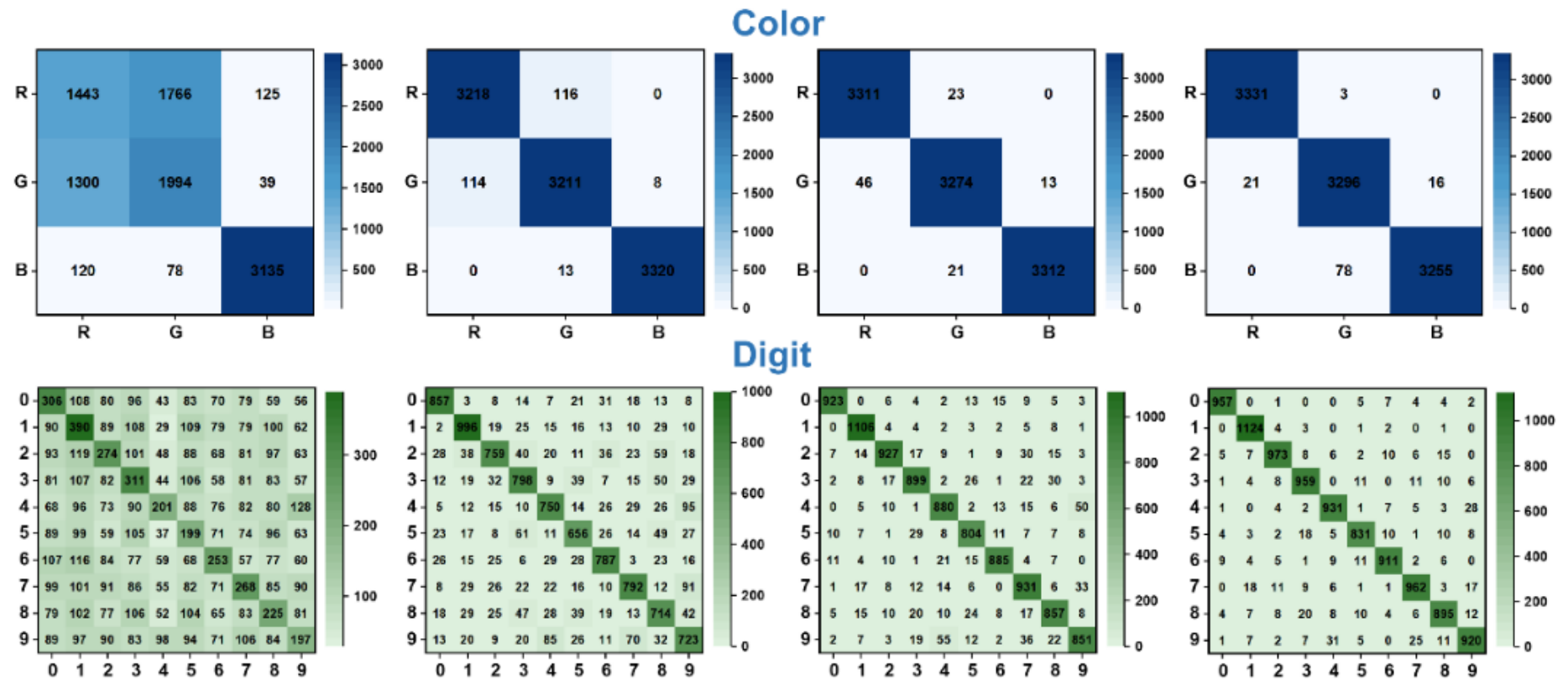

Supplementary Fig. 20 Confusion matrix for color and digit recognition results under 15% noise.

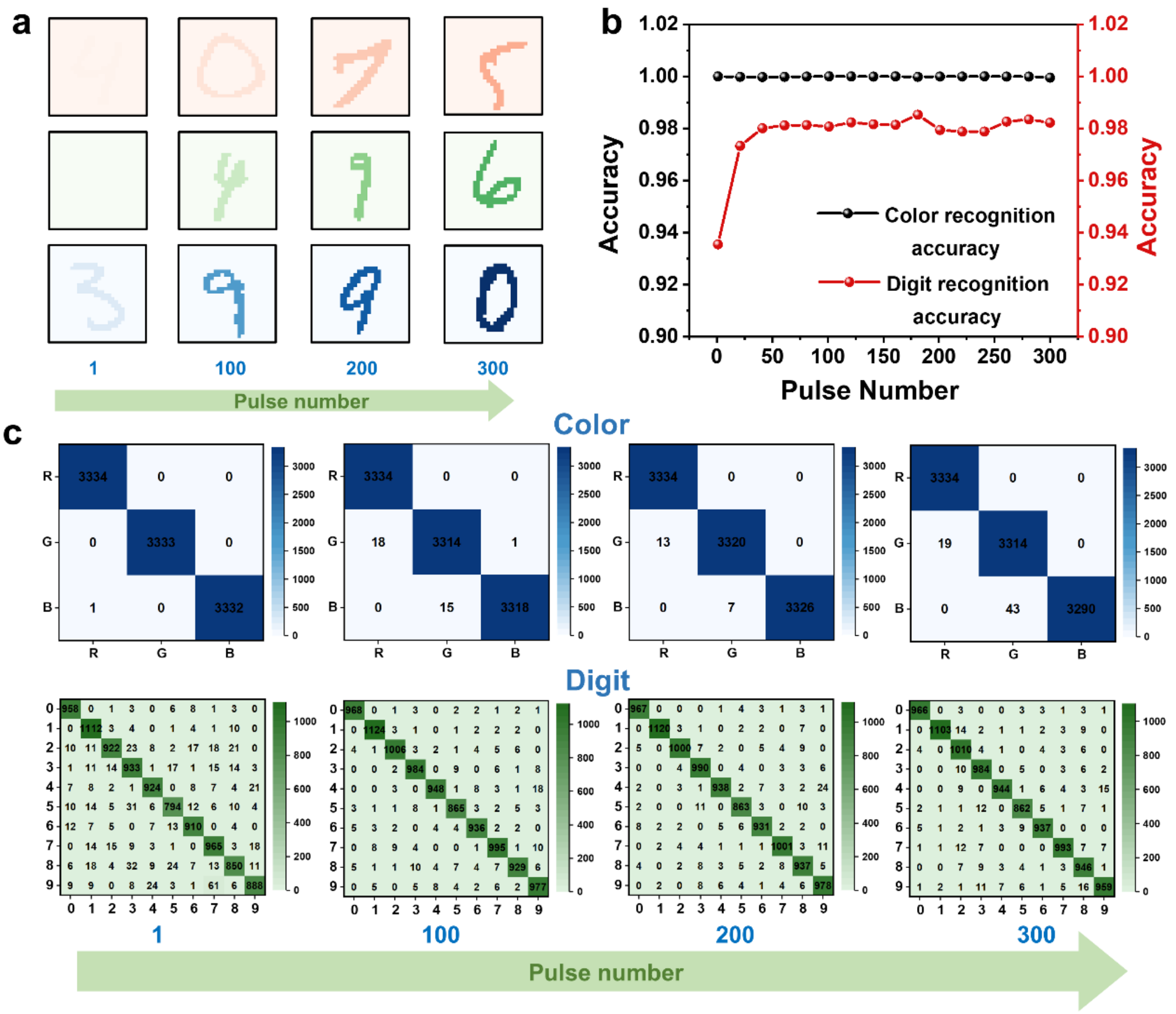

Supplementary Fig. 21 **a** Output photocurrent maps of the ferroelectric array in response to noisy colored digit inputs at different perception times (optical pulse numbers) under 0% noise. **b** The accuracy of digit and color recognition versus input pulse count. **c** Confusion matrix for color and digit recognition results across different pulse counts.

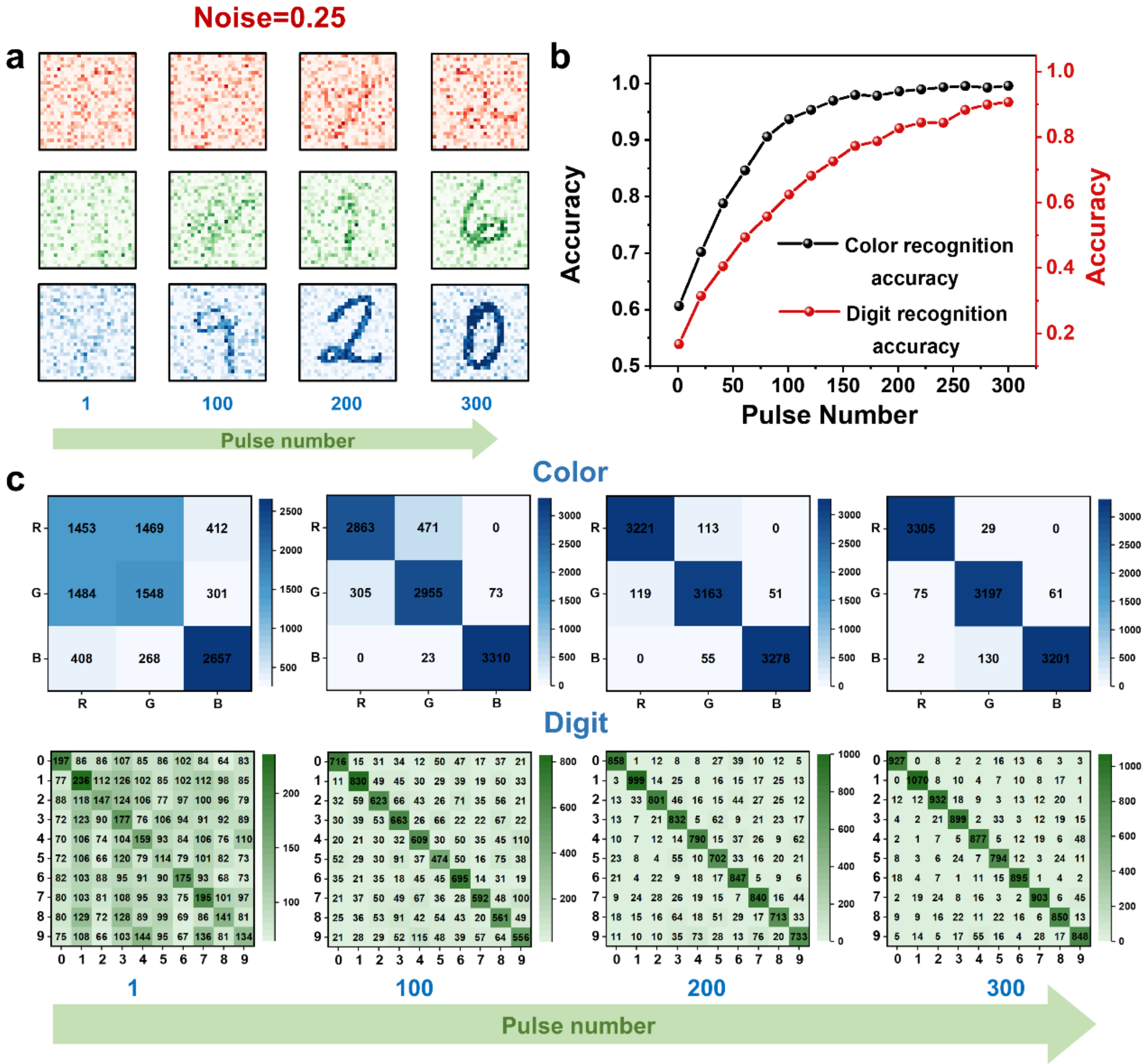

Supplementary Fig. 22 **a** Output photocurrent maps of the ferroelectric array in response to noisy colored digit inputs at different perception times (optical pulse numbers) under 25% noise. **b** Accuracy of digit and color recognition versus input pulse count. **c** Confusion matrix for color and digit recognition results across different pulse counts.

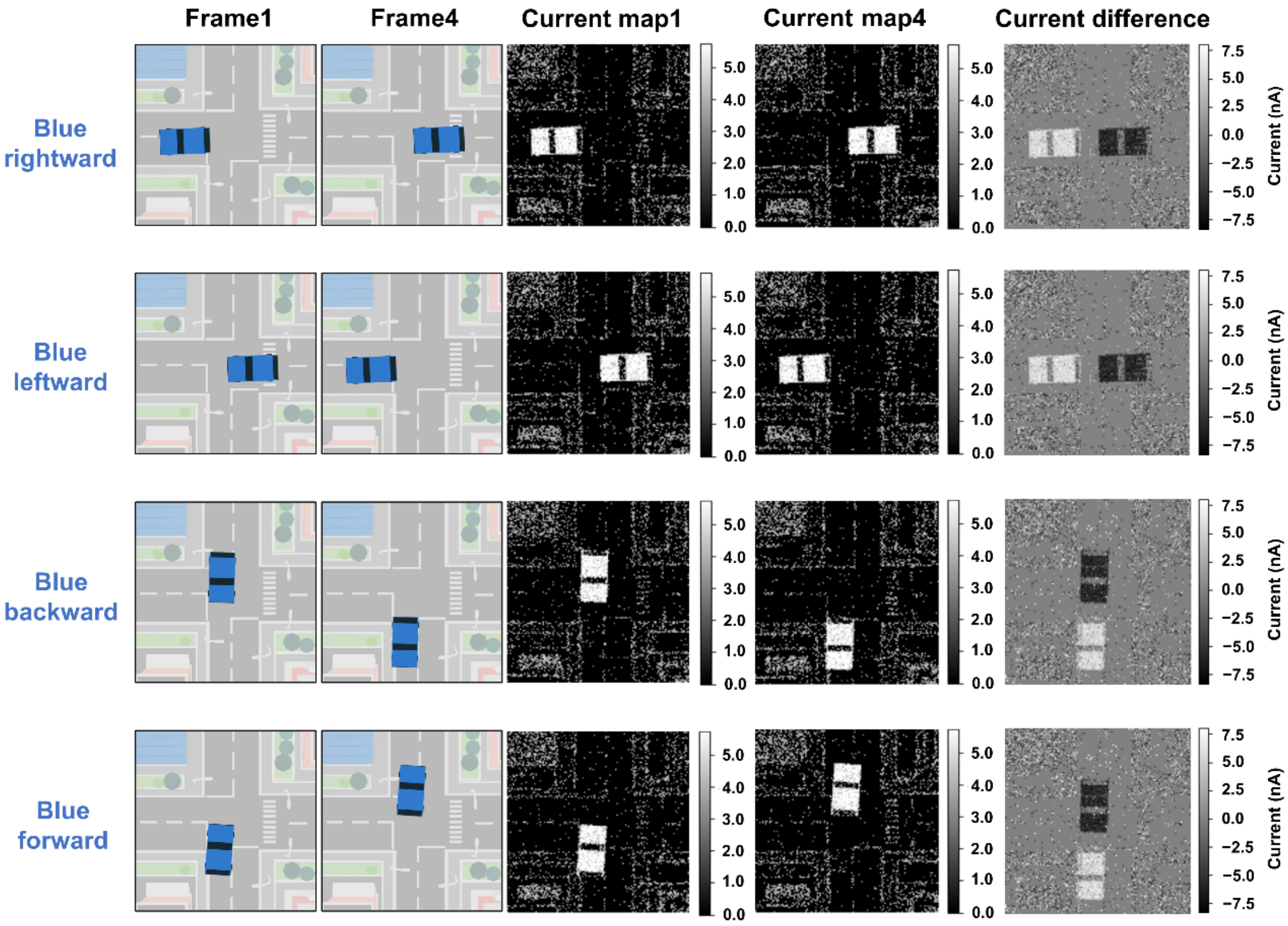

Supplementary Fig. 23 Illustration of four blue car motion scenarios. Rows 1-4 show a blue car moving rightward, leftward, backward, and forward, respectively. Each row contains five images: input frames 1 and 4, the corresponding output current maps from the array, and the inter-frame difference result.

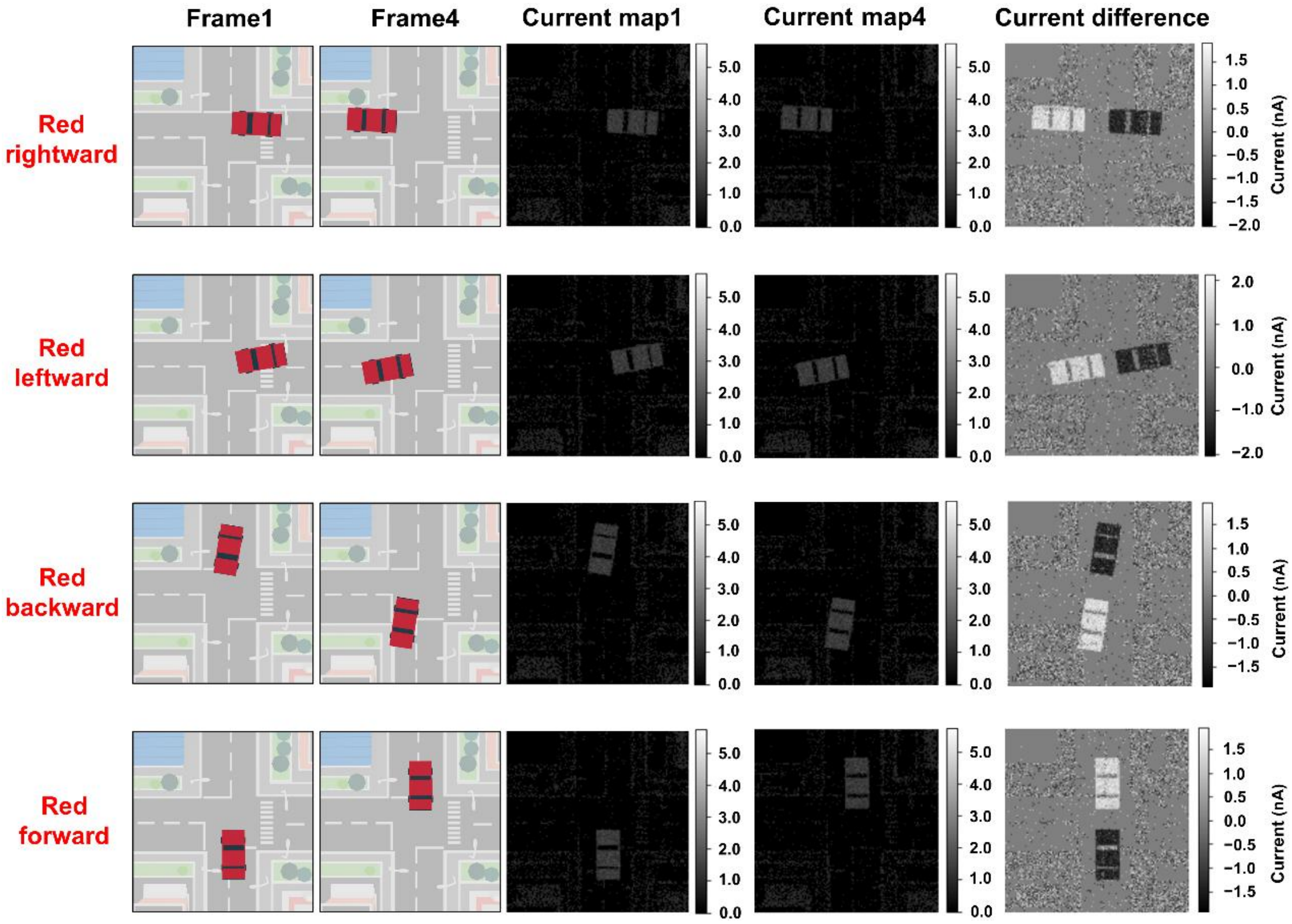

Supplementary Fig. 24 Illustration of four red car motion scenarios. Rows 1-4 show a red car moving rightward, leftward, backward, and forward, respectively. Each row contains five images: input frames 1 and 4, the corresponding output current maps from the array, and the inter-frame difference result.

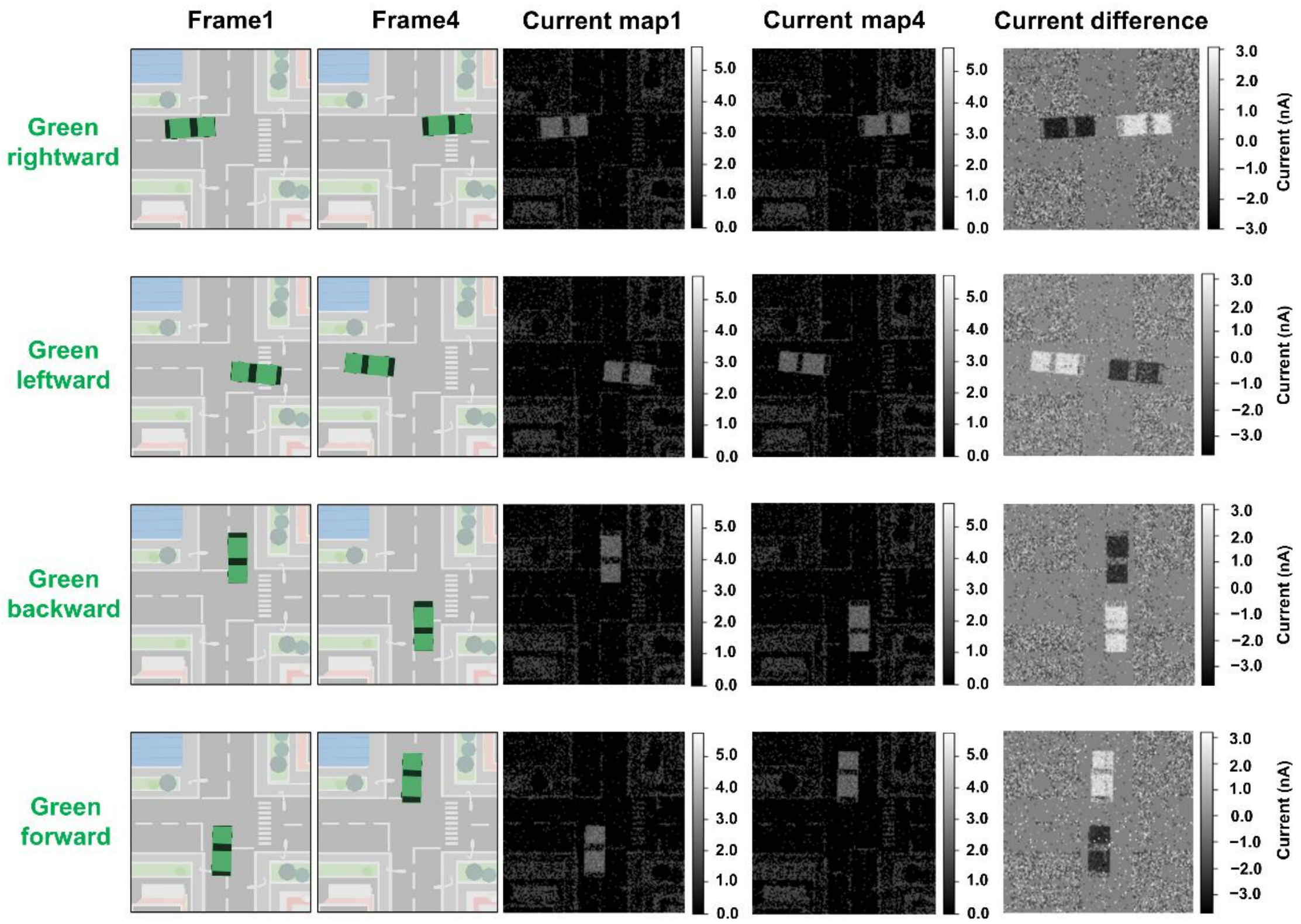

Supplementary Fig. 25 Illustration of four green car motion scenarios. Rows 1-4 show a green car moving rightward, leftward, backward, and forward, respectively. Each row contains five images: input frames 1 and 4, the corresponding output current maps from the array, and the inter-frame difference result.

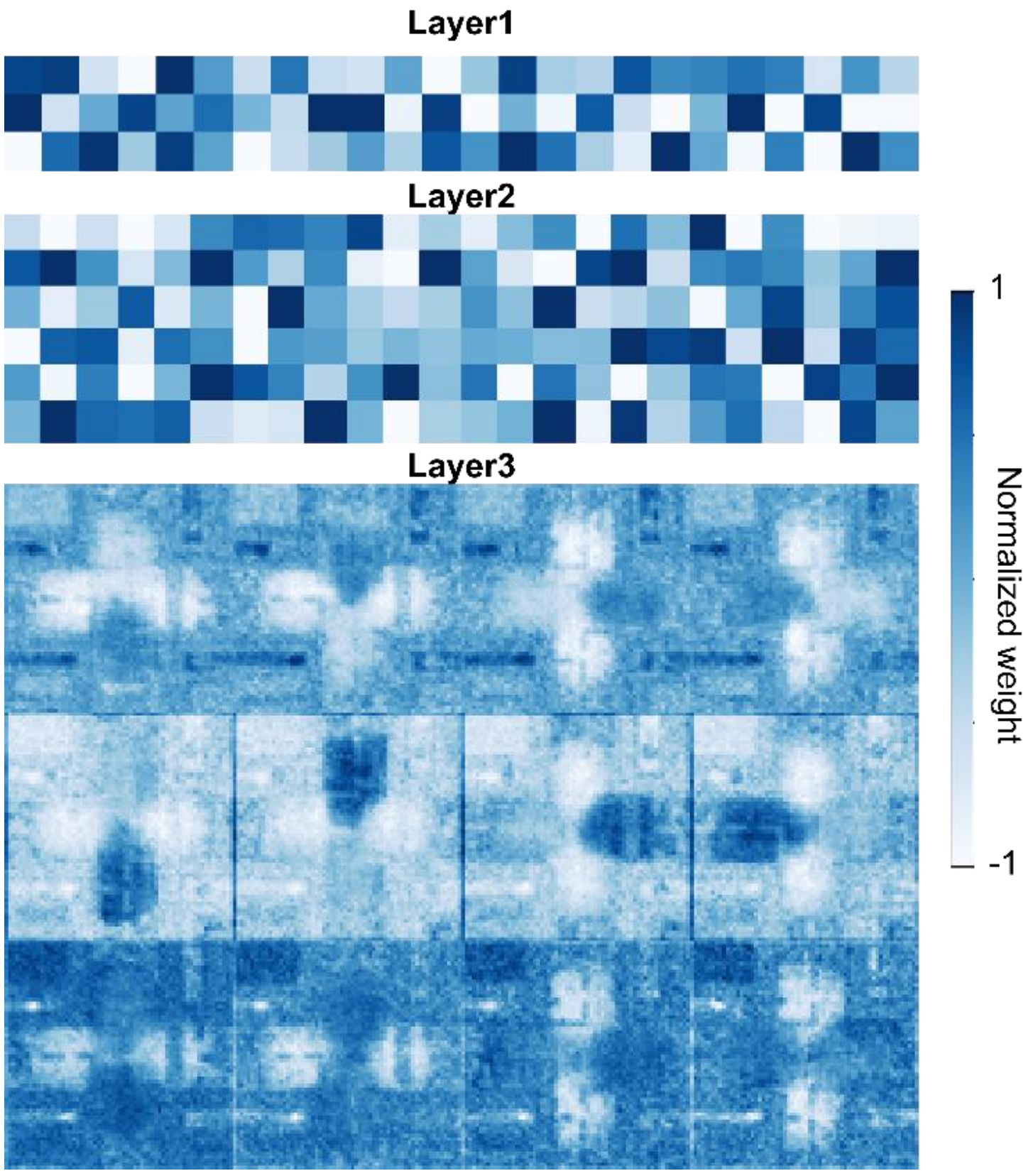

Supplementary Fig. 26 Weight map of the convolutional neural network for object color and motion direction after training.

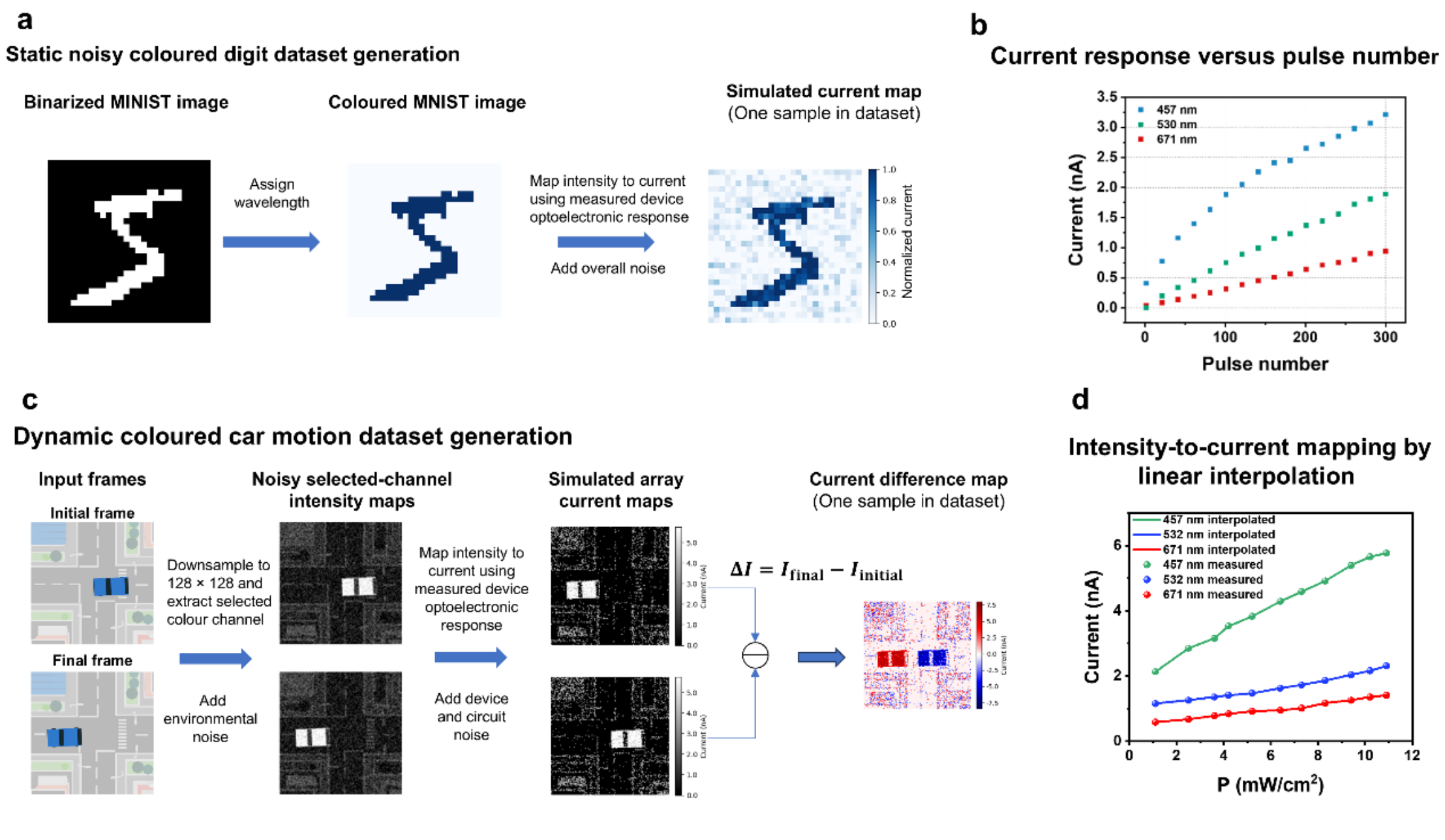

Supplementary Fig. 27 Workflow for generating simulated dataset samples in the

static and dynamic scenarios. a. Workflow for generating the noisy colored digit dataset. A binarized digit intensity map was first assigned to one color channel, followed by the addition of an overall Gaussian noise term. This overall noise term represents the combined noise level from possible environmental, device-related, and circuit-related contributions. The noisy intensity map was then mapped to a simulated array-output current map using the measured device current response versus optical pulse number at RGB-representative wavelengths. **b** Measured current response versus optical pulse number at wavelengths of 457, 532, and 671 nm. **c** Workflow for generating the simulated dynamic colored-car motion dataset. The initial and final frames were downsampled and converted into selected-channel intensity maps. Environmental Gaussian noise was then added to the extracted intensity maps, and the noisy intensity maps were then mapped to simulated array current maps using the measured wavelength- and intensity-dependent single-device response. Because the measured current-intensity response consists of discrete experimental points, linear interpolation was used to construct the wavelength-dependent intensity-to-current mapping function for converting all pixel intensities into simulated device currents. Device noise and circuit related noise were further added to the current maps before inter-frame differencing. The resulting current-difference map was used as one simulated array output sample. **d** Intensity-to-current mapping functions at 457, 532, and 671 nm. The measured discrete current–intensity data points were linearly interpolated to convert pixel intensities into simulated device currents.

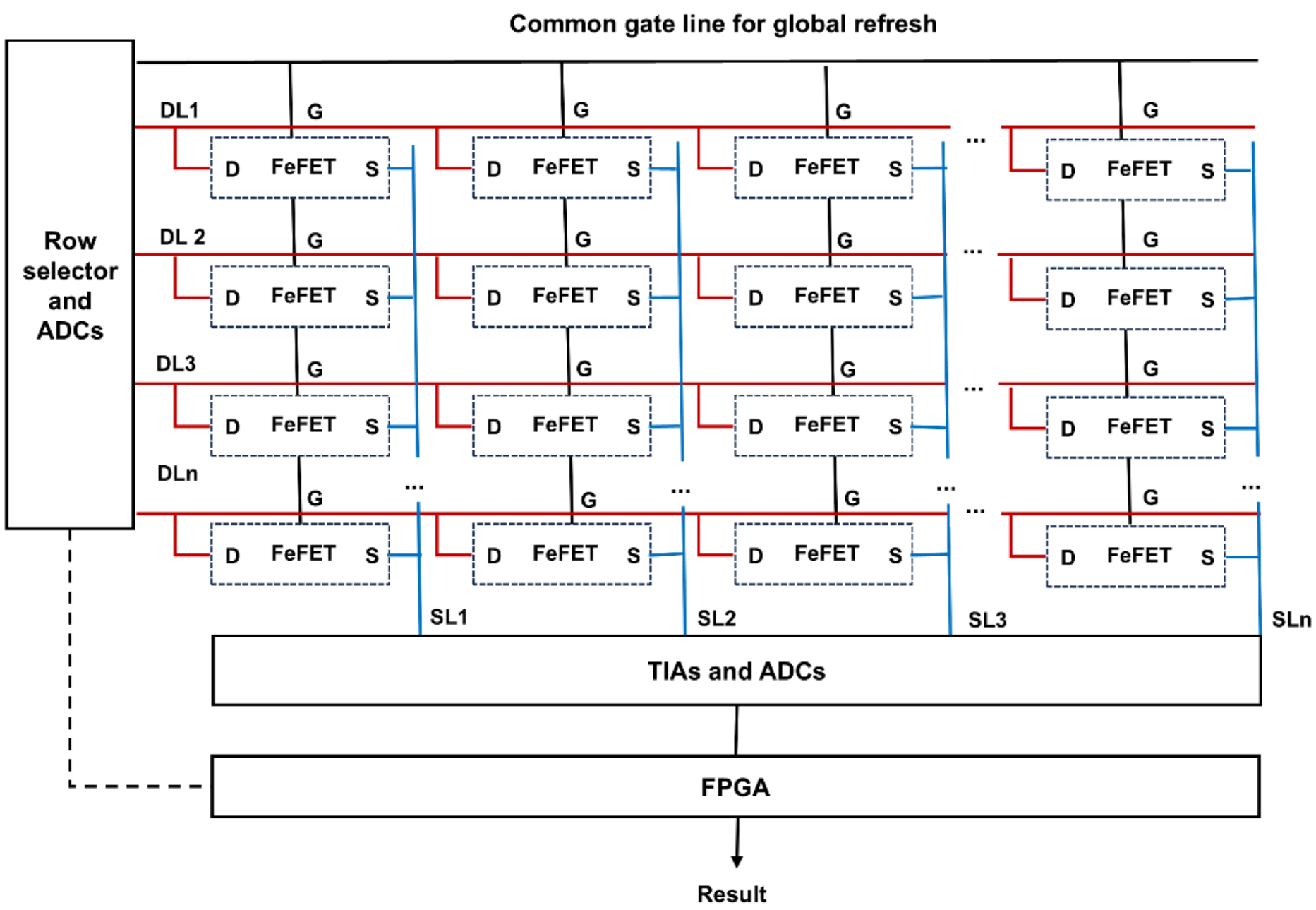

Supplementary Fig. 28 Proposed connection scheme of the FeFET array and peripheral circuits. Specific details are provided in Supplementary Note 4.

Supplementary Note 1. Power consumption

Compared to von Neumann computing architectures, low power consumption is one of the important advantages of artificial neuromorphic systems. Generally, the energy consumption of per synaptic event in biological brain is about 10 fJ, which can be calculated by the following equation: $E=I_{peak}\times V_{read}\times t$, where I_{peak}, V_{read}, and t are pulse current value, read voltage, and pulse width, respectively. Figure 3c demonstrates the postsynaptic currents of the synaptic device stimulated with light pulses of different intensities at a voltage of 0.01 V, and the minimum energy consumption per synaptic event is about 0.4 fJ (0.5 s). The power consumption is derived solely from the electrical energy consumption in the channel during the read operation, and it does not account for the optical input energy and polarization-programming energy.

Supplementary Note 2. Linearity and asymmetric for LTP/LTD characteristics.

The linearity of potentiation and depression is an important factor to evaluate the update of conductance, which is defined by the following equation:

$$G_p = B \cdot \left(1 - exp\left(-\frac{N}{A_p}\right)\right) + G_{min}, \quad (1)$$

$$\mathrm{G_d} = -\mathrm{B} \cdot \left(1 - \exp\left(-\frac{\mathrm{N\text{-}N_{max}}}{\mathrm{A_d}}\right)\right) + \mathrm{G_{max}}, \quad (2)$$

$$B = \frac{(G_{max} - G_{min})}{\left(1 - e^{-N_{max}/A_{p,d}}\right)} \quad (3)$$

where G_p and G_d are the conductance of potentiation and depression, respectively. A_p and A_d represent the linearity of potentiation and depression, respectively. N and N_{max} are the number and the maximum number of programming pulses, respectively. G_{max} and G_{min} are the maximum and minimum values of conductance.

The linear conductance change between them is evaluated by introducing the asymmetric ratio (AR), which is defined by the following equation:

$$AR = \frac{max|G_p(n) - G_d(n)|}{G_p(256) - G_d(256)}, (n = 1 \text{ to } 256) \quad (4)$$

where G_p (n) and G_d (n) are the channel conductance values after applying the nth light pulse and nth electrical spike, respectively.

Supplementary Note 3. Reconfigurable logic-in-memory functions

Non-volatile memory devices with logic functions are promising for improving the information processing capability of neuromorphic computing without static power consumption. The reconfigurable logic-in-memory functions with “AND” and “OR” are implemented based on ReS_2/BN/CIPS Fe-FET, where optical and electrical signals are defined as input signals for logic operations, and nonvolatile currents are utilized as output signals (Supplementary Fig. 12a, d). Supplementary Fig. 12b, e illustrates the

truth tables of the “AND” and “OR” logic gates. The “1” and “0” in the input signal refer to the “ON” and “OFF” states of the applied optical (electrical) input, and an output current value above 0.1 nA is defined as logic “1”, and otherwise is defined as logic “0”. It is important for choosing the optical light intensity and V_{GS} to realize logic-in-memory functions. When light pulse of 4.3 mW/cm^2 intensity (457 nm, Δt = 10 s) and electrical pulse of 8 V (Δt = 10 s) were used as input signals, the output current value was below the threshold when only the light or the electrical signal was input, which corresponds to logic “0”. When both light and electrical pulses were applied simultaneously, the output current was above the threshold, corresponding to logic “1”, which demonstrated the “AND” logic operation (Supplementary Fig. 12c). Furthermore, we demonstrated the “OR” logic operation by using a light pulse of 10.8 mW/cm^2 intensity (457 nm, Δt = 10 s) and an electrical pulse of 15 V (Δt = 10 s) as input signals (Supplementary Fig. 12f). It is worth noting that a negative gate voltage pulse (−15 V, Δt = 10 s) is employed to set the initial conductance state of the device before applying the input signal. The “AND” and “OR” logic operations are achieved in single device by synergistic effect between light and electrical stimuli, which can greatly improve the computing power, energy-efficiency, and computational accuracy of system to accomplish more complex tasks.

Supplementary Note 4. Array input and operation sequence

The FeFET front-end array is designed to sense and preprocess the optical input and store the preprocessed result in the proposed system-level implementation scheme. Each FeFET cell corresponds to one pixel. During optical input, the image is projected

onto the whole array in parallel. Owing to the wavelength-dependent photoresponse, intensity-dependent photoresponse, optical-pulse-dependent temporal accumulation, and optical memory behavior of the dual-FeFET, each cell senses and preprocesses the local optical information and stores the preprocessed result as a conductance state.

The array uses separated drain, source, and gate connections (Supplementary Fig. 28). The drain terminals are connected row by row as drain lines. During readout, a small read voltage is applied to one selected drain line, and the corresponding row of stored pixel states is read. The source terminals are connected column by column as source lines, and the output currents are converted by transimpedance amplifiers and digitized by analog-to-digital converters. The gate terminals are connected to a common gate line. After one frame is read out, a global gate pulse can be applied through this common gate line to refresh the array state before the next optical input. An FPGA coordinates the overall operation sequence.

In this operation, the optical stimulus is the raw optical input image. Its parameters include the wavelength or color and light intensity of each pixel. When temporal accumulation is used, the optical pulse number also serves as an input parameter. These optical parameters determine the sensing, preprocessing, and stored conductance state of each FeFET pixel. The electrical stimuli are used for array operation. The small drain read voltage is used to read the stored state from the selected row. The global gate voltage pulse is used to refresh the array state after the current frame has been read out. This refresh step reduces the influence of the stored information from the previous frame on the next optical input.

Supplementary Table 1. Comparison of the analog state numbers and memory on/off ratio of our device with those of previously reported optoelectronic synapse devices.

Device	LTP/LTD state	Memory on/off ratio	Reference
HfS_2	100	100	1
In_2Se_3/BN/CIPS	64	700	2
$MoTe_2$/P(VDF-TrFE)	52	10^3	3
SWNT@GDY nanotube	32	10^3	4
WSe_2	64	10^3	5
In_2Se_3	150	10^4	6
MoS_2/SiN_x	55	10^4	7
F8T2-SWCNT	40	10^4	8
MoS_2	20	10^5	9
In_2O_3	40	10^5	10
HfS_2/BN/Gr	50	10^5	11
WSe_2/Al_2O_3	100	10^5	12
MoS_2/BN/Gr	100	10^5	13
SnS_2/BN/CIPS	128	10^6	14
ReS_2/BN/CIPS	256	10^7	This work

Supplementary Table 2. Comparison of the power consumption and dark current of our device with those of previously reported optoelectronic synapse devices.

Device	Power consumption (J)	Dark current (A)	Reference
$Bi_3O_{2.5}Se_2$	5×10^{-16}	5×10^{-10}	15
InGaAs/P(VDF-TrFE)	8.4×10^{-16}	5×10^{-12}	16
MoS_2/BN/Gr	2.5×10^{-15}	1×10^{-12}	13
WS_2/HZO	5×10^{-15}	1×10^{-12}	17
In_2Se_3	4×10^{-14}	8×10^{-13}	18
HfS_2/BN/Gr	2×10^{-13}	5×10^{-12}	11
BP/P(VDF-TrFE)	4×10^{-14}	1.5×10^{-7}	19
SnS_2/BN/Gr	1.6×10^{-12}	1×10^{-12}	20
WSe_2/P(VDF-TrFE)	4×10^{-12}	1×10^{-11}	21
MoS_2/2D-RPP	5.1×10^{-12}	2.5×10^{-9}	22
MoS_2/BN/2D-RPP	1×10^{-11}	1×10^{-9}	23
In_2Se_3/BN/CIPS	6.9×10^{-12}	3×10^{-12}	2
C8-BTBT/PVA/P(VDFTrFE)	3.4×10^{-11}	1×10^{-12}	24
Pentacene/P(VDF-TrFE)	3.8×10^{-8}	5×10^{-11}	25
ReS_2/BN/CIPS	4×10^{-16}	6×10^{-15}	This work

Supplementary References

1. Xiong, H. et al. Optically modulated HfS_2-based synapses for artificial vision systems. *ACS Appl. Mater. Interfaces* **13,** 50132-50140 (2021).
2. Das, B. et al. Artificial visual systems fabricated with ferroelectric van der waals heterostructure for in-memory computing applications. *ACS Nano* **17,** 21297-21306 (2023).
3. Wu, G. et al. Ferroelectric-defined reconfigurable homojunctions for in-memory sensing and computing. *Nat. Mater.* **22,** 1499-1506 (2023).
4. Zhang, G.-X. et al. Broadband sensory networks with locally stored responsivities for neuromorphic machine vision. *Science* **9,** eadi5104 (2023).
5. Wu, R. et al. Biomimetic artificial tetrachromatic photoreceptors based on fully light-controlled 2D transistors. *Adv. Funct. Mater.* **33,** 2305677 (2023).
6. Chen, Y. et al. Bidirectional synaptic phototransistor based on two-dimensional ferroelectric semiconductor for mixed color pattern recognition. *ACS Nano* **17,** 12499 (2023).
7. Tan, F. et al. Multifunctional broadband artificial visual system using all-in-one two-dimensional optoelectronic transistors. *Mater. Today* **81,** 23-35 (2024).
8. Sui, N. et al. Photoprogrammed multifunctional optoelectronic synaptic transistor arrays based on photosensitive polymer-sorted semiconducting single-walled carbon nanotubes for image recognition. *Adv. Sci.* **11,** 2401794 (2024).
9. Liu, L. et al. Two-dimensional MoS_2-based anisotropic synaptic transistor for neuromorphic computing by localized electron beam irradiation. *Adv. Sci.* **11,**

2408210 (2024).

10. Jin, C. et al. Bidirectional photovoltage-driven oxide transistors for neuromorphic visual sensors. *Adv. Mater.* **37,** 2410398 (2025).

11. Xu, L. et al. High conductance margin for efficient neuromorphic computing enabled by stacking nonvolatile van der waals transistors. *Phys. Rev. Appl.* **16,** 044049 (2021).

12. Gong, Y. et al. Bioinspired artificial visual system based on 2D WSe_2 synapse array. *Adv. Funct. Mater.* **33,** 2303539 (2023).

13. Sun, Y. et al. Programmable van-der-waals heterostructure-enabled optoelectronic synaptic floating-gate transistors with ultra-low energy consumption. *InfoMat* **4,** e12317 (2022).

14. Wang, P. et al. Integrated in-memory sensor and computing of artificial vision based on full-vdw optoelectronic ferroelectric field-effect transistor. *Adv. Sci.* **11,** e2305679 (2024).

15. He, Y. et al. The discovery of a high-mobility two-dimensional bismuth oxyselenide semiconductor and its application in nonvolatile neuromorphic devices. *ACS Nano* **17,** 10783-10791 (2023).

16. Xie, P. et al. Ferroelectric P(VDF-TrFE) wrapped ingaas nanowires for ultralow-power artificial synapses. *Nano Energy* **91,** 106654 (2022).

17. Chen, L. et al. A van der waals synaptic transistor based on ferroelectric $Hf_{0.5}Zr_{0.5}O_2$ and 2D tungsten disulfide. *Adv. Electron. Mater.* **6,** 2000057 (2020).

18. Wang, S. et al. Two-dimensional ferroelectric channel transistors integrating ultra-

fast memory and neural computing. *Nat. Commun.* **12,** 53 (2021).

19. Dang, Z. et al. Black phosphorus/ferroelectric P(VDF-TrFE) field-effect transistors with high mobility for energy-efficient artificial synapse in high-accuracy neuromorphic computing. *Nano Lett.* **23,** 6752-6759 (2023).
20. Gao, F. et al. Low optical writing energy multibit optoelectronic memory based on SnS_2/h-BN/graphene heterostructure. *Small* **17,** e2104459 (2021).
21. Zhou, Y. et al. A reconfigurable Two-WSe_2-transistor synaptic cell for reinforcement learning. *Adv. Mater.* **34,** 2107754 (2022).
22. Lai, H. et al. Fast, multi-bit, and vis-infrared broadband nonvolatile optoelectronic memory with MoS_2/2D-perovskite van der waals heterojunction. *Adv. Mater.* **35,** 2208664 (2023).
23. Lai, H. et al. Photoinduced multi-bit nonvolatile memory based on a van der waals heterostructure with a 2D-perovskite floating gate. *Adv. Mater.* **34,** 2110278 (2022).
24. Li, L. et al. Autonomous light intensity adaptation in an energy-efficient retinomorphic organic ferroelectric neuristor. *Adv. Opt. Mater.* **12,** 2303172 (2024).
25. Jang, S. et al. Ultrathin conformable organic artificial synapse for wearable intelligent device applications. *ACS Appl. Mater. Interfaces* **11,** 1071-1080 (2018).
