# Supplementary material for: Dual-ferroelectric stacked high-performance field-effect transistor
Source: Nat Commun. 2026 Aug 19;17:9905. doi: 10.1038/s41467-026-76907-4 (PMC13582962; doi:10.1038/s41467-026-76907-4)
Supplement: Supplementary file 2 [file 41467_2026_76907_MOESM2_ESM.pdf]

nature portfolio

Peer Review File

Dual-ferroelectric stacked high-performance field-effect transistor

Corresponding Author: Professor Xiao-Hong Xu

This file contains all reviewer reports in order by version, followed by all author rebuttals in order by version.

Version 0:

Reviewer comments:

Reviewer #1

(Remarks to the Author)
Wang et al. propose a dual-ferroelectric field-effect transistor (dual-FeFET) that leverages ferroelectric polarization reversal induced by two stimuli (optical and electrical) and demonstrate its potential in multitask scenarios. While the reported single-device metrics (On/off ratio, dark current, and >8-bit operation) are indeed impressive, the manuscript does not yet provide sufficient validation of reliability and reproducibility. In addition, the distinction between experimentally demonstrated results and those obtained from simulated/virtual arrays is not clearly articulated. In its current form, the paper would require substantial revision prior to publication.

1. The manuscript presents non-volatile memory operation as a key aspect of the dual-FeFET functionality. However, the claim that the retention and endurance are "excellent" appears overstated. The reported retention is measured up to 1.6 × 10^4 s, and the endurance is on the order of 1200 cycles.

2. Rather than only reporting the existence of >300 states, it is more important to demonstrate whether these states remain separable over time. Retention and drift characteristics for the multilevel states should be provided. Without such analysis, the claim of the number of "distinguishable" states may be inflated.

3. The manuscript uses strong language such as "constructed artificial visual system" and "implemented real-time … system," yet also describes a "simulated 28 × 28 dual-FeFET array." It should be stated unambiguously (particularly in the Abstract and Results) whether a physical 28 × 28 array was fabricated and measured, or whether the array-level results are obtained from simulations parameterized by single-device measurements.

4. Related to point 3, if the system-level demonstrations are simulation-based, the Methods should describe (in a fully reproducible manner) how single-device characteristics (wavelength-dependent response) were mapped onto pixels/cells. It should also be clarified whether D2D variability, read noise, and other non-idealities were included, and how the measured/assumed device responses were incorporated during training and testing.

5. The reported energy consumption (~0.4 fJ) is not sufficiently defined. The authors should explicitly state whether the optical input energy is included or excluded. If the estimate accounts only for electrical dissipation, it should be described precisely (for example, "electrical energy consumed in the channel during read/write").

6. The manuscript should more convincingly disentangle optically induced ferroelectric polarization reversal from contributions associated with trapping/photogating. Additional experiments would strengthen this claim, such as (if feasible) comparing PFM domain configurations or polarization hysteresis before/after illumination, and/or employing pulsed electrical measurements designed to minimize trap-related artifacts in transfer characteristics.

7. The "related manuscript" file cannot be opened due to an error, preventing evaluation of the associated content. This needs to be fixed for proper review.

Minor comment.
1. In Fig. 2(a), the legend is missing. In addition, the voltage axes should be labeled using standard FET notation (e.g., V_GS, V_DS), since the plotted voltages are not absolute values but bias conditions defined with respect to the relevant

terminals.

Reviewer #2

(Remarks to the Author)
The work presents an opto-electrical ferroelectric memory with demonstrated reconfigurability with potential for applications in visual systems. The authors perform experimental characterization of the devices to demonstrate the functionalities that are essential for neural-network operations, such as LTP, LTD, Retention, etc.
While the overall outlook is interesting, there are few critical details that are vague and unclear.

The following points require further clarification.

1. Retention Time:
- One key selling point of the proposed memory is the ability to change the conductance state both optically and electrically.
- The authors demonstrate sufficiently long retention time, but only for electrical operations.
- Authors should demonstrate the long retention time for optical write operations, and at multiple memory states that are achieved through LTP using optical write operations.

2. Endurance:
- Same as above, that the reported endurance is for electrical write operations, while the key selling point of the device is opto-electric behavior. Therefore, authors should demonstrate endurance for optical operations as well.

3. LTP and LTD:
- The authors demonstrate multi-level capabilities with the LTP and LTD and demonstrate linearity. However, only LTP is performed optically while the LTD is performed electrically.
- Further, authors show polarization reversal only for Pdown to Pup (Line 156), and not the other way around.
- Therefore, authors should clarify if the polarization reverse is possible for program and erase operations.
- Further, authors should explain the implication of this 'limitation' on the overall application. That is, it is likely that a given device may receive optical stimuli while simultaneously receiving an electrical stimulus during write operations. Does this affect anything, as the LTP is optical and LTD is electrical.

4. Logic Operations:
Authors demonstrate logical AND and OR operations. Authors should provide:
- How the logic gates are constructed
- The context in which the AND and OR operations fit in their end application.
- One input is optical while another is electrical. Why not keep both to be either optical or electrical?
- The magnitude of optical and electrical stimuli strength is different between AND and OR operations. Authors should specify why?
- In the end processing, what would be the source of electrical and optical stimuli? Which physical parameter (e.g. pixel for pattern recognition?) does it correspond to?

5. Energy Calculations:
- Authors should provide the power consumption calculations and specify/provide a breakdown of each component.
- Reason being, author require pulses to operate the proposed device, and the peripherals that drive such large voltages can be energy consuming. Clarifying this w/ a breakdown of energy consumption will give a better overall picture.

6. Operation of Neural Network:
- Array Connection: In which configuration the FeFET array is connected? What are the inputs to the array? How is it connected to the peripherals?
- What is the input both parameter wise and stimuli wise? What is the role of electrical stimuli, optical stimuli?
- Is the network pre-trained with the data-set, or the optical-electrical parameters will be used to train it as well?

Reviewer #3

(Remarks to the Author)
The authors propose a dual-ferroelectric stacked field-effect transistor that successfully achieves reconfigurable integration of functions including non-volatile memory, synaptic simulation, and mixed-color image processing. The device not only demonstrates outstanding performance in current on/off ratio, multi-level optoelectronic storage, and number of analog states, but also exhibits multi-task parallel recognition capability and high recognition accuracy in handling both static and dynamic mixed-color visual information. However, some issues need to be addressed before publication, as follows.
1. This dual-ferroelectric field-effect transistor demonstrates outstanding performance in non-volatile memory and synaptic plasticity. However, the authors only compared this work with existing studies in terms of current on/off ratio, dark current, number of analog states, and power consumption. How does the high PPF index reported in this manuscript compare to that of recently reported advanced optoelectronic synapse devices?
2. The manuscript achieves multi-task parallel processing of static/dynamic mixed-color visual information. Could the authors clarify whether other recent studies also possess similar bio-inspired visual parallel processing capabilities? If so, please provide a comparison in terms of recognition accuracy or functional integration. If not, it is recommended to further highlight the innovation of this work.
3. The performance of the device is highly dependent on its fabrication process. It is recommended that the authors provide a

detailed flowchart to illustrate the key fabrication steps.
4. In neural network simulations, the linearity and symmetry of weight updates (conductance modulation) directly impact the network's learning efficiency and recognition accuracy. Please quantitatively evaluate the symmetry and linearity of LTP and LTD in Figures 5d-f.
5. The CNN architecture diagram shown in Figure 5b does not effectively illustrate the core concept of parallel recognition of color and motion direction.

Reviewer #4

(Remarks to the Author)
I co-reviewed this manuscript with one of the reviewers who provided the listed reports. This is part of the Nature Communications initiative to facilitate training in peer review and to provide appropriate recognition for Early Career Researchers who co-review manuscripts.

Version 1:

Reviewer comments:

Reviewer #1

(Remarks to the Author)
The authors have addressed most of the technical concerns raised in the previous round, particularly regarding device reliability, multilevel-state retention, the distinction between measured single-device data and simulation-based array demonstrations, and the mechanism of light-induced ferroelectric polarization reversal. I still have some reservations about the overall novelty and contribution of the manuscript, since the array-level demonstrations remain simulation-based rather than experimentally implemented. However, I leave this broader editorial judgment to the editor. From a technical standpoint, the revision is substantially improved, and only the following minor points remain.

1. The energy-consumption claim should be further qualified. The reported value of ~0.4 fJ refers only to the electrical energy consumed in the channel during read operation and excludes the optical input energy as well as the initial polarization-programming energy. This limitation should be stated consistently in the main text, figure caption, and any summary statements, so that the value is not interpreted as the total device- or system-level energy consumption.

2. The added photo-assisted PFM data provide useful support for light-induced ferroelectric polarization reversal. However, these results do not completely exclude possible contributions from trapping or photogating effects, especially under the actual pulsed optical conditions used for synaptic operation. The authors should therefore avoid overly strong mechanistic language and describe ferroelectric polarization reversal as the dominant or primary contribution, rather than the sole mechanism.

Reviewer #3

(Remarks to the Author)
All revisions have been completed. Recommended for publication.

Reviewer #4

(Remarks to the Author)
I co-reviewed this manuscript with one of the reviewers who provided the listed reports. This is part of the Nature Communications initiative to facilitate training in peer review and to provide appropriate recognition for Early Career Researchers who co-review manuscripts.

Version 2:

Reviewer comments:

Reviewer #1

(Remarks to the Author)
The authors have adequately addressed the remaining comments and concerns.

Reviewer #4

(Remarks to the Author)
I co-reviewed this manuscript with one of the reviewers who provided the listed reports. This is part of the Nature Communications initiative to facilitate training in peer review and to provide appropriate recognition for Early Career Researchers who co-review manuscripts.

Open Access This Peer Review File is licensed under a Creative Commons Attribution 4.0 International License, which permits use, sharing, adaptation, distribution and reproduction in any medium or format, as long as you give appropriate credit to the original author(s) and the source, provide a link to the Creative Commons license, and indicate if changes were made.
In cases where reviewers are anonymous, credit should be given to 'Anonymous Referee' and the source.
The images or other third party material in this Peer Review File are included in the article's Creative Commons license, unless indicated otherwise in a credit line to the material. If material is not included in the article's Creative Commons license and your intended use is not permitted by statutory regulation or exceeds the permitted use, you will need to obtain permission directly from the copyright holder.
To view a copy of this license, visit https://creativecommons.org/licenses/by/4.0/

List of Responses and Revision

Title: "Dual-ferroelectric stacked high-performance field-effect transistor"

ALL THE CHANGES WERE DONE IN RED IN THE REVISED MANUSCRIPT.

REVIEWER COMMENTS

Reviewer #1 (Remarks to the Author):

Wang et al. propose a dual-ferroelectric field-effect transistor (dual-FeFET) that leverages ferroelectric polarization reversal induced by two stimuli (optical and electrical) and demonstrate its potential in multitask scenarios. While the reported single-device metrics (On/off ratio, dark current, and >8-bit operation) are indeed impressive, the manuscript does not yet provide sufficient validation of reliability and reproducibility. In addition, the distinction between experimentally demonstrated results and those obtained from simulated/virtual arrays is not clearly articulated. In its current form, the paper would require substantial revision prior to publication.

Response: We thank the reviewer for the comments on our work. We have carefully considered all comments/suggestions of the reviewer. According to the reviewer's valuable suggestions, the manuscript was revised accordingly as follows.

1. The manuscript presents non-volatile memory operation as a key aspect of the dual-FeFET functionality. However, the claim that the retention and endurance are "excellent" appears overstated. The reported retention is measured up to 1.6 × 10^4 s, and the endurance is on the order of 1200 cycles.

Response: Thank you for the valuable comment. We fully agree with the reviewer that using the term "excellent" based on the existing data is somewhat overstated. Regarding retention characteristics, the initial data indicate that no significant decay was observed over 1.6×10^4 s, which demonstrates superior performance compared to similar devices [Nature Communications, 12, 53 (2021); Nature Communications, 14, 7891 (2023); Nature Communications, 16, 9382 (2025)]. Additionally, we have retested the cycle endurance of the device. As shown in **Fig. R1**, the current ratio exhibits no significant degradation over 6000 cycles, demonstrating good cyclic stability [Nature Communications, 14, 7891 (2023); Advanced Materials, 36, 2403785 (2024)]. To avoid exaggeration, we have revised the term "excellent" to "good" in the revised manuscript **(Pages 4, 6)**, and have updated the endurance data in **Supplementary Fig. 7** of the revised supplementary information.

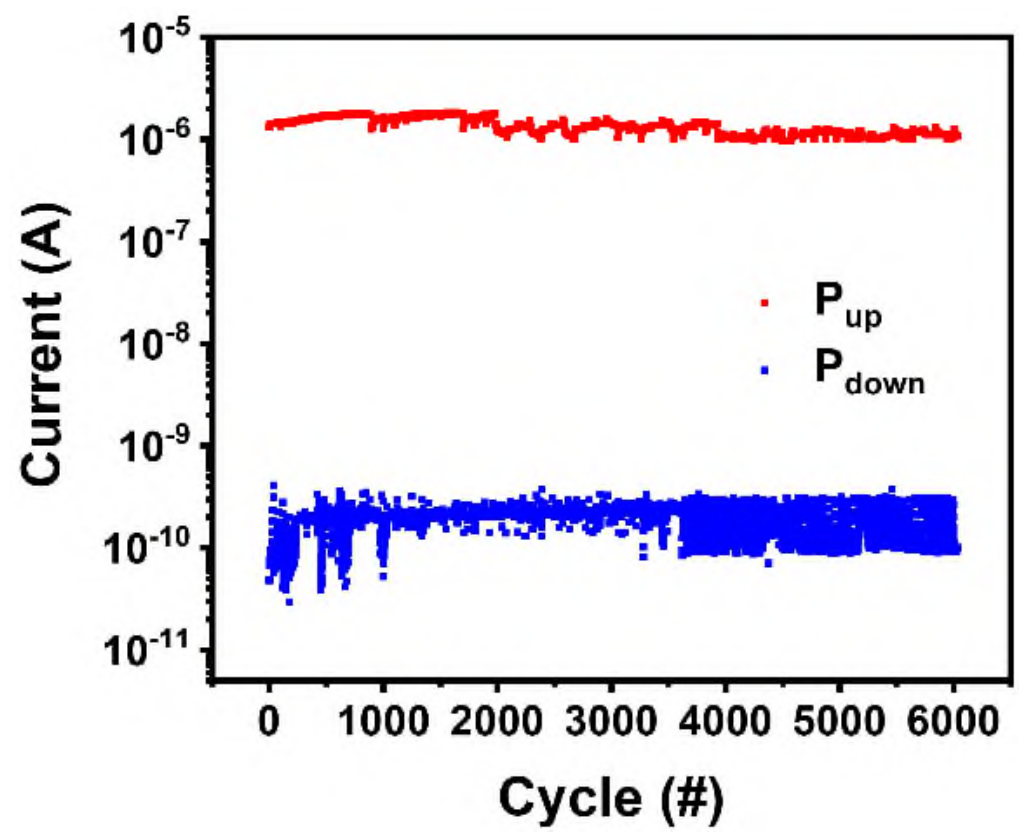

Fig. R1 Endurance performance of ReS_2/BN/CIPS Fe-FET after over 6000 erase/write cycles.

2. Rather than only reporting the existence of >300 states, it is more important to demonstrate whether these states remain separable over time. Retention and drift characteristics for the multilevel states should be provided. Without such analysis, the claim of the number of "distinguishable" states may be inflated.

Response: Thank you for the valuable comment. According to the reviewer's comments, we supplemented the retention and drift characteristics of the multi-level memory states by applying 300 consecutive light pulses. As shown in **Fig. R2**, the 300 conductance states maintain good distinguishability, and no significant overlap was observed between adjacent states, demonstrating that the device possesses stable and distinguishable multilevel memory characteristics. The relevant results and descriptions were added to the revised manuscript and supplementary information **(Supplementary Fig. 10 and Page 7)**.

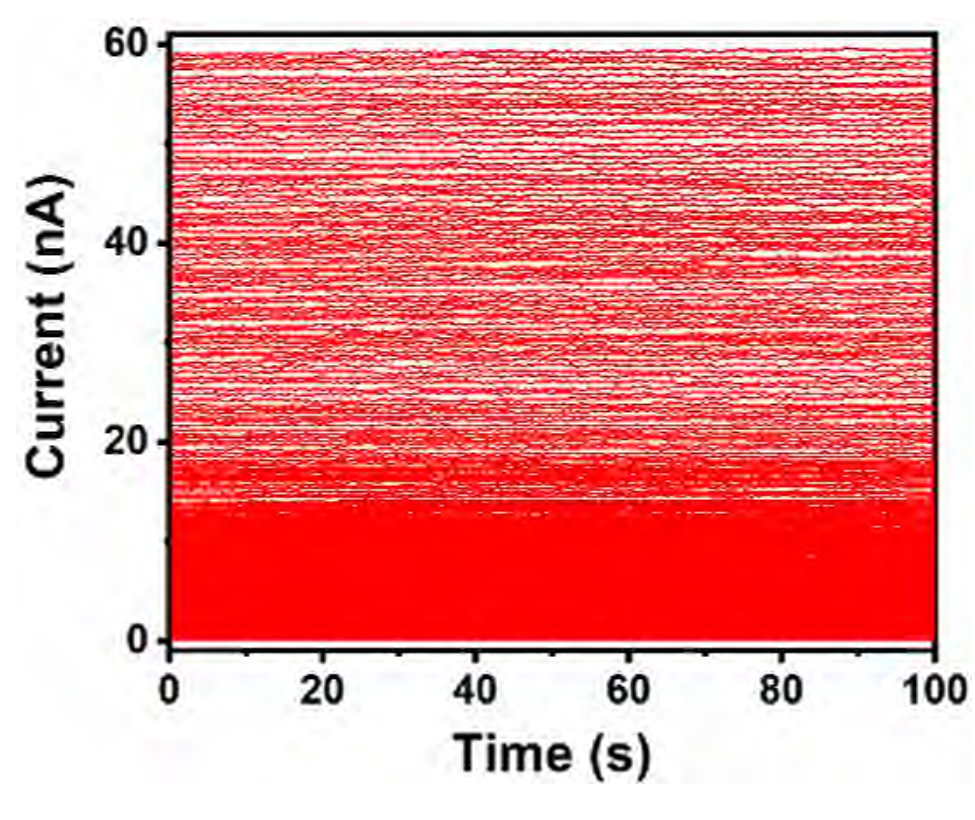

Fig. R2 Photocurrent retention under 300 consecutive optical pulses.

3. The manuscript uses strong language such as "constructed artificial visual system" and "implemented real-time … system," yet also describes a "simulated 28 × 28 dual-FeFET array." It should be stated unambiguously (particularly in the Abstract and Results) whether a physical 28 × 28 array was fabricated and measured, or whether the array-level results are obtained from simulations parameterized by single-device measurements.

Response: Thank you for the valuable reminder and comment. The relevant

descriptions in the manuscript may lead to ambiguity. It should be clarified that our work did not fabricate or measure a physical 28×28 array, the array-level results were obtained from simulations parameterized by single-device measurements. To avoid misunderstanding, we have revised the relevant descriptions in the revised manuscript **(Page 2, 5, 12, and 13)**. For example, we have changed "the constructed artificial vision system" to "the simulated artificial vision system", "a real-time color motion detection system is implemented" to "a real-time color motion detection simulation system is implemented", and "by constructing a bioinspired artificial visual system" to "by developing a simulated bionic artificial vision system".

4. Related to point 3, if the system-level demonstrations are simulation-based, the Methods should describe (in a fully reproducible manner) how single-device characteristics (wavelength-dependent response) were mapped onto pixels/cells. It should also be clarified whether D2D variability, read noise, and other non-idealities were included, and how the measured/assumed device responses were incorporated during training and testing.

Response: We thank the reviewer for pointing out this ambiguity. We have now clarified in the manuscript that, for both the static scenario and the dynamic scenario, the system-level demonstrations are based on simulations parameterized by single-device measurements. We have also added a detailed dataset generation workflow in the methods and supplementary information to describe how the measured single device responses were mapped onto simulated pixels and incorporated during training and testing, while accounting for device variability, read noise, and other non-ideal factors. The workflows are summarized in **Fig. R3.**

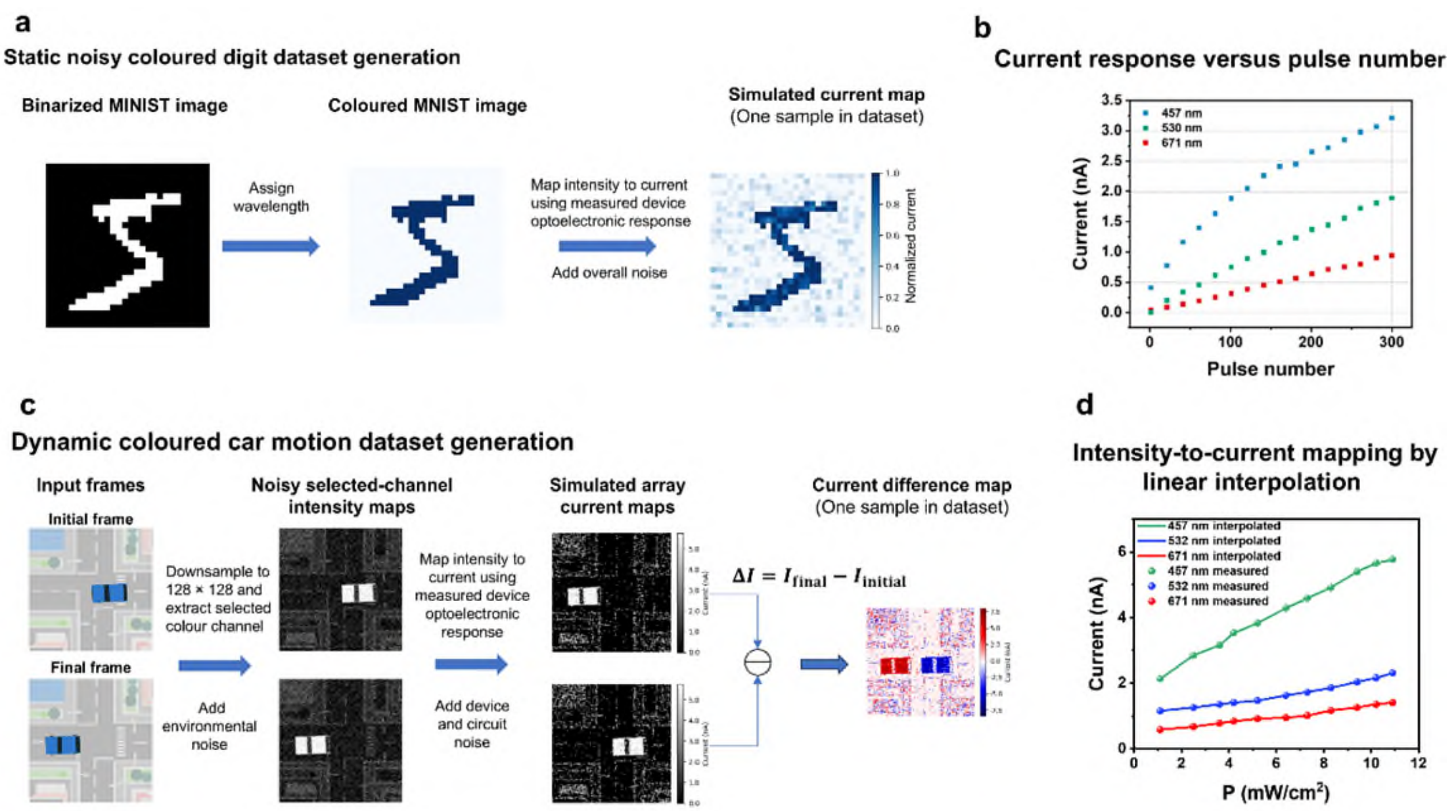

Fig. R3 Workflow for generating simulated dataset samples in the static and dynamic scenarios. a Workflow for generating the noisy colored digit dataset. A binarized digit intensity map was first assigned to one color channel, followed by the addition of an overall Gaussian noise term. This overall noise term represents the combined noise level from possible

environmental, device-related, and circuit-related contributions. The noisy intensity map was then mapped to a simulated array-output current map using the measured device current response versus optical pulse number at RGB-representative wavelengths. **b** Measured current response versus optical pulse number at wavelengths of 457, 532, and 671 nm. **c** Workflow for generating the simulated dynamic colored-car motion dataset. The initial and final frames were downsampled and converted into selected-channel intensity maps. Environmental Gaussian noise was then added to the extracted intensity maps, and the noisy intensity maps were then mapped to simulated array current maps using the measured wavelength- and intensity-dependent single-device response. Because the measured current-intensity response consists of discrete experimental points, linear interpolation was used to construct the wavelength-dependent intensity-to-current mapping function for converting all pixel intensities into simulated device currents. Device noise and circuit related noise were further added to the current maps before inter-frame differencing. The resulting current-difference map was used as one simulated array output sample. **d** Intensity-to-current mapping functions at 457, 532, and 671 nm. The measured discrete current–intensity data points were linearly interpolated to convert pixel intensities into simulated device currents.

For the static scenario, the simulation was designed to evaluate noisy colored digit recognition under different overall noise levels (**Fig. R3a**). The MNIST images have a size of 28 × 28 pixels, corresponding to the simulated 28 × 28 FeFET array. Each digit image was first binarized and then assigned to one color channel, corresponding to red, green, or blue input. The dataset was randomly divided into three equal subsets, and each subset was assigned to a distinct color channel. An overall Gaussian noise term was then added to the image intensity before current mapping. The noisy intensity map was then mapped pixel-by-pixel to a simulated array output current map using the measured device current response versus optical pulse number at RGB-representative wavelengths (**Fig. R3b**). This overall Gaussian noise was used to represent the combined overall noise level in the static dataset, including possible contributions from environmental fluctuations, device variation, and circuit-related uncertainty. The generated current maps were used as the input datasets for training and testing the multi-head convolutional neural network for parallel color and digit recognition.

For the dynamic scenario, the simulation was designed to represent a more realistic dynamic visual process (**Fig. R3c**). For each motion scene, the frame at the beginning of the motion and the frame at the end of the motion were used as the two array input images. First, each input frame was downsampled to match the simulated array size of 128 × 128 pixels. Second, the corresponding color channel intensity was extracted according to the object color. Third, environmental noise was added to the extracted intensity map to represent fluctuations in the optical input image, such as background and illumination variations in the dynamic scene. Fourth, the noisy intensity map was mapped to the array output current map according to the measured wavelength- and intensity-dependent single-device response. Since the measured single-device current response was obtained at a finite number of light-intensity points, linear interpolation was used to construct the wavelength-dependent intensity-to-current mapping function. This mapping function converts each pixel intensity in the selected-channel image into

the corresponding simulated current at the selected wavelength (**Fig. R3d**). During this intensity-to-current mapping step, an additional Gaussian noise was introduced to represent the combined effect of device variation and other circuit-related noise. After this sequence was applied to both the initial and final frames, two simulated array current maps were obtained. The inter-frame difference method, as described in the methods section, was then used to calculate the frame-difference current map. This current difference map corresponds to the simulated array processing result for one dynamic scene and was used as one sample in the dataset. The generated samples were then used for CNN training and testing.

We have added these descriptions to the revised **methods** and included the workflow schematic in the **Supplementary Fig. 27.**

Method

Preparation of datasets for static scenario

For the static-colored digit recognition task, the MNIST dataset was used to generate noisy colored digit datasets. The MNIST images have a size of 28 × 28 pixels, corresponding to the simulated 28 × 28 array. The dataset was randomly divided into three equal subsets, and each subset was assigned to one color channel (red, green, or blue). The dataset contains 60000 training samples and 10000 testing samples.

For each noise level and optical pulse number, an independent dataset was generated with the following sequence (**Supplementary Fig. 27a**). The colored MNIST image was first binarized. An overall Gaussian noise term was then added to each pixel of the binarized input image intensity to simulate the selected overall noise level:

$$P_{noise}(x,y) = P(x,y) + N(0, \sigma_{overall})$$

where $P(x,y)$ is the binarized input image intensity at pixel position (x,y), and $\sigma_{overall}$ is the selected overall noise level. This overall Gaussian noise term represents the combined overall noise level in the static dataset, including possible environmental, device-related, and circuit-related contributions. The noisy image intensity was then mapped to a simulated array output current map using the measured device current response versus optical pulse count across RGB wavelengths (**Supplementary Fig. 27b**). The resulting simulated array output current maps were used for multi-head convolutional neural network training and testing for color and digit recognition.

Preparation of datasets for dynamic scenario

A dynamic colored-car motion dataset was constructed to simulate dynamic visual scenes of a moving vehicle. For each condition, a colored car (red, green, or blue) was generated in synthetic scenes and programmed to move along one of four motion directions (forward, backward, leftward, and rightward), with variations in motion. These rendered scenes formed a 12-class dataset defined by the combination of three colors and four motion directions. Each class contained 100 distinct samples (1200 samples in total), which were split into training and test sets with a 4:1 ratio.

For both the training and test datasets, the same sequence was used to generate the

simulated array output as one dataset sample (**Supplementary Fig. 27c**). For each dynamic scene, the frame at the beginning of the motion and the frame at the end of the motion were used as the two array input images. Each frame was first downsampled to 128 × 128 pixels to match the size of the simulated array. The corresponding color channel was then extracted according to the object color, and the extracted color-channel image was used as optical input for the simulated array. After extracting this selected color-channel image, pixel-wise environmental noise was added to the image:

$$P'_\lambda(x,y) = P_\lambda(x,y) + N(0,\sigma_{\text{env}})$$

where $P_\lambda(x,y)$ is the input image intensity of the selected color channel at pixel position (x,y), $N(0,\sigma_{\text{env}})$ represents the environmental noise, and $P'_\lambda(x,y)$ is the noisy input image intensity. The environmental Gaussian noise was used to represent background and illumination fluctuations in the optical input frames. The current of each simulated pixel was obtained from the measured wavelength- and intensity-dependent device response by linear interpolation:

$$I_0(x,y) = f_\lambda[P_\lambda(x,y)]$$

where $P_\lambda(x,y)$ is the intensity of the selected color channel at pixel position (x,y), f_λ is the linearly interpolated wavelength-dependent intensity-to-current mapping function constructed from the measured discrete data for wavelength λ, (**Supplementary Fig. 27d**) and $I_0(x,y)$ is the mapped device output current.

Device noise and other circuit-related noise were further added to the simulated current maps before inter-frame differencing:

$$I_{\text{sim}}(x,y) = I_0(x,y)[1 + \mathcal{N}(0,\sigma_n)]$$

where σ_n is the normalized standard deviation.

The above procedure was applied both to the initial and final frames, generating two simulated array current maps. The inter-frame difference was then calculated as:

$$\Delta I(x,y) = I_{\text{final}}(x,y) - I_{\text{initial}}(x,y)$$

where $I_{\text{initial}}(x,y)$ and $I_{\text{final}}(x,y)$ are the simulated current maps corresponding to the beginning and end frames, respectively. The resulting current difference map was used as one simulated array output sample for CNN training and testing.

5. The reported energy consumption (~0.4 fJ) is not sufficiently defined. The authors should explicitly state whether the optical input energy is included or excluded. If the estimate accounts only for electrical dissipation, it should be described precisely (for example, "electrical energy consumed in the channel during read/write").

Response: Thank you for your valuable comment. The 0.4 fJ reported in the manuscript refers to the electrical energy consumption during read operations and does not include optical input energy. The energy consumption was calculated using $E = I_{\text{peak}} \times V_{\text{read}} \times t$, where I_{peak}, V_{read}, and t are the peak current, read voltage, and pulse width, respectively. We have corrected the description of energy consumption in the revised manuscript, explicitly stating that this energy consumption refers to "the electrical energy consumed by the channel during read operations." The relevant modification was noted in the revised manuscript **(Page 10)**.

6. The manuscript should more convincingly disentangle optically induced ferroelectric polarization reversal from contributions associated with trapping/photogating. Additional experiments would strengthen this claim, such as (if feasible) comparing PFM domain configurations or polarization hysteresis before/after illumination, and/or employing pulsed electrical measurements designed to minimize trap-related artifacts in transfer characteristics.

Response: Thanks to the reviewer for the comments. Distinguishing the contributions of light-induced ferroelectric polarization reversal and the charge trapping effect is crucial for clarifying the operating mechanism of the device. Given this issue, we supplemented photo-assisted piezoresponse force microscopy (PFM) measurements to directly observe the changes in ferroelectric polarization domains before and after light illumination. As shown in **Fig. R4a**, the polarization states of CIPS and ReS_2 were first set to the downward state by applying a +7 V bias (the red dashed box indicates the ReS_2/CIPS heterojunction, and the black dashed box indicates the separate CIPS). Subsequently, light pulses (457 nm, 10.9 mW/cm^2, and 90 s) were applied, the ferroelectric polarization gradually switched to the upward state **(Fig. R4b)**, confirming light-induced ferroelectric polarization reversal [Nature Communications, 9, 3344 (2018)]. Notably, the polarization reversal in the ReS_2/CIPS heterojunction region was faster and more complete, attributed to the generation of more photogenerated charge carriers in this region. Furthermore, from the electrical transfer characteristics (Fig. R1c), the device exhibits a counterclockwise hysteresis loop dominated by ferroelectric polarization [Nature Electronics, 7, 29-38 (2023); Nature Communications, 12, 1109 (2021)], which is distinctly different from the clockwise hysteresis loop typically dominated by traps. Currently, the results of photo-assisted PFM and electrical transport characteristics indicate that light-induced ferroelectric polarization reversal plays a dominant role. The relevant results have been included in the revised manuscript **(Supplementary Fig. 12 and Page 9)**.

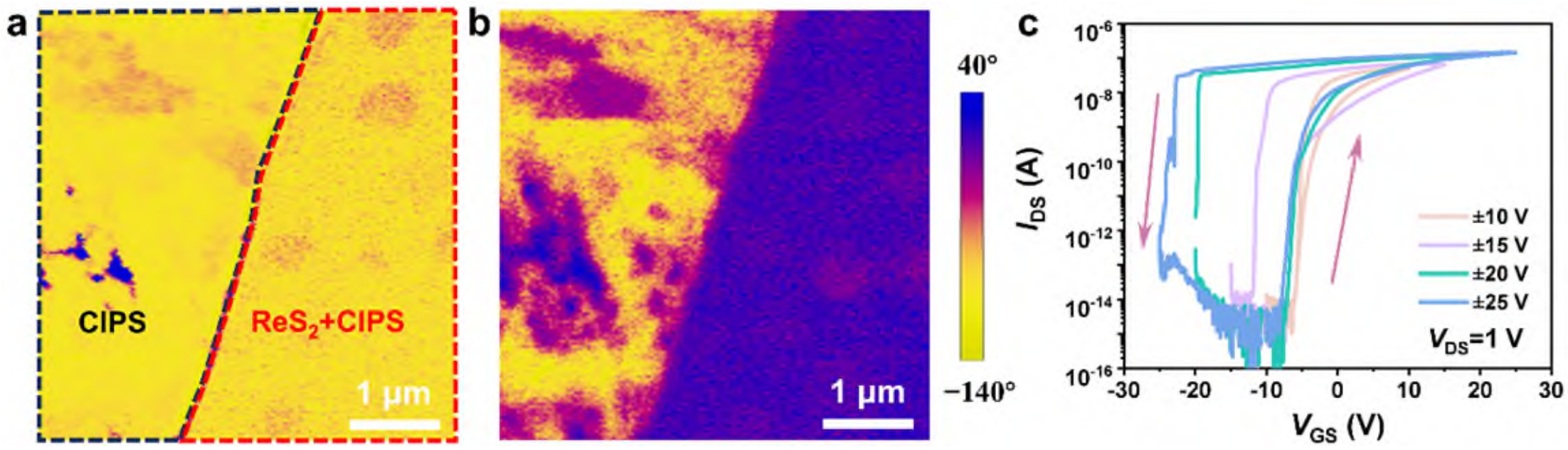

Fig. R4 a, b PFM phase diagrams before and after light-induced ferroelectric domain evolution (457 nm, 10.9 mW/cm^2, 90 s). Scale bar: 1 μm. **c** The transfer curves under different gate-voltage sweep ranges.

7. The "related manuscript" file cannot be opened due to an error, preventing evaluation of the associated content. This needs to be fixed for proper review.

Response: Thank you for the valuable comment. We sincerely apologize for the inconvenience caused by the inability to open the "related manuscript" file. This file

contains the PDF forms required for Nature Communications submission (Editorial Policy Checklist and Lasing Reporting Summary), which need to be opened with Adobe Acrobat Reader. Thank you for your understanding.

Minor comment.

1. In Fig. 2(a), the legend is missing. In addition, the voltage axes should be labeled using standard FET notation (e.g., V_GS, V_DS), since the plotted voltages are not absolute values but bias conditions defined with respect to the relevant terminals.

Response: Thank you for your careful comment. As shown in **Fig. R5**, we have revised Fig. 2a by adding a legend to specify the test conditions for each curve, and modifying the voltage axis labels with standard FET notation (V_{GS} and V_{DS}). The corresponding revisions were updated to the revised manuscript and supplementary information **(Fig. 2a and Page 6)**.

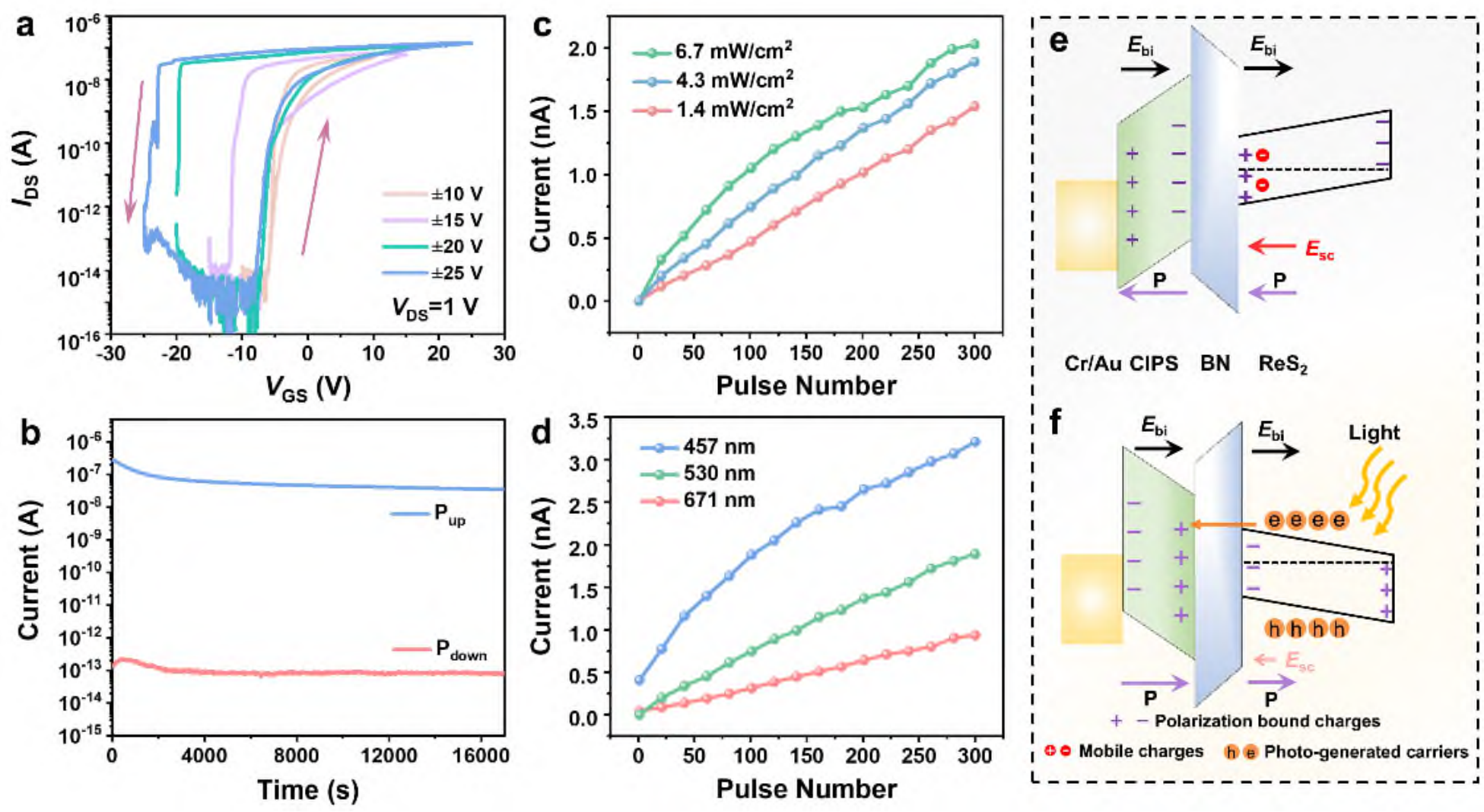

Fig. R5 Memory proprieties and energy band diagrams of the ReS_2/BN/CIPS FeFET. a The transfer curves at different sweep ranges and fixed V_{DS} of 1 V. **b** Retention characteristic after applying different polarization voltage pulses. **c, d** Multi-level memory characteristics with 300 distinguishable conductance states under varying optical conditions: light intensity at a fixed 457 nm wavelength (c) and wavelength at a fixed light intensity (d). **e, f** Band diagrams of light-induced ferroelectric polarization reversal.

Thanks again for your professional comments, which greatly help us improve the manuscript. Hopefully, we have addressed all of your concerns.

Reviewer #2 (Remarks to the Author):

The work presents an opto-electrical ferroelectric memory with demonstrated reconfigurability with potential for applications in visual systems. The authors perform experimental characterization of the devices to demonstrate the functionalities that are essential for neural-network operations, such as LTP, LTD, Retention, etc.
While the overall outlook is interesting, there are few critical details that are vague and unclear. The following points require further clarification.

Response: We thank the reviewer for his/her positive comments on our work. We have carefully considered all comments/suggestions of the reviewer. According to the reviewer's valuable suggestions, the manuscript was revised accordingly as follows.

1. Retention Time:
- One key selling point of the proposed memory is the ability to change the conductance state both optically and electrically.
- The authors demonstrate sufficiently long retention time, but only for electrical operations.
- Authors should demonstrate the long retention time for optical write operations, and at multiple memory states that are achieved through LTP using optical write operations.

Response: We sincerely thank the reviewer for the valuable comments and suggestions. Following the reviewer's comment, we have further supplemented the retention time for optical write operations. First, we applied optical pulses of different durations (100 pulses, 256 pulses) to the device and measured the retention characteristics of the photocurrent. As shown in **Fig R1a**, the photocurrent remained relatively stable even after 10^4 s. Subsequently, we applied 256 consecutive optical pulses to further evaluate the retention characteristics of the multi-level storage states. The results indicate that these 300 conductance states exhibit good retention characteristics, and no significant overlap was observed between adjacent states **(Fig. R1b)**. These results have also been added to the revised supplementary information **(Supplementary Fig. 10)** and the corresponding descriptions have also been added to the revised manuscript on **Page 7**.

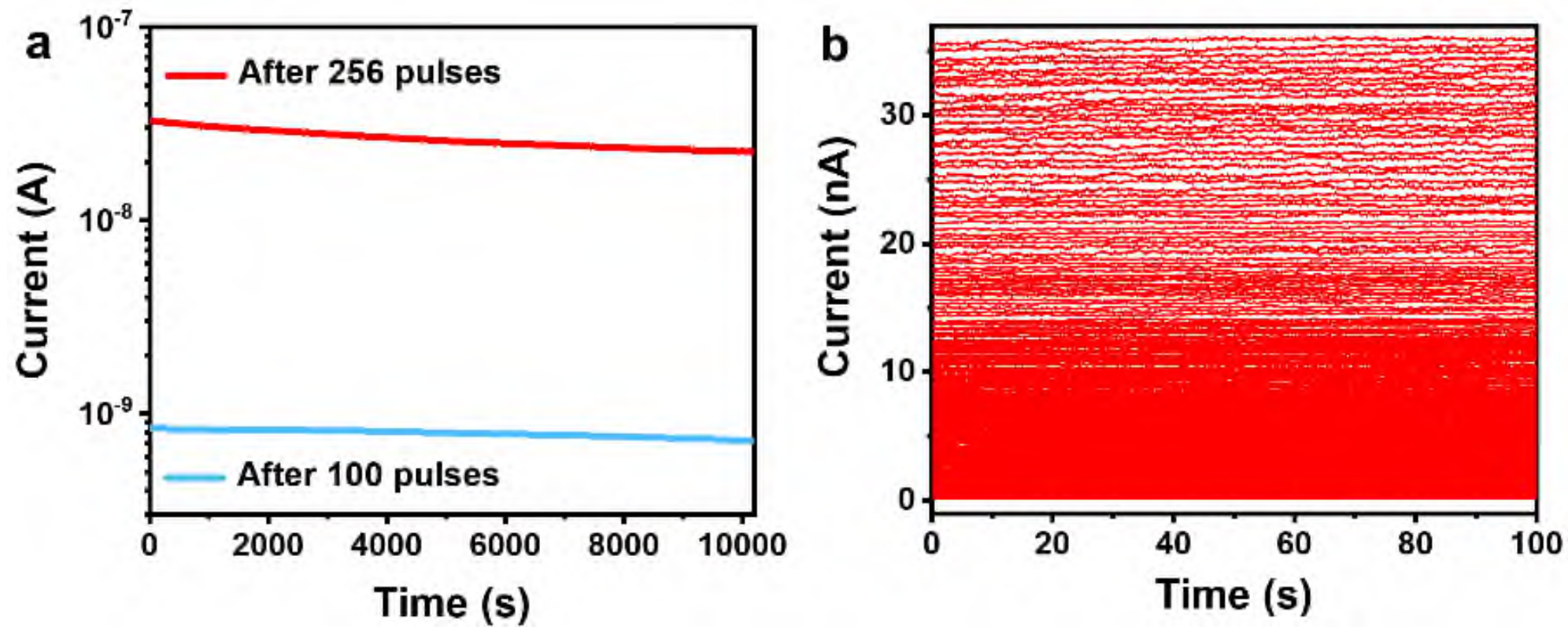

Fig.R1 a Photocurrent retention of the device after 100 and 256 optical pulses. b Photocurrent retention under 256 consecutive optical pulses.

2. Endurance:

- Same as above, that the reported endurance is for electrical write operations, while the key selling point of the device is opto-electric behavior. Therefore, authors should demonstrate endurance for optical operations as well.

Response: Thanks for your suggestion. For cyclic endurance tests of LTP and LTD behaviors under different wavelengths, the LTP process was actually performed under light illumination. Therefore, these endurance data can reflect the durability under optical operation **(Fig. R2)**. In addition, the extensive optoelectronic response tests presented in the manuscript (such as optoelectronic memory and optoelectronic synaptic behaviors) also verify the durability under repeated optical operations.

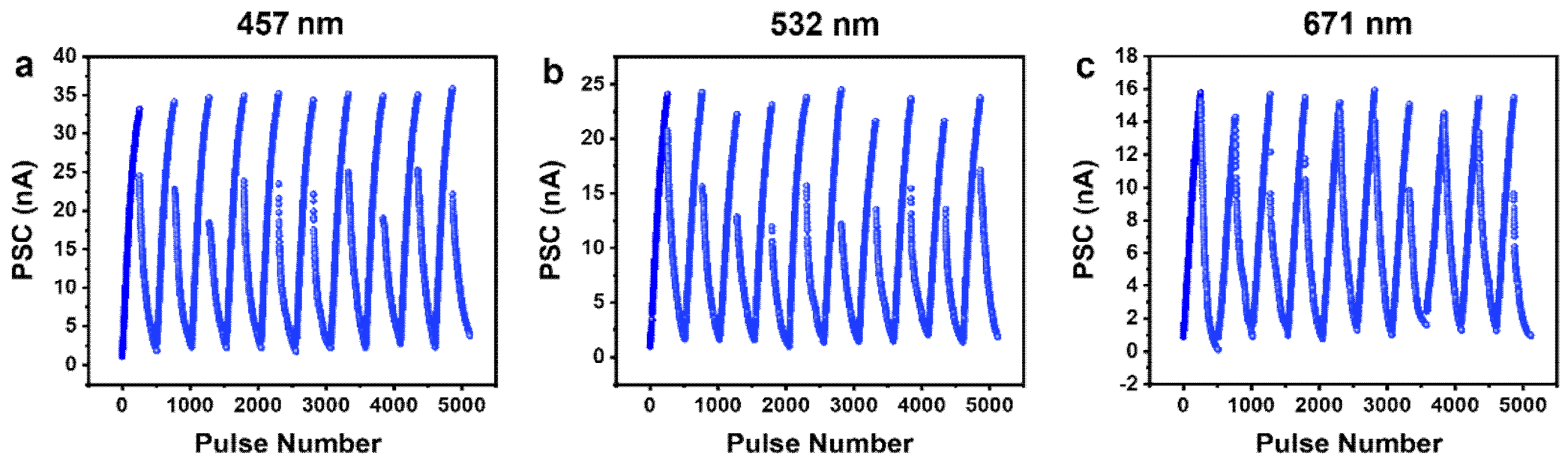

Fig.R2 Cyclic endurance of LTP/LTD behaviors under different wavelengths (457 nm, 532 nm, and 671 nm) and -5 V voltage programming.

3. LTP and LTD:

- The authors demonstrate multi-level capabilities with the LTP and LTD and demonstrate linearity. However, only LTP is performed optically while the LTD is performed electrically.
- Further, authors show polarization reversal only for Pdown to Pup (Line 156), and not the other way around.
- Therefore, authors should clarify if the polarization reverse is possible for program and erase operations.
- Further, authors should explain the implication of this 'limitation' on the overall application. That is, it is likely that a given device may receive optical stimuli while simultaneously receiving an electrical stimulus during write operations. Does this affect anything, as the LTP is optical and LTD is electrical.

Response: Thanks to the reviewer for the insightful and constructive comments. In the original manuscript, we demonstrated optical LTP and electrical LTD to confirm that the device possesses information processing capabilities under both optical and electrical dual-mode modulation. To further illustrate the bidirectional synaptic plasticity of the device under electrical modulation, we have added LTP and LTD behaviors under ±5 V voltages **(Fig. R3)**. The corresponding results and descriptions were added to the revised manuscript and the supplementary information **(Supplementary Fig. 16 and Page 11)**.

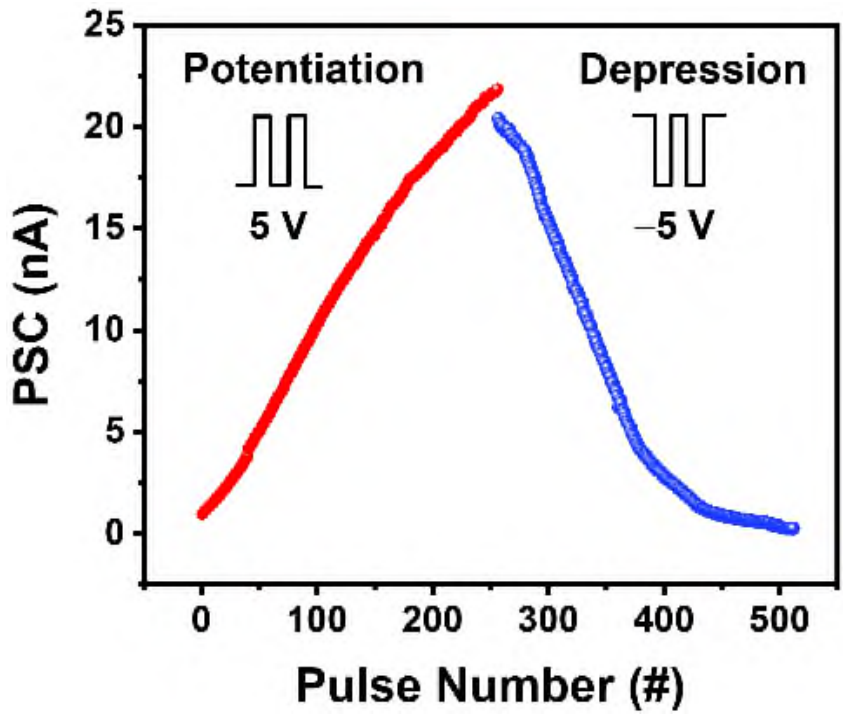

Fig.R3 LTP and LTD behaviors of the device under ±5 V voltage stimulation.

Additionally, Light-induced ferroelectric polarization reversal exhibits directional selectivity. Based on the device structure in this manuscript, light can only drive ferroelectric polarization switching from downward to upward. Specifically, when the ferroelectric polarization is downward, the ReS_2 channel layer is in a high-resistance state, and the photogenerated carriers generated by illumination can neutralize the screening field and depolarization field, thereby inducing polarization reversal from downward to upward. When the ferroelectric polarization is upward, the ReS_2 channel layer is in a low-resistance state, and photogenerated carriers do not induce polarization reversal. This mechanism determines the unidirectionality of light-induced polarization reversal, whereas bidirectional polarization reversal can be achieved through electrical modulation. We have also explicitly clarified that light can only induce unidirectional reversal of ferroelectric polarization in the revised manuscript **(Page 8)**.

Regarding the effect of simultaneous optical and electrical stimulation. If the device receives both optical and electrical stimuli simultaneously, there is a competitive relationship between potentiation and depression. However, in normal operation, LTP and LTD are independently regulated by optical and negative voltage stimulation, respectively, and the appropriate stimulation mode can be selected according to actual needs. Therefore, the scenario of simultaneously applying light and negative voltage stimulation can be avoided in practical applications.

4. Logic Operations:
Authors demonstrate logical AND and OR operations. Authors should provide:
- How the logic gates are constructed
- The context in which the AND and OR operations fit in their end application.
- One input is optical while another is electrical. Why not keep both to be either optical or electrical?
- The magnitude of optical and electrical stimuli strength is different between AND and OR operations. Authors should specify why?
- In the end processing, what would be the source of electrical and optical stimuli? Which physical parameter (e.g. pixel for pattern recognition?) does it correspond to?

Response: Thanks to the reviewer for the detailed suggestions and comments on logic operations. Our point-by-point responses are as follows:

For the construction of logic gates. As shown in Fig. R2, the ferroelectric gate and the channel layer serve as the electrical signal input and optical signal input, respectively, and the source-drain current of the device serves as the output. By setting 0.1 nA as the logic threshold, output current higher than this value is defined as logic "1", and that below this value is defined as logic "0". By applying different combinations of optical and electrical stimuli, "AND" and "OR" logic functions are realized, respectively. Detailed descriptions can be found in Supplementary Fig. 17 and Supplementary Note 3.

"AND" and "OR" logic operations can be applied to application scenarios such as image information preprocessing. For example, logical "OR" can be used to fuse image features, while logical "AND" can extract common features from images, thereby providing a foundation for subsequent high-precision image processing. It should be clearly noted that this manuscript only demonstrates the device's potential in logic operations and we have not further applied it to actual image preprocessing.

The manuscript employs light and electricity as two distinct physical input modes, aiming to demonstrate the information processing capabilities of the device under multi-physical fields modulation. Logical operations typically require two input terminals and an output terminal. In our three-terminal ferroelectric field-effect transistor, optical and electrical dual-mode inputs are employed (light acts on the channel layer, and electricity acts on the ferroelectric gate terminal). These two inputs are independent and do not interfere with each other, thereby allowing the complete realization of the four logical input combinations: (0,0), (0,1), (1,0), and (1,1). If only a single electrical input is used, two independent electrical signals could not be applied simultaneously to the same ferroelectric gate terminal without causing crosstalk. If only a single optical modality is used, two independent light sources are required to act on the channel layer. Therefore, the optical and electrical dual-mode inputs are the ideal solution for achieving complete logic combinations based on this device structure.

In the “AND” and “OR” operations, the difference in the amplitudes of optical and electrical stimuli strength arises from the fact that achieving different logic functions on the single device requires a unified output current threshold. Here, the output current threshold is uniformly set to 0.1 nA. To achieve the logical functions of “AND” and “OR”, the input intensities must be designed differently. Specifically, for the "AND" operation, we employ an optical pulse of 4.3 mW/cm^2 and an electrical pulse of 8 V as inputs. A single input fails to reach the threshold, and only when two inputs are applied simultaneously does the output current exceed the threshold. For the “OR” operation, we use an optical pulse of 10.8 mW/cm^2 and an electrical pulse of 15 V as inputs, a single input is sufficient to cause the output current to exceed the threshold. Therefore, the difference in input intensities is designed to meet the requirements of logical operations under the unified threshold condition.

Our work primarily focuses on implementing “AND” and “OR” logic functions in a single device through optoelectronic synergistic cooperation, without further extending them to specific application scenarios (such as pattern recognition). At the

logic device level, optical stimulation is provided by laser-excited light pulses (457 nm), and electrical stimulation is provided by gate voltage pulses applied using a 4200-semiconductor parameter analyzer.

5. Energy Calculations:
- Authors should provide the power consumption calculations and specify/provide a breakdown of each component.
- Reason being, author require pulses to operate the proposed device, and the peripherals that drive such large voltages can be energy consuming. Clarifying this w/ a breakdown of energy consumption will give a better overall picture.

Response: We thank the reviewer for the valuable suggestions and comments. The total energy consumption comprises two components: external initial programming power consumption and synaptic event power consumption. Figure 3c in the main text and Supplementary Note 1 show the power consumption of the synaptic event (read operation), where the minimum energy consumption per synaptic event is approximately 0.4 fJ. This synaptic power consumption is calculated using the following formula: $E = I_{peak} \times V_{read} \times t$, where I_{peak} (0.08 pA), V_{read} (0.01 V), and t (0.5 s) represent the pulse current, read voltage, and pulse width, respectively. Additionally, the power consumption during the initial programming component is approximately 0.15 pJ, where the read voltage is 5 V, the pulse width is 1 s, and the current is approximately 0.03 pA. To avoid misunderstanding, we have explicitly noted that the consumption (0.4 fJ) corresponds to synaptic events in the revised manuscript **(Page 10)**, not programming power consumption.

6. Operation of Neural Network:
- Array Connection: In which configuration the FeFET array is connected? What are the inputs to the array? How is it connected to the peripherals?
- What is the input both parameter wise and stimuli wise? What is the role of electrical stimuli, optical stimuli?
- Is the network pre-trained with the data-set, or the optical-electrical parameters will be used to train it as well?

Response: We thank the reviewer for raising these important points. The array-level demonstrations in this work are based on simulations parameterized by measured single-device characteristics. To make the system operation clearer, we have added a proposed FeFET array configuration and operation scheme in the revised supplementary information. This added description explains how the optical input is sensed, preprocessed and stored by the FeFET front-end array, how the preprocessed results are electrically read out, how the array is refreshed before the next input, and how the measured device behavior is incorporated into CNN training and testing.

The overall operation workflow is as follows. The optical image is first projected onto the whole FeFET array in parallel. Because the dual-FeFET has wavelength-dependent photoresponse, intensity-dependent photoresponse, optical-pulse-dependent temporal accumulation, and optical memory behavior, each FeFET cell can sense and preprocess the local optical information at its pixel position. The preprocessed optical information

is then stored as a conductance state in each device. After this step, the stored and preprocessed image information is read out electrically. A small read voltage is applied to one selected row, and the currents from different columns are collected to read out this row. By scanning the rows sequentially, the full output conductance map is reconstructed. After the current frame has been read out, a global gate pulse can be applied to refresh the array before the next optical input.

For the array connection (**Fig. R4**), one FeFET cell corresponds to one pixel. The drain, source, and gate terminals are connected separately. The drain terminals are connected row by row to form drain lines. These drain lines are used for row selection and read-voltage application. The source terminals are connected column by column to form source lines. These source lines are used to collect the output currents from the selected row. The gate terminals of all cells are connected to a common gate line. This common gate line is used for global electrical refresh after frame readout.

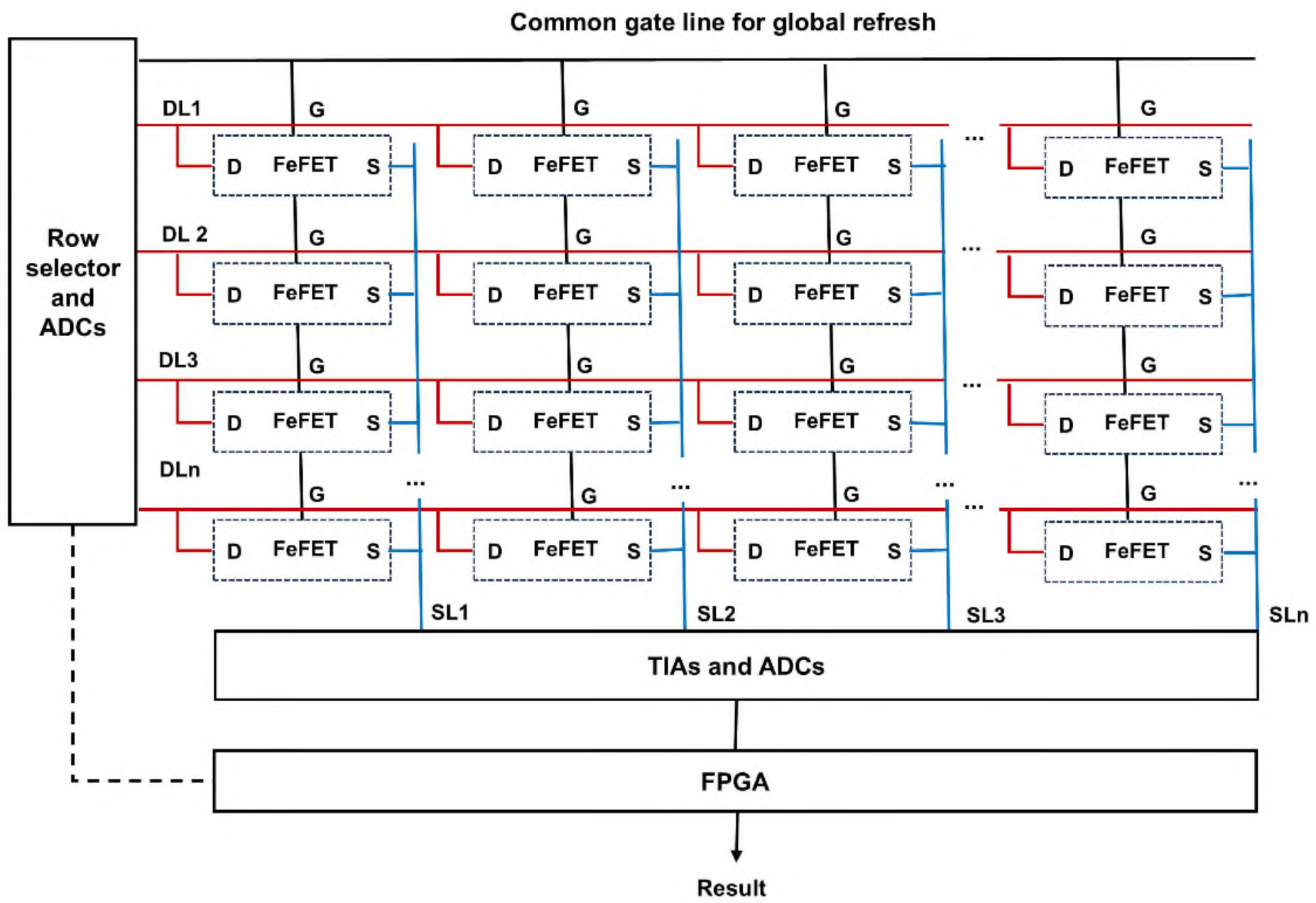

Fig. R4. Proposed connection scheme of the FeFET array and peripheral circuits.

Regarding the peripheral circuits, the drain lines are driven by a row selector and a digital-to-analog converter to provide the read voltage. The source lines are connected to transimpedance amplifiers and analog-to-digital converters for current readout. The common gate line is driven by a digital-to-analog converter to apply the electrical refresh pulse. An FPGA coordinates the overall operation sequence.

The inputs to the array include optical stimuli and electrical stimuli with different roles. The optical stimulus is the raw optical input image. Its parameters include the wavelength or color and light intensity of each pixel. When temporal accumulation is used, the optical pulse number also serves as an input parameter. These optical parameters determine the sensing, preprocessing, and stored conductance state of each FeFET pixel. The electrical stimuli are used for array operation. The small drain read voltage is used to read the stored state from the selected row. The global gate voltage

pulse is used to refresh the array state after the current frame has been read out. This refresh step reduces the influence of the stored information from the previous frame on the next optical input. Thus, in the proposed operation scheme, the optical stimuli on array provide the visual information for front-end sensing and preprocessing, while the electrical stimuli on array provide result readout and global refresh.

We have added the related descriptions in the revised manuscript and Supplementary Information. The array-connection schematic is added in **Supplementary Fig. 28**. In addition, **Supplementary Note 4** describes the array input and operation sequence, including optical sensing, front-end preprocessing, row readout, and global refresh.

Supplementary Note 4. Array input and operation sequence

The FeFET front-end array is designed to sense and preprocess the optical input and store the preprocessed result in the proposed system-level implementation scheme. Each FeFET cell corresponds to one pixel. During optical input, the image is projected onto the whole array in parallel. Owing to the wavelength-dependent photoresponse, intensity-dependent photoresponse, optical-pulse-dependent temporal accumulation, and optical memory behavior of the dual-FeFET, each cell senses and preprocesses the local optical information and stores the preprocessed result as a conductance state.

The array uses separated drain, source, and gate connections (**Supplementary Fig. 28**). The drain terminals are connected row by row as drain lines. During readout, a small read voltage is applied to one selected drain line, and the corresponding row of stored pixel states is read. The source terminals are connected column by column as source lines, and the output currents are converted by transimpedance amplifiers and digitized by analog-to-digital converters. The gate terminals are connected to a common gate line. After one frame is read out, a global gate pulse can be applied through this common gate line to refresh the array state before the next optical input. An FPGA coordinates the overall operation sequence.

In this operation, the optical stimulus is the raw optical input image. Its parameters include the wavelength or color and light intensity of each pixel. When temporal accumulation is used, the optical pulse number also serves as an input parameter. These optical parameters determine the sensing, preprocessing, and stored conductance state of each FeFET pixel. The electrical stimuli are used for array operation. The small drain read voltage is used to read the stored state from the selected row. The global gate voltage pulse is used to refresh the array state after the current frame has been read out. This refresh step reduces the influence of the stored information from the previous frame on the next optical input.

Thanks again for your professional comments, which greatly help us improve the manuscript. Hopefully, we have addressed all of your concerns.

Reviewer #3 (Remarks to the Author):

The authors propose a dual-ferroelectric stacked field-effect transistor that successfully achieves reconfigurable integration of functions including non-volatile memory, synaptic simulation, and mixed-color image processing. The device not only demonstrates outstanding performance in current on/off ratio, multi-level optoelectronic storage, and number of analog states, but also exhibits multi-task parallel recognition capability and high recognition accuracy in handling both static and dynamic mixed-color visual information. However, some issues need to be addressed before publication, as follows.

Response: We thank the reviewer for his/her positive comments on our work. We have carefully considered all comments/suggestions of the reviewer. According to the reviewer's valuable suggestions, the manuscript was revised accordingly as follows.

1. This dual-ferroelectric field-effect transistor demonstrates outstanding performance in non-volatile memory and synaptic plasticity. However, the authors only compared this work with existing studies in terms of current on/off ratio, dark current, number of analog states, and power consumption. How does the high PPF index reported in this manuscript compare to that of recently reported advanced optoelectronic synapse devices?

Response: We thank you for your valuable comment and suggestion. We have added a comparison of the PPF index of our work with that of the state-of-the-art optoelectronic synaptic devices, and the results indicate that the PPF index is at a relatively high level **(Fig. R1)**.

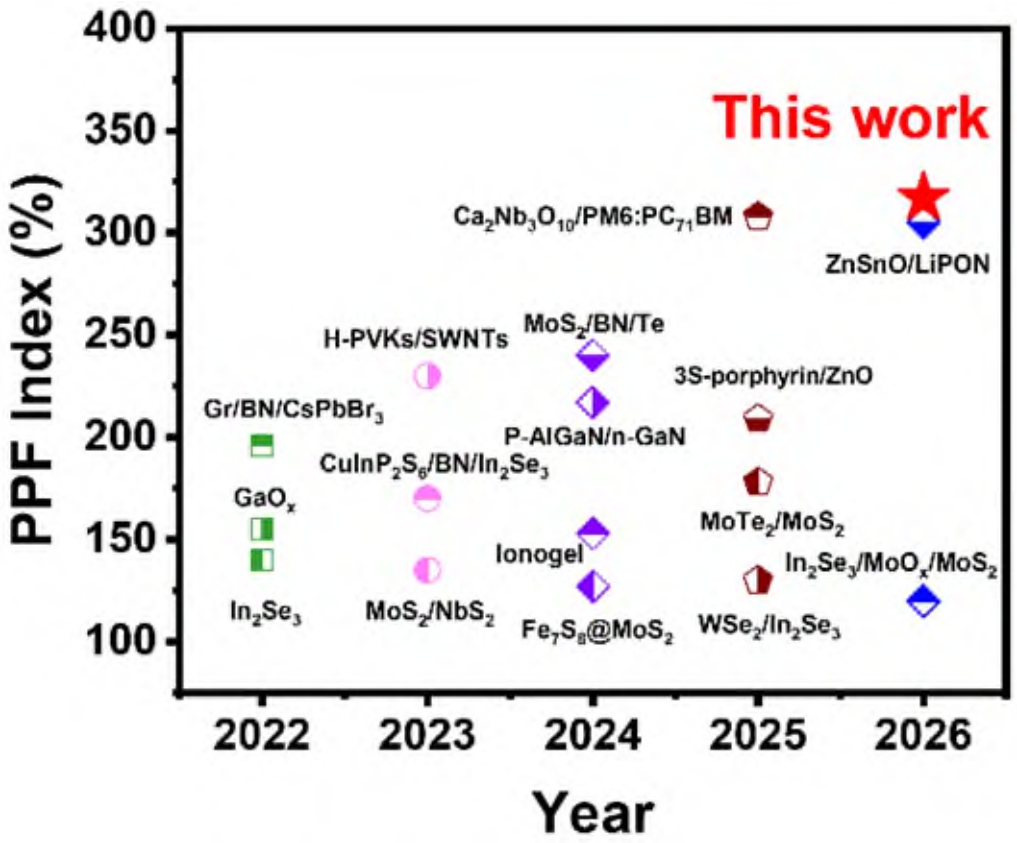

Fig. R1 Comparison of the PPF index of our work with that of other state-of-the-art optoelectronic devices[1-18].

2. The manuscript achieves multi-task parallel processing of static/dynamic mixed-color visual information. Could the authors clarify whether other recent studies also possess similar bio-inspired visual parallel processing capabilities? If so, please provide a comparison in terms of recognition accuracy or functional integration. If not, it is recommended to further highlight the innovation of this work.

Response: We thank the reviewer for their valuable comments. We systematically reviewed the relevant literature and found no existing reports on the multi-task parallel processing of static/dynamic mixed-color visual information. Current studies on mixed-color visual information processing are limited to single tasks such as color or digit recognition, and are unable to perform parallel recognition of both color and digit (or color and motion direction). Therefore, our work overcomes the limitation of existing studies that can only accomplish single recognition tasks and effectively improves information processing efficiency, laying a key foundation for building efficient and compact bio-inspired visual devices. The relevant description has been added to the revised manuscript **(Page 14)**.

3. The performance of the device is highly dependent on its fabrication process. It is recommended that the authors provide a detailed flowchart to illustrate the key fabrication steps.

Response: We sincerely thank the reviewer for this valuable suggestion. We fully agree that the performance of the device is highly dependent on its manufacturing process. We have added a detailed fabrication flowchart of the device **(Fig. R2)**, illustrating the entire process from electrode pre-deposition to dry transfer of materials. Specifically, the device was fabricated using a purely dry process: first, Cr/Au metal electrodes were pre-deposited on a SiO_2/Si substrate via photolithography and electron-beam evaporation; subsequently, CIPS, h-BN, and ReS_2 flakes were mechanically exfoliated from bulk single crystals and sequentially transferred onto the pre-deposited electrodes using the dry transfer technique. This preparation strategy, combining pre-deposited electrodes with dry transfer, effectively avoids the adverse effects of organic solvents on the materials and the heterojunction interfaces during the photolithography process, while also preventing device performance degradation caused by metal infiltration into the materials during electrode fabrication. The relevant fabrication process has been added to the revised supplementary information **(Supplementary Fig. 2)**.

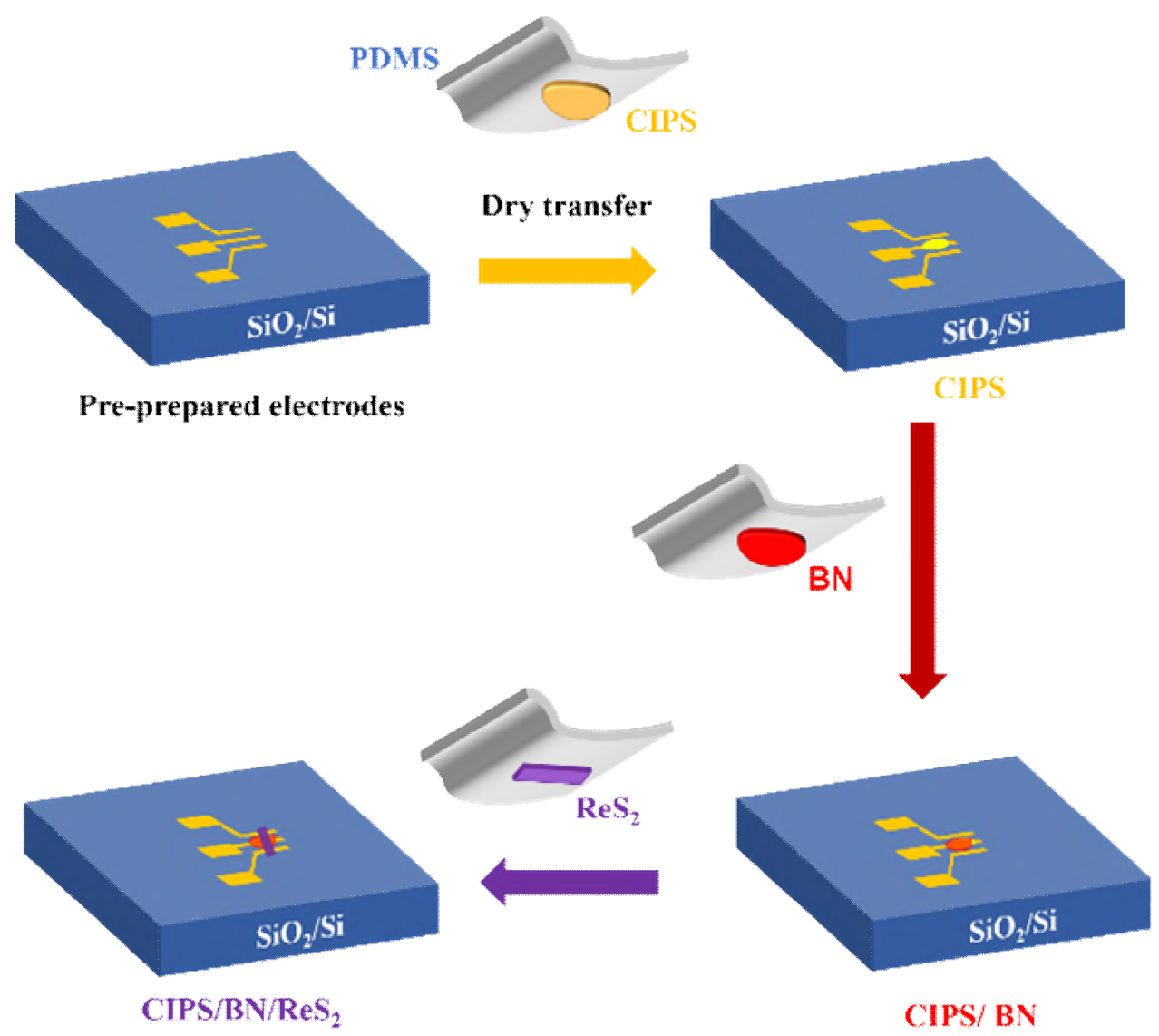

Fig.R2 Fabrication process of the dual-ferroelectric field-effect transistors.

4. In neural network simulations, the linearity and symmetry of weight updates (conductance modulation) directly impact the network's learning efficiency and recognition accuracy. Please quantitatively evaluate the symmetry and linearity of LTP and LTD in Figures 5d-f.

Response: Thank the reviewer for the professional comment and suggestion. We fully agree that the linearity and symmetry of weight updates have a significant impact on the learning efficiency and recognition accuracy of neural networks. The linearity of potentiation and depression is an important factor to evaluate the update of conductance, which is defined by the following equation:

$$G_p = B \cdot \left(1 - exp\left(-\frac{N}{A_p}\right)\right) + G_{min}, \quad (1)$$

$$G_d = -B \cdot \left(1 - \exp\left(-\frac{N\text{-}N_{max}}{A_d}\right)\right) + G_{max}, \quad (2)$$

$$B = \frac{(G_{max} - G_{min})}{\left(1 - e^{-N_{max}/A_{p,d}}\right)} \quad (3)$$

where G_p and G_d are the conductance of potentiation and depression, respectively. A_p and A_d represent the linearity of potentiation and depression, respectively. N and N_{max} are the number and the maximum number of programming pulses, respectively. G_{max} and G_{min} are the maximum and minimum values of conductance.

The linear conductance change between them is evaluated by introducing the asymmetric ratio (*AR*), which is defined by the following equation:

$$AR = \frac{max|G_p(n) - G_d(n)|}{G_p(256) - G_d(256)}, (n = 1 \text{ to } 256) \quad (4)$$

where G_p (n) and G_d (n) are the channel conductance values after applying the n_{th} light pulse and nth electrical spike, respectively.

We quantitatively evaluated the nonlinearity and symmetry of LTP and LTD **(Fig. R3)**. Under modulation with a wavelength of 457 nm and a voltage of -5 V, the A_p/A_d values are approximately 0.77/0.34, and the *AR* is 0.56. For wavelengths of 532 nm and 671 nm, the A_p/A_d values are 1.29/0.39 and 1.99/0.25, respectively, and the *AR* values are 0.45 and 0.52, respectively. The corresponding quantitative analysis results and descriptions were added to the revised manuscript **(Page 10)** and supplementary information **(Supplementary Fig. 14 and Note 2)**.

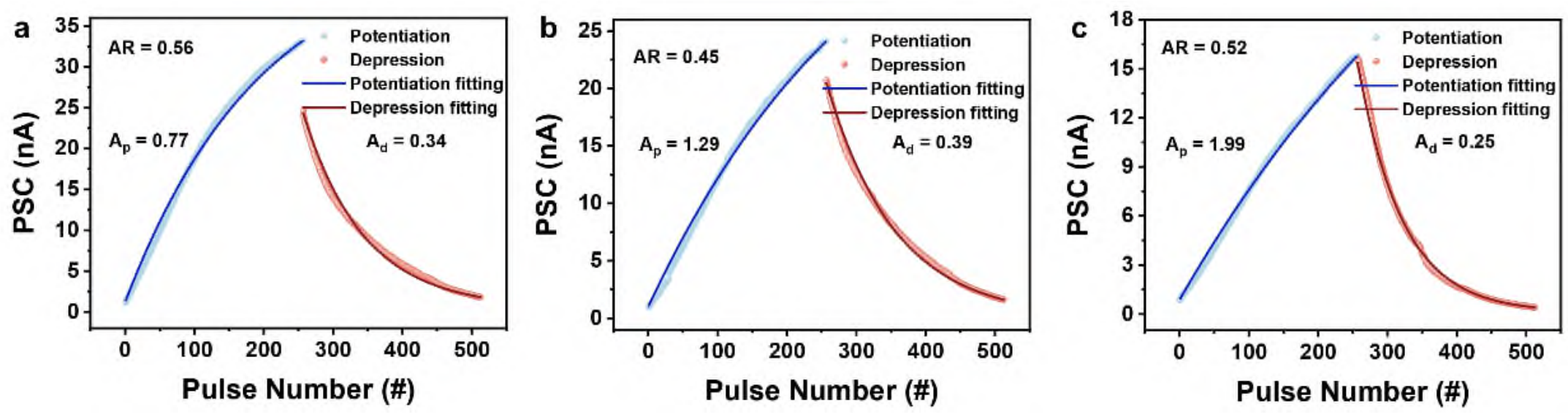

Fig.R3 The Fitted curves for LTP/LTD characteristics and their linearity/symmetry.

5. The CNN architecture diagram shown in Figure 5b does not effectively illustrate the core concept of parallel recognition of color and motion direction.

Response: Thanks to the reviewer for the detailed comment. We have revised the convolutional neural network architecture diagram to clearly illustrate the core concept of parallel recognition of color and motion direction **(Fig. R4 b)**. The corresponding revisions were replaced in **Fig. 5b** of the revised manuscript.

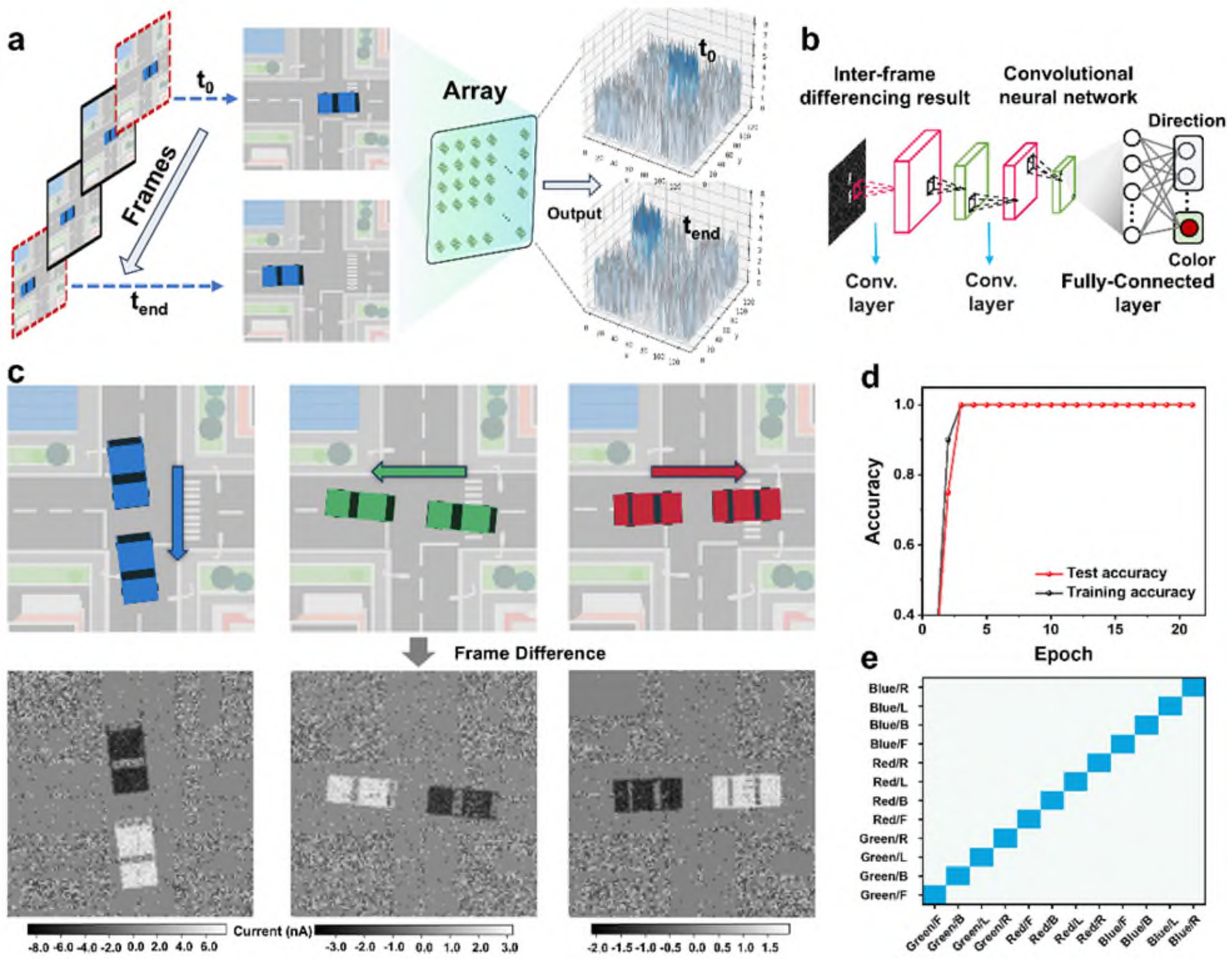

Fig. R4 Color and motion recognition of moving colored targets. Schematic of the neuromorphic dynamic vision system consists of **a** a dual-FeFET array and **b** a CNN network for parallel motion direction and color recognition. **c** Inter-frame differencing results for three colored cars (blue, green, and red) moving in different directions (backward/B, leftward/L, and rightward/R). **d** Recognition accuracy of the network on the training and test sets over training epochs. **e** Confusion matrix of the 12-class classification results on the test set.

Thanks again for your professional comments, which greatly help us improve the manuscript. Hopefully, we have addressed all of your concerns.

References

[1] Han, C. et al. Light-stimulated synaptic transistor with high ppf feature for artificial visual perception system application. *Adv. Funct. Mater.* **32**, 2113053 (2022).

[2] Liu, K. et al. An optoelectronic synapse based on α-In_2Se_3 with controllable temporal dynamics for multimode and multiscale reservoir computing. *Nat. Electron.* **5**, 761–773 (2022).

[3] Zhang, Z. et al. In-sensor reservoir computing system for latent fingerprint recognition with deep ultraviolet photo-synapses and memristor array. *Nat. Commun.* **13**, 6590 (2022).

[4] Das, B. et al. Artificial visual systems fabricated with ferroelectric van der waals heterostructure for in-memory computing applications. *ACS Nano* **17**, 21297-21306 (2023).

[5] Huang, P. Y. et al. Neuro-inspired optical sensor array for high-accuracy static image recognition and dynamic trace extraction. *Nat. Commun.* **14**, 6736 (2023).

[6] Liu, Q. et al. Circular polarization-resolved ultraviolet photonic artificial synapse based on chiral perovskite. *Nat. Commun.* **14**, 7179 (2023).

[7] Deng, Y. et al. Intrinsic defect-driven synergistic synaptic heterostructures for gate-free neuromorphic phototransistors. *Adv. Mater.* **36**, 2309940 (2024).

[8] Liu, X. et al. Optoelectronic synapses with chemical-electric behaviors in gallium nitride semiconductors for biorealistic neuromorphic functionality. *Nat. Commun.* **15**, 7671 (2024).

[9] Luo, X. et al. A bionic self-driven retinomorphic eye with ionogel photosynaptic retina. *Nat. Commun.* **15**, 3086 (2024).

[10] Zha, J. et al. A 2D heterostructure-based multifunctional floating gate memory device for multimodal reservoir computing. *Adv. Mater.* **36**, 2308502 (2024).

[11] Zhao, Z. et al. Bias-switchable photodetection and photosynapse dual-functional devices based on 2D perovskite/organic heterojunction for imaging-to-recognition conversion. *Adv. Mater.* **37**, 2416033 (2024).

[12] Li, J. et al. Phase-engineered In_2Se_3 ferroelectric p-n junctions in phototransistors for ultra-low power and multiscale reservoir computing. *ACS Nano* **19**, 13220-13229 (2025).

[13] Ouyang, Y. et al. Gate-tunable dual-mode optoelectronic device for self-powered photodetector and optoelectronic synapse. *Adv. Sci.* **12**, 2416259 (2025).

[14] Su, F., Wan, J. & Wang, D. Bioinspired fully optical modulated artificial visual synapse with outstanding memory retention based on hollow multishelled structure. *Adv. Funct. Mater.* **35**, 2425627 (2025).

[15] Talanti, S. et al. CMOS-integrated organic neuromorphic imagers for high-resolution dual-modal imaging. *Nat. Commun.* **16**, 4311 (2025).

[16] Yang, D. et al. Relaxor antiferroelectric dynamics for neuromorphic computing. *Adv. Mater.* **37**, 2419204 (2025).

[17] Xie, L. et al. Brain-inspired cmos-compatible ZnSnO synaptic array with ultra-high PPF for versatile neuromorphic computing. *Adv. Funct. Mater.*, e30738 (2026) https://doi.org/10.1002/adfm.202530738.

[18] Zhi, J. et al. Ferroelectricity-modulated asymmetric van der waals heterostructure for ultralow-power neuromorphic synapse and logic-in-memory operations. *Nat. Commun.*, **17**, 3974 (2026).

List of Responses and Revision

Title: "Dual-ferroelectric stacked high-performance field-effect transistor"

ALL THE CHANGES WERE DONE IN RED IN THE REVISED MANUSCRIPT.

REVIEWER COMMENTS

Reviewer #1 (Remarks to the Author):

The authors have addressed most of the technical concerns raised in the previous round, particularly regarding device reliability, multilevel-state retention, the distinction between measured single-device data and simulation-based array demonstrations, and the mechanism of light-induced ferroelectric polarization reversal. I still have some reservations about the overall novelty and contribution of the manuscript, since the array-level demonstrations remain simulation-based rather than experimentally implemented. However, I leave this broader editorial judgment to the editor. From a technical standpoint, the revision is substantially improved, and only the following minor points remain.

Response: We sincerely thank the reviewers for acknowledging the technical improvements in our revised manuscript. Regarding the reviewer's concern that the array-level demonstrations remain simulation-based rather than experimentally implemented, we completely agree that a fully hardware-based array demonstration is the most convincing form of verification for practical applications.

Nevertheless, at the current stage of our research, full array integration has not yet been completed due to the process complexity involved in stacking multiple materials. It should be emphasized that our array-level demonstrations are not built upon theoretical assumptions, but rather on extensive experimental data measured from single-device. This experiment-data-driven system-level simulation enables us to reasonably estimate array performance and verify architectural feasibility. We fully understand the reviewer's concern regarding the limitations of simulations. Therefore, we provide the following detailed explanation and response to clarify the overall novelty and contribution of our work.

Most existing optoelectronic synapse devices typically lack functional reconfigurability and the ability of parallel processing of multidimensional visual information (such as color, shape, and motion), which severely limits their application potential in task-adaptive switching and multi-source information fusion perception. Therefore, two aspects need to be explored and developed: firstly, the task-adaptive functional reconfiguration capability, enabling dynamic switching of processing modes based on actual needs; secondly, the parallel processing capabilities and recognition accuracy for static and dynamic mixed-color visual information.

To this end, we report a dual-ferroelectric stacked transistor that enables the reconfigurable functional integration of electrical memory, multilevel optical memory, and visual function

simulation within a single device. The dual-ferroelectric architecture synergistically enhances memory performance and synaptic plasticity, including an ultra-high on/off ratio, ultra-low dark current, >8-bit conductance states, and long-term potentiation/depression (LTP/LTD) with a wide dynamic range. These device characteristics compare favorably with previously reported state-of-the-art devices across multiple key performance metrics, and achieve the integration of high performance and multifunctionality. Building upon the experimentally demonstrated device characteristics, the system-level simulations demonstrate multi-task parallel processing of mixed-color static and dynamic visual information, enabling accurate multitask recognition of color-number or color-motion direction combinations beyond conventional single-task perception, and significantly improving both information processing efficiency and accuracy.

Overall, our work not only achieves the integration and performance enhancement of multimodal functions but also verifies its efficient parallel processing capabilities in complex visual scenarios, providing a new perspective for developing next-generation energy-efficient, compact neuromorphic vision chips. Although the current array-level demonstrations are simulation-based, the experimental validation of the underlying device together with the system-level simulations provides a solid proof of concept for the proposed architecture and establishes a practical foundation for future experimental implementation of neuromorphic vision arrays.

We are again grateful to the reviewers for the constructive feedback, and hope that the above explanation will better highlight the novelty and contributions of the manuscript.

1. The energy-consumption claim should be further qualified. The reported value of ~0.4 fJ refers only to the electrical energy consumed in the channel during read operation and excludes the optical input energy as well as the initial polarization-programming energy. This limitation should be stated consistently in the main text, figure caption, and any summary statements, so that the value is not interpreted as the total device- or system-level energy consumption.

Response: Thanks to the reviewer for the suggestions. We sincerely apologize for the imprecise description of the energy consumption in our initial manuscript. The reported value of ~0.4 fJ is derived solely from the electrical energy consumption in the channel during the read operation, and it does not account for the optical input energy and polarization-programming energy.

To avoid any misunderstanding, we have thoroughly revised the entire manuscript to explicitly state this limitation. The corrections can be found at the following locations:

In the **Introduction**, we have revised the description to: "...and electrical energy consumption of about 0.4 fJ (excluding the optical input and the initial polarization-programming energy consumption)".

In the **Results** (the section describing Figure 3c), we have revised the description to: "...the minimum electrical energy consumption in the channel during read operation per synaptic event is about 0.4 fJ (excluding the optical input and initial polarization-programming energy consumption) (Supplementary Note 1)".

In the **Figure 3c caption**, we have updated the description to: "…the minimum electrical energy consumption of about 0.4 fJ per pulse (excluding optical input and initial polarization-programming energy consumption)".

2. The added photo-assisted PFM data provide useful support for light-induced ferroelectric polarization reversal. However, these results do not completely exclude possible contributions from trapping or photogating effects, especially under the actual pulsed optical conditions used for synaptic operation. The authors should therefore avoid overly strong mechanistic language and describe ferroelectric polarization reversal as the dominant or primary contribution, rather than the sole mechanism.

Response: We thank the reviewer for the careful suggestions. We completely agree that while the photo-assisted PFM data provide useful support for light-induced ferroelectric polarization reversal, they cannot completely exclude possible contributions from trapping or photogating effects.

Following the reviewer's suggestion, we have carefully refined the description of the mechanism to ensure scientific rigor. Specifically, we made the following revisions to the manuscript:

To verify the mechanism of light-induced ferroelectric polarization reversal, we conducted photo-assisted PFM measurements to directly observe the changes in ferroelectric polarization domains before and after light illumination. As shown in Supplementary Fig. 12a, the polarization states of CIPS and ReS_2 were first set to the downward state by applying a +7 V bias (the red dashed box indicates the ReS_2/CIPS heterojunction, and the black dashed box indicates the separate CIPS). Subsequently, a light pulse (457 nm, 10.9 mW/cm^2, and 90 s) was applied, the ferroelectric polarization gradually switched to the upward state (Supplementary Fig. 12b), indicating that light illumination can effectively induce ferroelectric polarization reversal. Notably, the polarization reversal in the ReS_2/CIPS heterojunction region was faster and more complete, which is predominantly attributed to the generation of more photogenerated charge carriers in this region. Although photo-assisted PFM data provide useful support for light-induced ferroelectric polarization reversal, it should be noted that these results do not completely exclude possible contributions from trapping or photogating effects, especially under the actual pulsed optical conditions used for synaptic operation.

Reviewer #3 (Remarks to the Author):

All revisions have been completed. Recommended for publication.

Response: Thank you very much for your positive comments. Your valuable feedback has significantly contributed to the improvement of our work.

Reviewer #4 (Remarks to the Author):

I co-reviewed this manuscript with one of the reviewers who provided the listed reports. This is part of the Nature Communications initiative to facilitate training in peer review and to provide appropriate recognition for Early Career Researchers who co-review manuscripts.

Response: Thank you very much for your participation in the peer review process. We appreciate the collaborative effort in reviewing our manuscript and the valuable insights provided.

List of Responses and Revisions

Title: "Dual-ferroelectric stacked high-performance field-effect transistor"

ALL THE CHANGES WERE DONE IN RED IN THE REVISED MANUSCRIPT.

Reviewer #1 (Remarks to the Author):

Comments:

The authors have adequately addressed the remaining comments and concerns.

Response: Thank you very much for your positive comments. Your valuable feedback has significantly contributed to the improvement of our work.

Reviewer #4 (Remarks to the Author):

Comments:

I co-reviewed this manuscript with one of the reviewers who provided the listed reports. This is part of the Nature Communications initiative to facilitate training in peer review and to provide appropriate recognition for Early Career Researchers who co-review manuscripts.

Response: Thank you very much for your participation in the peer review process. We appreciate the collaborative effort in reviewing our manuscript and the valuable insights provided.
